# Structure-guided development of a potent human B^0^AT1 inhibitor effective in a mouse model of phenylketonuria

**DOI:** 10.1038/s42003-026-10535-y

**Published:** 2026-06-25

**Authors:** Takuya Imazu, Tomoya Akashi, Masahiro Hiraizumi, Yosuke Inui, Wataru Sasaki, Tsuyoshi Takahashi, Hidenori Todoroki, Taichi Kumanomidou, Kazunori Yamada, Norie Fujikawa, Hiromi Hisano, Hidetsugu Asada, Tsukasa Kusakizako, Tomohiro Nishizawa, So Iwata, Osamu Nureki, Ikuko Miyaguchi

**Affiliations:** 1 Neuro Science Unit Sohyaku Innovative Research Division, Mitsubishi Tanabe Pharma Co., Ltd., Yokohama, Japan; 2 DMPK Research Laboratories Sohyaku Innovative Research Division, Mitsubishi Tanabe Pharma Co., Ltd., Yokohama, Japan; 3 Department of Chemistry and Biotechnology, Graduate School of Engineering, The University of Tokyo, Tokyo, Japan; 4 Discovery Technology Laboratories Sohyaku Innovative Research Division, Mitsubishi Tanabe Pharma Co., Ltd., Yokohama, Japan; 5 Department of Cell Biology, Graduate School of Medicine, Kyoto University, Kyoto, Japan; 6 Department of Biological Sciences, Graduate School of Science, The University of Tokyo, Tokyo, Japan; 7 Graduate School of Medical Life Science, Yokohama City University, Yokohama, Japan

**Keywords:** Cryoelectron microscopy, Structure-based drug design

## Abstract

B^0^AT1 (SLC6A19) is a neutral amino acid transporter mediating intestinal absorption and renal reuptake of amino acids, including phenylalanine (Phe). Inhibiting B^0^AT1 enhances Phe excretion, offering a therapeutic strategy for phenylketonuria (PKU). Using cryo-EM, we determined human B^0^AT1 structures in outward- and inward-open states, revealing an allosteric pocket ~17 Å from the substrate site that is present in the outward-open conformation and has been previously reported. Structure-guided inhibitor design targeting this pocket produced a potent B^0^AT1 inhibitor that locks the transporter in an outward-occluded state and blocks transport. The higher-resolution structures reveal detailed interactions at the binding site, including water-mediated coordination and conformational changes around Leu52. This inhibitor exhibited submicromolar IC_50_ against both human and mouse B^0^AT1, and oral administration in PKU model mice increased urinary Phe and reduced plasma Phe levels. These findings provide structural insight into allosteric inhibition of B^0^AT1 and establish a framework for the rational optimization of inhibitors targeting conformationally dynamic allosteric sites in SLC6-family transporters.

## Abstract (web summary)

High-resolution structures of human B0AT1 reveal an allosteric inhibition mechanism and enable the development of potent inhibitors that reduce phenylalanine levels in a mouse model of phenylketonuria.

## Introduction

Phenylketonuria (PKU) is a recessive genetic disorder caused by deficiency of phenylalanine hydroxylase (PAH), leading to phenylalanine (Phe) accumulation and severe neurodevelopmental impairment. Elevated Phe disrupts brain amino acid homeostasis, resulting in hypomyelination, gliosis, and neurotransmitter deficits^1,2^. Although drugs like sapropterin and pegvaliase are approved for PKU, they are ineffective in many cases, due to genetic heterogeneity. Thus, there remains a substantial unmet need for broadly effective therapies to lower blood Phe levels across diverse patient populations^3^. We therefore sought to lower systemic Phe levels by modulating its transport in peripheral organs and targeted B^0^AT1 (SLC6A19) to reduce the plasma Phe levels in patients with PKU.

B^0^AT1 is a key transporter that mediates absorption of neutral amino acids, including Phe, in the intestine and reabsorption in the kidney. Inhibition of B^0^AT1 function in the kidney is expected to block Phe reabsorption into the blood, promote its urinary excretion, and lower its plasma levels^4–6^. B^0^AT1 is a member of the SLC6 transporter family, which includes serotonin transporter (SERT; SLC6A4), IMINO transporter (SIT1; SLC6A20), and dopamine transporter (DAT1; SLC6A3)^7–9^. The activity of B^0^AT1 depends on the co-expression of Tmem27 (Collectrin) in the kidneys and angiotensin-converting enzyme 2 (ACE2) in the intestines^10^. Similar dependencies are observed for other neutral amino acid transporters, such as B^0^AT3 and SIT1^10,11^. The B^0^AT1-ACE2 complex is also known as a receptor for coronaviruses^12^. Co-expression of human ACE2 or Collectrin is known to increase transporter function by approximately tenfold^13,14^.

**Fig. 1:**
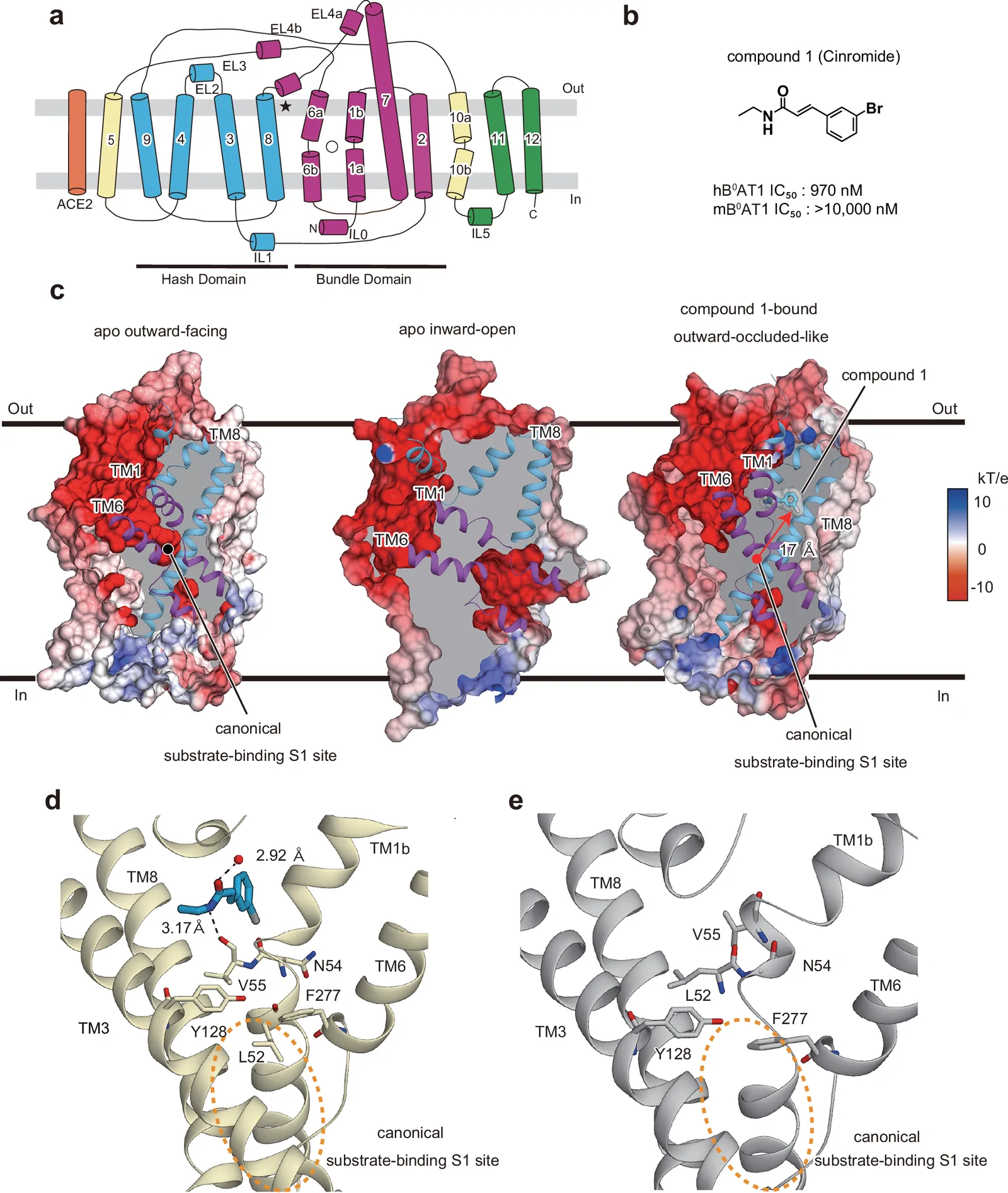
Cryo-EM structures of the human B^0^AT1 in the three states. **a** Topological diagram of the B^0^AT1-ACE2 heterodimer, showing the location of the canonical substrate-binding S1 (〇) and compound binding (★) sites. **b** Structure of compound 1 (Cinromide), a B^0^AT1 inhibitor, with IC_50_ values for human and mouse B^0^AT1. **c** Cross-sectional electrostatic surface potentials of apo outward-facing B^0^AT1, apo inward-open B^0^AT1, and the B^0^AT1-compound 1 complex (outward-occluded-like). **d** The compound binding site and its surrounding structure in the B^0^AT1-compound 1 complex. **e** The same site of apo outward-facing B^0^AT1. The gate formed by Y128 and F277 is conserved in both structures. Symmetry expansion and focused classification were applied to improve local resolution around the inhibitor-binding site, enabling clear visualization of compound 1 density.

B^0^AT1 has a leucine transporter-like (LeuT) fold structure common to SLC5, 6, and 7 families, with ten α-helices in TM1-10 (Fig. 1a). The function and structure of these transporter families have been extensively studied in the LeuT and serotonin transporter (SERT) of SLC6^7,15^. The structure of LeuT consists of a bundle domain of four α-helices (TM1, 2, 6, and 7) and a hash domain of another four α-helices (TM3, 4, 8, and 9), with gate helices TM5 and 10. These transporter classes utilize sodium ions (Na^+^) as a co-substrate for active transport. Extracellular Na^+^ binding induces a conformational change in the hash domain, shifting the transporter to an outward-facing conformation. This allows for substrate binding, followed by a transition to an inward-facing conformation, simultaneously translocating both the substrate and Na^+^ into the cell. The stoichiometry of Na^+^ binding varies, ranging from one to three ions, and some transporters also require chloride ions (Cl^-^) for function. This transport cycle, involving alternating outward and inward conformations, is known as the alternating access mechanism^16^. Although the amino acids at the Na1 and Na2 sites are commonly found in SLC6s, as previously reported^16^, B^0^AT1 transports substrates in a 1:1 ratio with Na⁺, independent of Cl⁻^17,18^.

Several B^0^AT1-ACE2 structures^8,19,20^ have been reported, revealing the substrate-binding site adjacent to the conserved unwound regions in TM1 and TM6, which exhibits structural flexibility that favors the recognition of multiple substrates^21^. Although an allosteric binding mode for Cinromide analogs was recently reported^20^, its functional role had not been fully elucidated. Previous cryo-EM studies have identified an allosteric site located between TM6 and TM8 and demonstrated that inhibitor binding stabilize the outward-open conformation to prevent the transition to the inward conformation^20^. Importantly, this site is distinct from both the canonical substrate-binding S1 site and the S2 site of SERT, which is often referred to as an allosteric site and accommodates inhibitors like escitalopram and duloxetine^22^. Cinromide analogs bind to a unique, previously uncharacterized site specific to B^0^AT1, topologically separate from any known ligand-binding pockets in other SLC6 transporters.

In contrast to the relatively small and conserved S1 site, allosteric sites offer a more promising avenue for developing specific inhibitors^5^. However, in vivo efficacy of B^0^AT1 allosteric inhibitors has been reported, although the structural basis for their activity and optimization remains incompletely understood. Successful drug development necessitates demonstrating efficacy in animal models and humans, bridging the gap between in vitro and in vivo studies. This requires iterative compound optimization to achieve target protein efficacy across species.

In this study, we developed inhibitors that specifically bind to an allosteric site in B^0^AT1 and demonstrated that these inhibitors reduce plasma Phe concentrations in vivo in PKU model mice. Cryo-EM structures of multiple conformations revealed that this site is exclusive to the outward-open conformation and that binding of the compounds induces an outward-occluded conformation without substrate binding. These insights pave the way for the development of therapeutics targeting this allosteric site for the treatment of PKU.

## Results

### Sample preparation and structural characterization of B^0^AT1 in apo and Cinromide-bound forms

Human wild-type B^0^AT1 (encoded by SLC6A19) was unstable when expressed alone. To address this, we stabilized the protein through complex formation with ACE2, a strategy commonly used in previous structural studies of B^0^AT1 and related transporters^19,20^, and with Collectrin, or by introducing stabilizing mutations and antibody Fab fragments. We selected Cinromide (compound 1; Fig. 1b), a known potent B^0^AT1 inhibitor^23–25^, as an initial compound for structural analysis. The stabilized B^0^AT1 construct (sB^0^AT1) was engineered for structural studies and was not used for functional transport assays, which were performed using wild-type B^0^AT1. In previously reported cryo-EM structures, ACE2 forms a homodimer that recruits two B^0^AT1 molecules, resulting in a dimeric ACE2-B^0^AT1 complex^19^. Consistent with these reports, all ACE2-bound particles in our dataset were reconstructed as dimers. In contrast, the Fab-bound complex was observed as a monomeric particle, reflecting the absence of ACE2-mediated dimerization. To improve the local resolution around the inhibitor-binding site, symmetry expansion followed by focused classification and local refinement was performed, enabling high-resolution visualization of the ligand-binding region at the monomer level. Cryo-EM analysis of B^0^AT1 in complex with ACE2 or Fab yielded three distinct conformations: an outward-facing apo structure (ACE2 complex), an inward-open apo structure (Fab complex), and an outward-occluded-like closed structure bound to compound 1 (ACE2 complex). The outward-facing apo structure was consistent with previous reports^19^. Notably, we identified a sodium ion (Na^+^) in the Na2 site, coordinated by five neighboring atoms with distances of ~2.4 Å, consistent with previously reported Na2 coordination geometry in SLC6 transporters (Supplementary Fig. 1a). In contrast, the inward-open apo structure lacked a density for Na⁺ at the Na2 site (Supplementary Fig. 1b). No density was detected at the putative Na1 site in either apo structure (Supplementary Fig. 1c, d), likely reflecting the absence of substrate^17^.

The compound 1 bound structure adopts an outward-occluded-like conformation, with a closed intracellular gate formed by Tyr128 and Phe277, in the absence of substrate (Fig. 1c, d). In this conformation, Na⁺ was not observed at either the Na1 or Na2 sites (Supplementary Fig. 2a, b). Compound 1-bound to an extracellular allosteric site ~17 Å from the canonical substrate-binding site (Fig. 1c–e). Its binding induced a shift of TM1b toward the canonical substrate-binding site, enabling a hydrogen bond between the amide NH of compound 1 and the carbonyl oxygen of Val55. The amide carbonyl of compound 1 also binds to a water molecule (Fig. 1d and Supplementary Fig. 2c). In the compound 1-bound structure, Leu52 flipped toward the canonical substrate-binding site, effectively occluding the cavity. In the previously reported structure of the inhibitor JX98, a hydrogen bond was formed with Trp56, and a similar Leu52 flipping was observed^20^. However, unlike compound 1, it lacked the water-mediated interaction (Supplementary Fig. 3). For compound 1, consistent with its binding mode, we observed potent inhibition of human B^0^AT1 (hB^0^AT1: IC_50_ = 970 nM), whereas inhibition of mouse B^0^AT1 (mB^0^AT1) was much weaker (>10,000 nM) (Fig. 1b).

### Optimized compounds and their improved in vitro activities

**Fig. 2:**
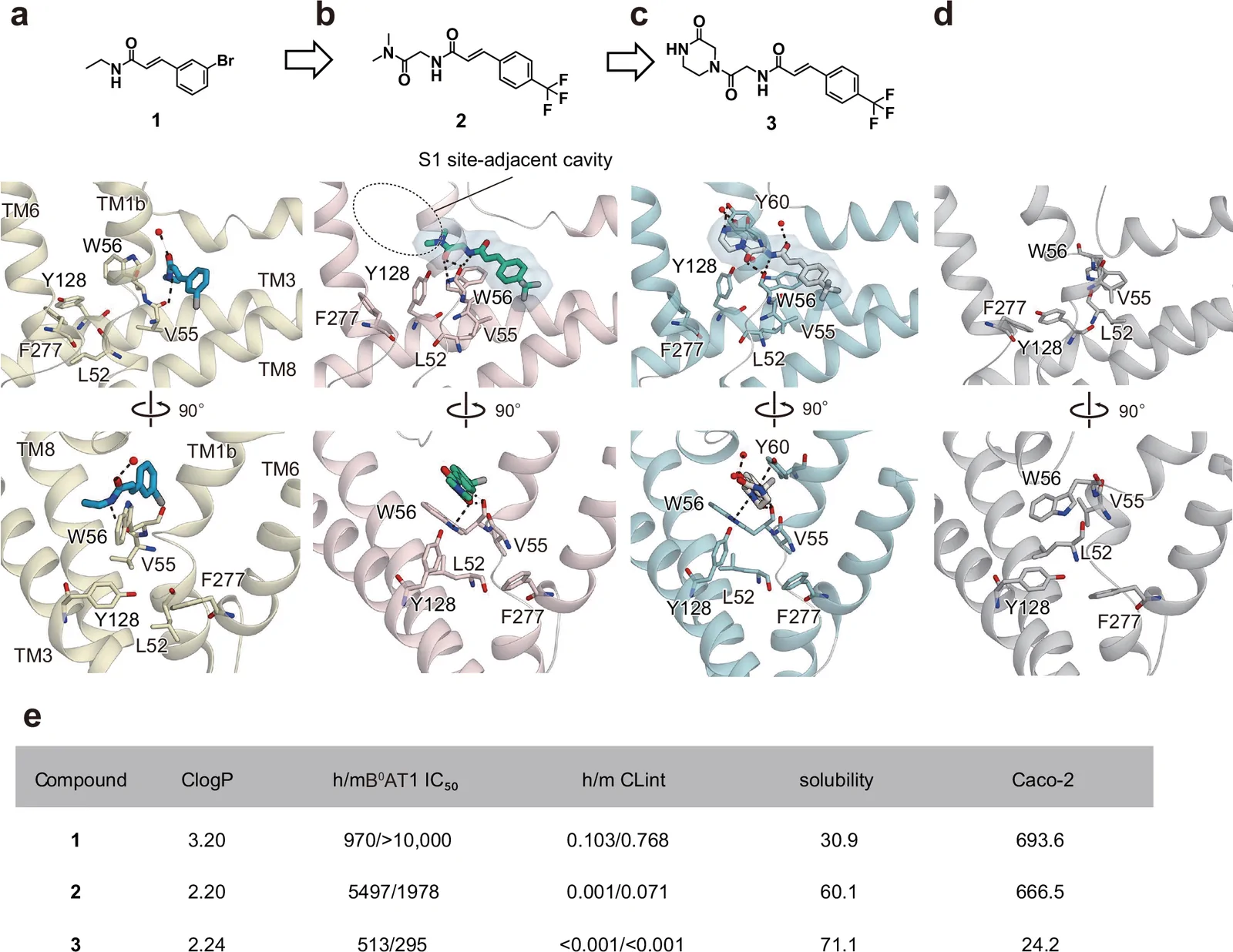
Comparison of binding sites and properties of compounds 1–3. Cryo-EM structures of B^0^AT1 bound to compound 1 (**a**), compound 2 (**b**), and compound 3 (**c**) shown in the outward-occluded conformation. Two orthogonal views (rotated by 90°) are shown for each complex. Compounds 2 and 3, in contrast to compound 1, extend toward the S1 site-adjacent cavity within the translocation pathway and form additional interactions with residues such as W56. **d** The same binding site in the outward-facing apo structure. **e** Comparison of physicochemical and pharmacological properties of compounds 1–3, including inhibitory activities against human and mouse B^0^AT1 (IC_50_, nM), along with metabolic stability (CLint, mL/min/g protein), membrane permeability (Caco-2, Papp A to B, ×10^−7^ cm/s), lipophilicity (ClogP), and solubility (JP2, µg/mL). Papp A to B represents the apparent permeability coefficient from the apical to the basolateral side.

Because of the low inhibitory activity of compound 1 against mouse B^0^AT1, in vivo efficacy testing in mice is not feasible. Therefore, we optimized compound 1 to improve its inhibitory potency against mouse B^0^AT1 (Fig. 2a–d). Our strategy focused on modifying interactions with two residues near the compound 1 binding site: Ile136 and Val317 in human B^0^AT1, which correspond to Val136 and Ala317 in mB^0^AT1 (Supplementary Fig. 4a, b). We hypothesized that these species-specific amino acid differences underlie the poor inhibition of mouse B^0^AT1 by compound 1. Ile136 is located near the ethylamine terminus of compound 1, and Val317 is near the cinnamic acid terminus. We reasoned that alkyl interactions might be insufficient for high-affinity binding to mouse B^0^AT1. To enhance binding and improve metabolic stability, we introduced chemical modifications, including a para-CF₃ group on the cinnamic acid ring and replacement of the ethylamine with a dimethylaminoglycine group. The resulting compound 2 showed improved metabolic stability and reduced lipophilicity, but at the expense of potency against human B^0^AT1 (IC_50_: 5497 nM for hB^0^AT1, 1978 nM for mB^0^AT1; Fig. 2e). Cryo-EM structures revealed that compound 2 binds to the same extracellular allosteric site as compound 1, with Na⁺ absent from the canonical sodium-binding sites (Fig. 2a, b and Supplementary Fig. 5a–c). Unexpectedly, compound 2 induces local rearrangements in the surrounding amino acids. Specifically, compound 2 forms hydrogen bonds with the main chain carbonyl oxygen of Trp56, which shifts toward TM3 and forms π interactions with the compound’s amide group (Fig. 2b). As a result, the Leu52 side chain reverted to its apo-like conformation, occluding the canonical substrate-binding site (See Supplementary Fig. 9 for a structural comparison of the occluded and non-occluded conformations).

Because compound 2 successfully reduced the species difference and gained inhibitory activity against mouse B^0^AT1, we further improved its potency by engaging the flexible regions of TM1 and TM6 and occupying the S1 site adjacent cavity within the translocation pathway. This cavity did not contribute to binding in compound 2, so we extended the substituent toward this space to strengthen interactions and enhance inhibitory activity. To this end, we replaced the dimethylamino group of compound 2 with a piperazinone moiety. The resulting compound 3 exhibited improved potency against both human and mouse B^0^AT1, with minimal species difference (IC_50_: 513 nM for hB^0^AT1, 295 nM for mB^0^AT1; Fig. 2e). The cryo-EM structure showed that the piperazinone group extends closer to the S1 site adjacent cavity while maintaining a binding mode similar to that of compound 2 (Fig. 2c and Supplementary Fig. 5d–f).

Cell membrane-binding assays using human B^0^AT1-ACE2 co-expressing cells revealed that the binding affinities (*K*_d_) of compounds 1–3 were 472, 140, and 47.0 nM, respectively (Supplementary Fig. 6). Thermal stability assays on human B^0^AT1 showed increases of 1.1, 2.7, and 6.4 °C, respectively, relative to the control (44.5 °C) (Supplementary Fig. 7). Notably, binding affinity (*K*_d_) does not exactly correlate with their inhibitory potency (IC_50_); compound 2 exhibited lower inhibitory potency than compound 1. These observations suggest that the discrepancy between affinity and inhibitory potency among compounds 1–3 may arise from multiple factors, including differences in ligand-protein interactions and the extent of conformational adjustments induced upon binding. Compound 1 is associated with more pronounced local rearrangements-such as the Leu52 shift-which may contribute to its stronger inhibitory effect (lower IC_50_) despite its weaker affinity. For compound 3, the improved IC_50_, *K*_d_, and thermal stabilization values (*T*_m_) are consistent with strengthened interactions within the expanded S1 site adjacent cavity. While interactions with Trp56 remain similar to those observed for compound 2, compound 3 additionally engages in hydrogen bonding with Tyr60 and structured water molecules, as well as hydrophobic contacts mediated by the piperazinone ring projecting into this cavity. These features likely promote a more energetically favorable binding mode. However, because the binding assay was performed using membrane fractions lacking an Na⁺ gradient, potential effects of altered transporter energetics cannot be fully excluded.

**Fig. 3:**
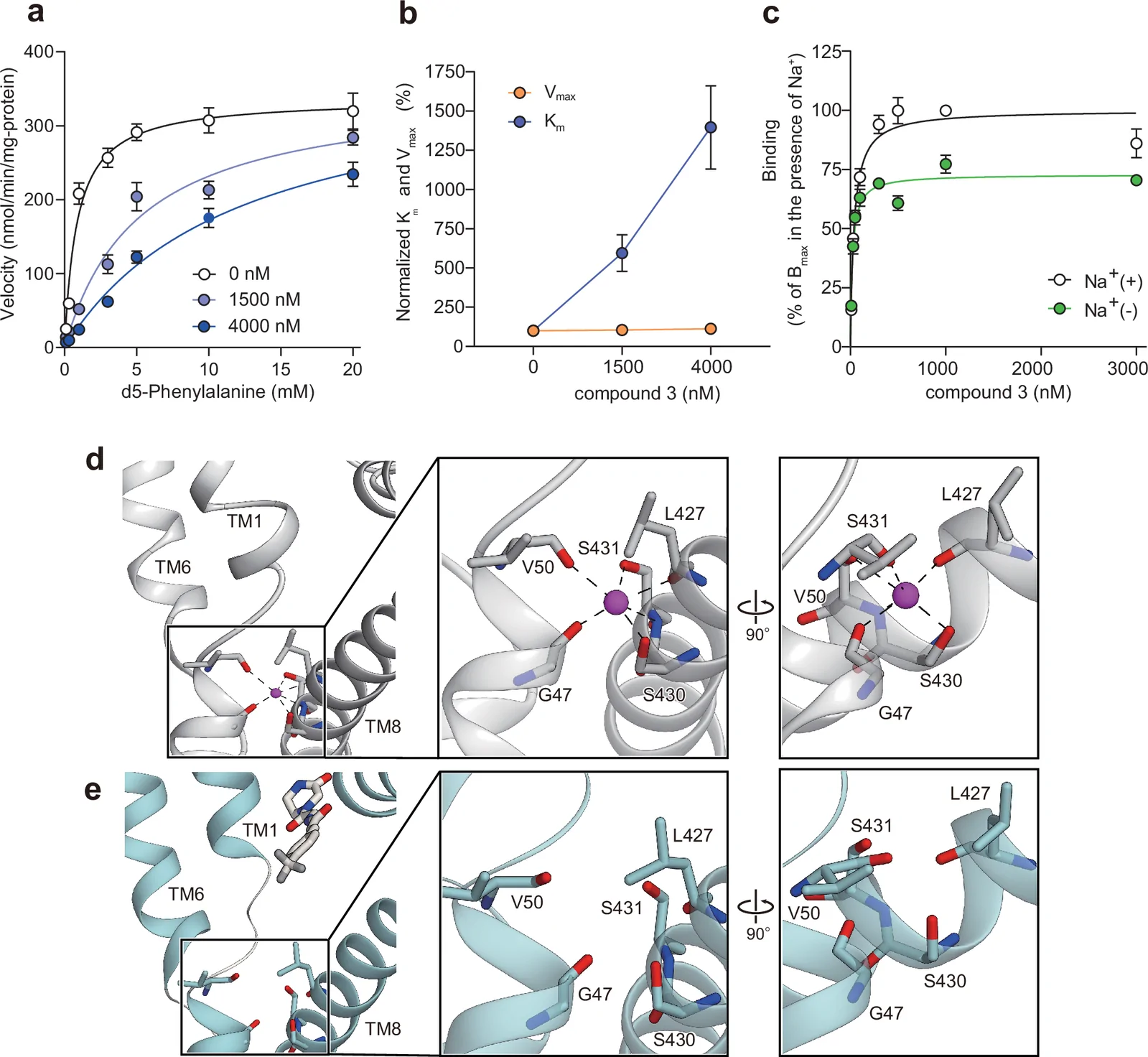
Competitive binding of B^0^AT1 inhibitors. **a** Competitive inhibition of compound 3 in d5-phenylalanine uptake by hB^0^AT1. Each point represents mean ± SEM (*n* = 3, biological replicates). **b** Kinetics parameters in d5-phenylalanine uptake by hB^0^AT1 in the presence of compound 3. Each point represents mean ± SEM (*n* = 3, biological replicates). **c** Concentration-dependent binding of compound 3 in the presence and absence of Na^+^. Specific binding was normalized to the *B*_max_ in the presence of Na^+^. Each point represents mean ± SEM (*n* = 3, technical replicates). **d**) Na2 site in the apo structure. **e** Complex structure of compound 3 with B^0^AT1 around the Na2 site.

Finally, to further investigate the inhibition mechanism of compound 3, we performed kinetic assays using stable isotope-labeled d5-Phe uptake in cells expressing B^0^AT1-Collectrin. Compound 3 increased the *K*_m_ for d5-Phe without affecting *V*_max_, indicating competitive inhibition (Fig. 3a, b and Supplementary Data 1). In addition, binding assays in the presence and absence of Na^+^ revealed a higher *B*_max_ value under Na⁺-rich conditions indicating that compound 3 preferentially binds to the Na⁺-bound conformation of B^0^AT1 (Fig. 3c and Supplementary Data 2). Structural comparison of the Na2 site in the apo and inhibitor-bound conformations further showed that compound 3 binds near the Na2 site without directly coordinating the ion (Fig. 3d, e).

### In vivo pharmacological effects in Pah^*enu2*^ mice

**Fig. 4:**
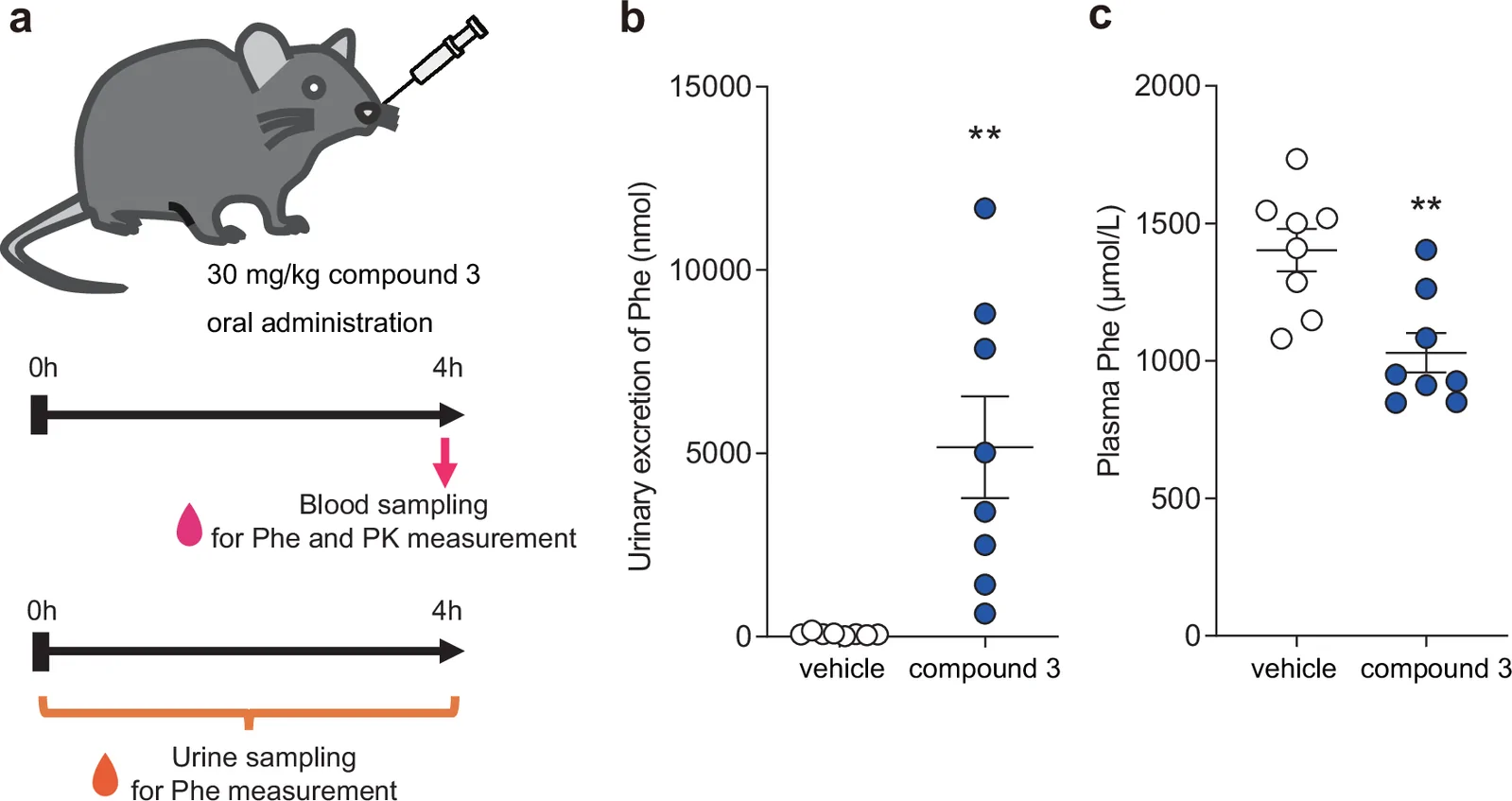
In vivo pharmacological effects of compound 3 in male *Pah*^*enu2*^ mice. **a** Schematic of the in vivo study design. Male *Pah*^*enu2*^ mice were orally administered compound 3 (30 mg/kg) and blood and urine samples were collected after 4 h post-dose. **b** Urinary excretion of phenylalanine (Phe) measured 4 h after compound 3 administration. Mice were placed in metabolic cages immediately after dosing and urine was collected over 4 h. **c** Plasma Phe concentrations measured 4 h after oral administration of compound 3. Phe levels were quantitated by LC-MS/MS. Data are shown as mean ± SEM (*n* = 8 mice per group). Each dot represents an individual mouse. **p < 0.01 compared to vehicle group (Student’s *t*-test).

Because of its favorable metabolic stability and solubility, we evaluated the in vivo effect of compound 3 on Phe levels in male *Pah*^*enu2*^ mice (Fig. 4a)^26^. These mice carry a homozygous F263S mutation in the *Pah* gene, resulting in a markedly reduction in PAH enzymatic activity and elevated plasma Phe levels, and detectable urinary excretion. They serve as a well-established model of PKU.

Following oral administration of compound 3 (30 mg/kg), the compound was detectable in plasma at 4 and 8 h post-dose (Supplementary Data 3). Although compound 3 shows low membrane permeability (Caco-2, Papp A to B, 24.2 × 10^−7^ cm/s; Fig. 2e), where Papp A to B represents the apparent permeability coefficient from the apical to the basolateral side, its detectable plasma levels are likely attributable to its relatively low molecular weight and high thermodynamic solubility. In 12-week-old male *Pah*^*enu2*^ mice, compound 3 treatment significantly increased urinary Phe excretion and concomitantly reduced plasma Phe concentrations 4 h after dosing (Fig. 4b, c). These findings are consistent with the proposed mechanism of B^0^AT1 inhibition, whereby reduced systemic Phe levels may reflect effects on intestinal absorption and/or renal handling. However, the present experiments do not allow discrimination between these potentially independent processes.

### Structural and functional analyses of the inhibition mechanism

**Fig. 5:**
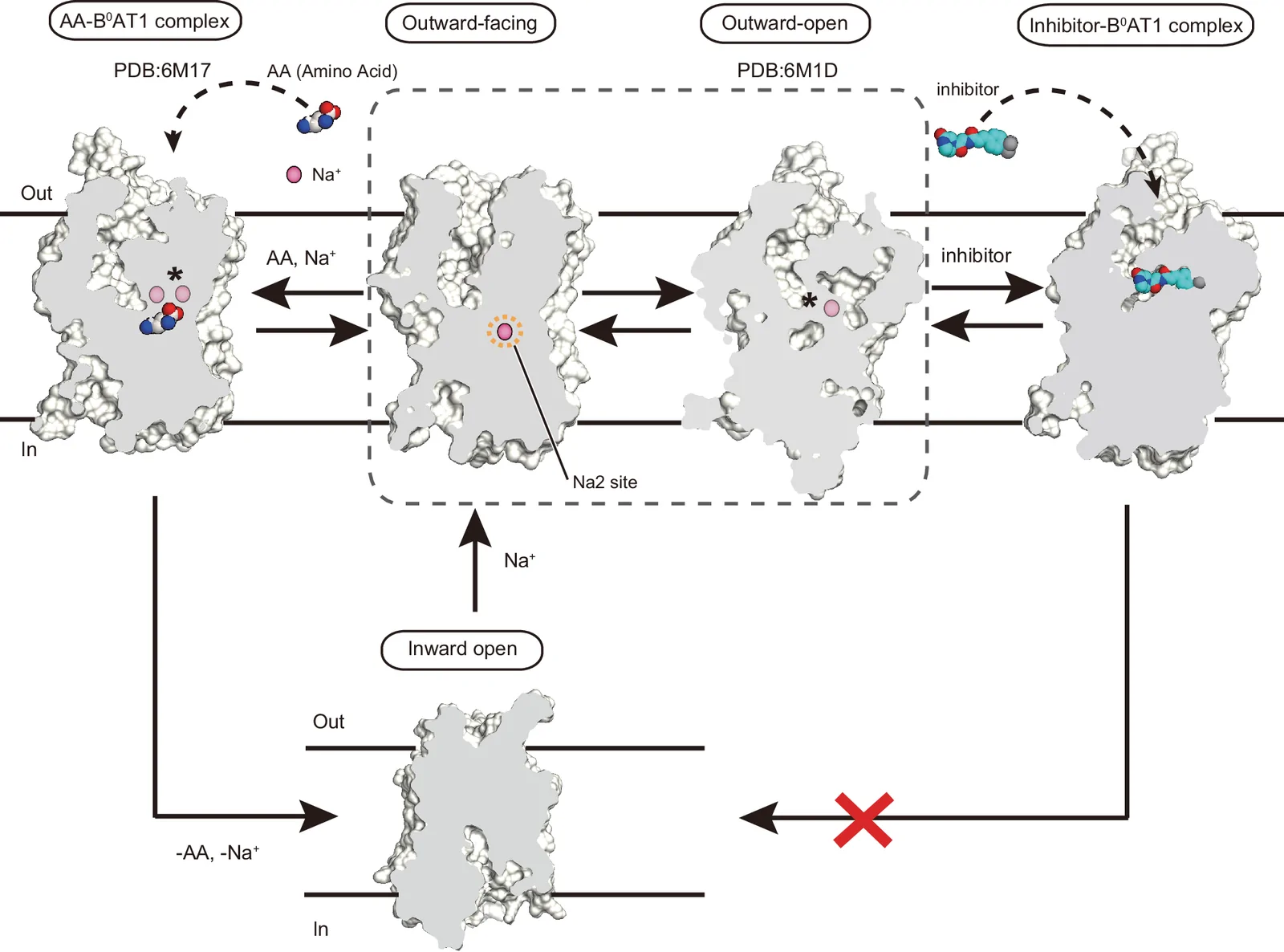
Proposed mechanism of B^0^AT1 inhibition based on Na⁺-coupled conformational transitions. Schematic representation of the transport cycle of B^0^AT1, illustrating transitions between outward-facing, outward-open, and inward-open conformations and the coupling of substrate (AA) and Na⁺ binding and release. In the absence of inhibitor, B^0^AT1 undergoes conformational changes to transport amino acids across the membrane. Inhibitors bind to the outward-open conformation and stabilize an outward-occluded-like state, thereby preventing the transition to the inward-open conformation and blocking the transport cycle. The asterisk indicates uncertainty in Na⁺ occupancy, including the number of bound ions and their specific binding sites. The number of Na⁺ ions shown is inferred from adjacent states in the transport cycle. The outward-open conformation was modeled based on PDB ID: 6M1D, and the AA-B^0^AT1 complex was generated from PDB ID: 6M17. For clarity, the surface representation is sectioned to visualize the continuous pathway from the extracellular side to the central cavity.

We next examined the inhibition mechanism of the developed B^0^AT1 inhibitors by comparing the present cryo-EM structures with related transporter conformations. B^0^AT1 functions through an alternating-access mechanism, in which Na⁺ and substrate bind simultaneously to the outward-facing state, triggering transitions through occluded intermediates to the inward-open conformation (Fig. 5).

In structural analysis, Na⁺ density was observed at the Na2 site in the outward-facing apo structure but not in the inward-open state (Supplementary Fig. 1a, b), suggesting its role in stabilizing the outward conformation. This interpretation aligns with MD simulations of LeuT showing that Na⁺ binding at the Na2 site promotes outward-facing conformations and substrate engagement^27,28^. Notably, Na⁺ binding can occur in the absence of substrate, consistent with intermediate outward-facing states observed under substrate-free conditions. Supporting this, adding Na⁺ enhanced inhibitor binding, and compound 3 competed with Phe in uptake assays (Fig. 3a–c and Supplementary Data 1, 2). This is consistent with the inhibitor pocket being exclusive to the outward-open state (Supplementary Fig. 8), confirming that inhibitor binding prevents substrate access. Together, these findings indicate that Na⁺ is not essential for inhibitor binding but promotes the outward-open conformation, thereby facilitating inhibitor access to the allosteric pocket.

## Discussion

Based on the structural and functional analyses above, we propose a mechanism in which B^0^AT1 inhibitors exert their effect by binding to a transient pocket that appears in the outward-substrate-binding site, thereby preventing substrate amino acids from accessing the transporter and effectively halting its activity (Fig. 5). Notably, this binding event stabilizes the outward-facing conformation and prevents the transition to the inward-facing conformation. As a result, the compound-bound transporter is unable to proceed through the transport cycle. This mechanism supports a model in which the inhibitors function by locking the transporter in an inactive, outward-facing conformation^20^. In addition, the inhibitors exhibited competitive inhibition despite binding to an allosteric site distinct from the canonical substrate-binding pocket (Fig. 3a, b). This indicates that allosteric inhibitors can functionally mimic competitive inhibition by inducing conformational occlusion of the substrate-binding site.

The discrepancy between binding affinity and inhibitory potency observed among compounds 1–3 likely arises from multiple factors that collectively shape the ligand-induced conformational landscape of B^0^AT1. Although compound 2 shows stronger binding affinity than compound 1, its inhibitory potency is lower, suggesting that affinity alone does not directly translate into functional inhibition. One possible explanation is that compound 1 induces larger conformational adjustments in the extracellular gate region-exemplified by the Leu52 shift-which may more effectively disrupt the transport cycle. In contrast, compound 3 exhibits improvements in *K*_d_, *T*_m_, and IC_50_, consistent with enhanced ligand-protein interactions achieved by occupying the S1 site adjacent cavity within the translocation pathway. These additional interactions likely stabilize a more inhibitory conformation, leading to a synergistic enhancement of potency. This interpretation aligns with the structural comparison showing that compound 3 better engages the flexible regions of TM1 and TM6 compared with compound 2. It is also important to note that our binding assays were performed using membrane fractions, in which the Na⁺ gradient required for productive transport is absent. Therefore, the energetics of ligand engagement may differ from physiological conditions, and this factor may contribute to the observed divergence between affinity and function. Nevertheless, the consistent structure-activity trend across *K*_d_, *T*_m_, and IC_50_ measurements supports the conclusion that the potency gains in compound 3 primarily result from optimized interactions within the expanded S1 site adjacent cavity and the associated conformational effects on the extracellular gate.

To better understand the functional significance of the allosteric inhibition observed in this study, we next examined the structural characteristics of the compound-binding pocket. This pocket is located at the outer gate of B^0^AT1 and is formed by transmembrane helices TM1b, TM3, TM7, and TM8-core elements of the LeuT-fold scaffold that are involved in conformational transitions. In the compound-bound structures, compound 1 disrupted the substrate pocket by flipping Leu52, whereas compounds 2 and 3 stabilized an occluded conformation lacking substrate (Supplementary Fig. 9). In transporter-targeted drug design, canonical substrate-binding sites typically serve as the primary targets. However, because B^0^AT1 transports a broad range of small amino acids, designing substrate analogs with high specificity is challenging. In contrast, the allosteric site identified here provides a promising alternative: it is structurally stable, suitable sized for small-molecule binding, and functionally essential. While the overall architecture of this site is conserved, sequence divergence at key positions may allow for selective recognition by small molecules. (Supplementary Fig. 10a). Notably, this allosteric site is also present, albeit more restricted, in the bacterial transporter LeuT (Supplementary Fig. 10b; PDB ID: 3TT1). Moreover, a recent study reported an allosteric inhibitor binding to the equivalent site in the dopamine transporter DAT^29^, reinforcing the idea that this pocket may serve as a generalizable target for drug development across structurally related SLC6-family transporters.

Together, these features highlight the allosteric pocket as a structurally and pharmacologically tractable site for the development of selective B^0^AT1 inhibitors. Compound 1 showed inhibitory activity against human B^0^AT1 but not against mouse B^0^AT1, whereas compounds 2 and 3 showed no species-specific differences. Compounds 2 and 3 likely share similar binding modes between the two species, as supported by comparable inhibitory potencies and binding affinities (Supplementary Fig. 6). In contrast, compound 1 bound only to human B^0^AT1, despite the high sequence identity (86.8%) between human and mouse orthologs. Although Val317 and Ile136-residues differing between species near the binding site (Supplementary Fig. 4)-were initially considered as potential contributors to the observed species difference, they do not fully account for the loss of compound 1 activity in mouse B^0^AT1. The structure of mouse B^0^AT1 has not been reported, but given the overall sequence similarity, the binding pocket is expected to be largely conserved. One possible explanation lies in the dynamic nature of transporters: substrate binding and transport involve large-scale conformational changes, and species-specific differences outside the binding site may influence these structural transitions and affect compound efficacy. These findings underscore the importance of considering not only static binding pockets but also conformational flexibility and remote residue contributions when designing inhibitors targeting structurally dynamic transporters like B^0^AT1.

Starting from compounds with in vitro inhibitory activity against B^0^AT1, we developed potent inhibitors that reduce plasma phenylalanine levels in a PKU mouse model. These inhibitors act via a previously identified allosteric site, offering high specificity and the potential for reduced side effects-key features for the lifelong management of PKU. Notably, we found that extracellular Na⁺ stabilizes the outward conformation of B^0^AT1 and enhances the binding of allosteric inhibitors, which is advantageous for in vivo use given the high Na⁺ concentration in plasma.

Overall, our study provides detailed structural insight into how inhibitors targeting this allosteric site modulate B^0^AT1 activity. The higher-resolution structures reveal key interactions, including water-mediated coordination and conformational changes around Leu52, that explain the potency and selectivity of compound 1. These findings provide a structural framework for the rational optimization of B^0^AT1 inhibitors.

## Methods

### Reagents and chemicals

The synthesis of compounds 2 and 3 is described in the Supplementary Information. Compound 1 was purchased from Angene International, Ltd. (Nanjing, China). Phenylalanine was purchased from Sigma-Aldrich (St. Louis, MO). D5-phenylalanine was purchased from C/D/N Isotopes (Quebec, Canada).

### cDNA constructs

Wild-type- *SLC6A19* (UniProt accession: Q695T7) and *ACE2* (UniProt accession: Q9BYF1) constructs were prepared as previously described^19^. *SLC6A19* cDNA and human *ACE2* cDNA were synthesized and codon-optimized for expression in human cell lines. Both cDNAs were, respectively, subcloned into the pcDNA3.4 vector. An N-terminal FLAG tag was fused to B^0^AT1, and a Strep-tag was fused after the N-terminal signal peptide of ACE2.

### Expression and purification of wild-type B^0^AT1-ACE2

Mammalian FreeStyle 293-F cells (ThermoFisher Scientific, Waltham, USA) were grown and maintained in FreeStyle 293 Expression Medium (ThermoFisher Scientific, Warrington, USA) at 37 °C and 8% CO_2_ under humidified conditions. The cells were transiently transfected at a density of 1.8 × 10^6^ cells/mL with the plasmids and PEI Max (Polysciences). For transfection, approximately 270 μg of the B^0^AT1 plasmid and 270 μg of the ACE2 plasmid were premixed with 1890 μg of PEI Max in 60 mL medium for 15–30 min before transfection. Then, 60 mL of this mixture was added to 540 mL of the cell culture (600 mL total) and incubated at 37 °C in the presence of 8% CO_2_ for 72 h before collection. The cells were collected by centrifugation (1000 × *g*, 10 min, 4 °C) and stored at −80 °C before use. To prepare the B^0^AT1-ACE2 complex, the harvested cells were solubilized for 2 h at 4 °C in solubilization buffer (25 mM Tris-HCl [pH 8.0], 150 mM NaCl, 1% GDN [Anatrace], and protease inhibitor cocktail). After ultracentrifugation (20,000 × *g*, 45 min, 4 °C) and filtering through a syringe filter (0.45 μm pore size), the supernatant was incubated with M2 Anti-FLAG Affinity Resin (Sigma-Aldrich) equilibrated with buffer A (25 mM Tris-HCl [pH 8.0], 150 mM NaCl, 0.02% GDN) and incubated for 2 h at 4 °C. The resin was washed with 5 column volumes (CVs) of buffer A, and the protein was eluted with buffer A supplemented with 0.1 mg/mL DYKDDDDK Peptide (Fujifilm Wako, Tokyo, Japan). The eluate was mixed with Strep-Tactin Sepharose (IBA), equilibrated with buffer A, and incubated for 1 h at 4 °C. After incubation, the resin was washed with 5CV buffer A, and the protein was eluted with buffer A plus 4 mM of desthiobiotin (Sigma-Aldrich). The sample was concentrated and purified via size-exclusion (Sigma-Aldrich) chromatography on Superose 6 Increase 10/300 GL (GE Healthcare) pre-equilibrated with the same buffer. The peak fractions were pooled and concentrated to 10-12 mg/mL.

### Design of stabilized B^0^AT1

As wild-type B^0^AT1 is prone to aggregation, stabilized B^0^AT1 was designed. Introduction of N-terminal and C-terminal truncates of B^0^AT1, substitution of surface-exposed cysteine with alanine, and point mutations were tested. Sequential alignment was performed with SLC6A family transporters (dopamine transporter [SLC6A3] and SERT [SLC6A4]), whose structures have been previously reported^7,9,22^, to introduce point mutations. Stabilized B^0^AT1 (hereinafter, sB^0^AT1) was successfully obtained by combining mutations (⊿2-7, Δ621-634, C500A, C610A, E81L, L114I, S290A, F471L, Y507W, and M542L), which contributed to the stabilization or improvement of expression, as assessed by fluorescence-detected size-exclusion chromatography (FSEC). For sB^0^AT1, an enhanced green fluorescent protein sequence and 8× polyhistidine tag at the C terminus of sB^0^AT1 were retained after the tobacco etch virus (TEV) cleavage site. The aforementioned stabilizing mutations were introduced into this construct via site-directed mutagenesis. The stabilized B^0^AT1 construct was used for the structural determination of the inward-open apo state in complex with a Fab.

### Expression and purification of sB^0^AT1

Mammalian HEK293S GnTI-cells (ATCC) were grown and maintained in FreeStyle 293 Expression Medium supplemented with 2% fetal bovine serum at 37 °C and 8% CO_2_ under humidified conditions. Cells were transiently transfected at a density of 2.0 × 10^6^ cells/mL using plasmids and FectoPRO (Polyplus, Illkirch, France). Approximately 200 μg of sB^0^AT1 plasmid was premixed with 200 μL of FectoPRO reagent in 80 mL of medium for 10–20 min before transfection. The sB^0^AT1 construct was originally designed for crystallographic studies prior to the publication of ref. ^19^; therefore, HEK293S GnTI⁻ cells and DDM:CHS were employed, following the standard strategies used for membrane protein crystallography at that time. For transfection, 80 mL of the mixture was added to 0.72 L of the cell culture and incubated at 37 °C with 8% CO₂ for 72 h before harvesting. The cells were collected by centrifugation (800 × *g*, 10 min, 4 °C) and stored at −80 °C before use.

To prepare sB^0^AT1, the cells were solubilized for 2 h at 4 °C in buffer (50 mM Tris-HCl [pH 7.5], 250 mM NaCl, 20% glycerol, 1.5% [w/v] n-Dodecyl-β-D-maltoside (DDM; Sigma-Aldrich), 0.15% 3β-Hydroxy-5-cholestene 3-hemisuccinate; 5-Cholesten-3β-ol 3-hemisuccinate (CHS; Sigma-Aldrich), protease inhibitor cocktail (cOmplete, Roche, Basel, Switzerland). After ultracentrifugation (142,400 × *g*, 30 min, 4 °C), the supernatant was incubated with Affi-Gel 10 (Bio-Rad) coupled with a GFP-binding nanobody^30^ and incubated for 3 h at 4 °C. The resin was washed five times with five column volumes of wash buffer (50 mM Tris-HCl [pH 7.5], 300 mM NaCl, 20% glycerol, 20 mM imidazole [pH 7.5], 0.1% DDM [Anatrace], and 0.005% CHS) and suspended in TEV protease and incubated overnight to cleave the eGFP-His tag. The flow-through was pooled, and the cleaved eGFP-His tag was removed by passing through TALON metal affinity resin pre-equilibrated with wash buffer.

N-linked glycans were removed by adding homemade His-tagged PNGaseF to the sample after TALON metal affinity resin treatment. After removing PNGaseF by passing through a Ni-NTA superflow, the samples were concentrated and purified by size-exclusion chromatography on a HiLoad Superdex75 16/600 column (GE Healthcare) equilibrated with SEC buffer (20 mM Tris-HCl [pH 7.5], 150 mM NaCl, 0.05% DDM [GLYCON Biochemicals], and 0.005% CHS). Finally, for the cryo-EM trial, the SEC buffer was replaced with a buffer containing 20 mM Tris-HCl (pH 7.5), 150 mM NaCl, and 0.06% GDN, using a Superdex200 Increase 10/300 GL column.

### Preparation of monoclonal antibody (Fab#54)

All animal experiments conformed to the Guide for the Care and Use of Laboratory Animals of Japan and were approved by the Kyoto University Animal Experimentation Committee. sB^0^AT1 was used to prepare the monoclonal antibody (Fab#54). Construct design, expression, and purification were performed as described above. Purified sB^0^AT1 was reconstructed in liposomes composed of 10:1 egg phosphatidylcholine (Sigma-Aldrich) to adjuvant lipid A (Sigma-Aldrich) to enhance the immune response. MRL/lpr mice were immunized with three injections of liposomal sB^0^AT1 at 2-week intervals. Spleen cells were collected from immunized mice and fused with myeloma cells. To select antibodies recognizing the sB^0^AT1 structure, multistep screening, including liposome-ELISA, denaturing ELISA, and fluorescence size-exclusion chromatography, was performed. For the cryo-EM trial, a large amount of the selected monoclonal antibody against sB^0^AT1 (IgG #54) was prepared via a large-scale culture of hybridoma cells. Monoclonal antibodies were initially purified with protein G resin, eluted with Gly buffer, digested with papain immobilized on N-hydroxysuccinimide-Sepharose and incubated for 5 h at 37 °C. Protein A chromatography was used to separate the Fc fragment from the Fab. The collected Fab fragment was then purified by size-exclusion chromatography on a HiLoad Superdex200 16/600 column (GE Healthcare) equilibrated with SEC buffer (20 mM Tris-HCl [pH 7.5], 150 mM NaCl, 0.05% DDM [Anatrace], and 0.005% CHS). Finally, the SEC buffer was replaced with a buffer containing 20 mM Tris-HCl (pH 7.5), 150 mM NaCl, and 0.06% GDN by ultrafiltration (Amicon Ultra-15 30 K; Millipore). The sequence of Fab#54 was determined via standard 5’-RACE using total RNA isolated from hybridoma cells.

The sequence of the Fab#54 fragment light chain is provided below:

AQAAELDIVMTQSPSSLVVSAGEKVTMTCKSSQSLFNSRTRKNYLAWYQQKPGQSPKLLIYWASTRESGVPDRFTGSGSGTDFTLTISSVQAEDLAVYYCKQSSYLLTFGAGTKLELKRADAAPTVSIFPPSSEQLTSGGASVVCFLNNFYPKDINVKWKIDGSERQNGVLNSWTDQDSKDSTYSMSSTLTLTKDEYERHNSYTCEATHKTSTSPIVKSFNRNEC

The sequence of the Fab#54 fragment heavy chain is provided below:

GGSSRSSLEVQLQQSGPELVKPGASVKISCKASGYTFTDYYINWVKQRPGQGLEWIGWIYPRSGGTKYIEKFKGKATLTVDTSSSTAYMQLNSLTSEDSAVYFCARFYYSYNEDYYALDYWSQGTSVTVSSAKTTAPSVYPLAPVCGDTSGSSVTLGCLVKGYFPEPVTLTWNSGSLSSGVHTFPAVLQSDLYTLSSSVTVTSSTWPSQSITCNVAHPASSTKVDKKIEPRGPTIKPCPPCKCPAPNLLGGPSVFIFPPKIKDVLMISLSPIVTCVVVDVSEDDPDVQISWFVNNVEVHTAQTQTHREDYNSTLRVVSALPIQHQDWMSGKEFKC

### Electron microscopy sample preparation

The purified wild-type B^0^AT1-ACE2 protein was incubated with compounds 1, 2, and 3 (0.5 mM) and incubated for 1 h on ice. The B^0^AT1-Fab#54 antibody complex was assembled by incubating sB^0^AT1 with Fab#54 at a molar ratio of 1:1.8 overnight on ice. The mixture was subjected to size-exclusion chromatography (Superdex200 Increase 10/300 GL; GE Healthcare). Fractions containing the B^0^AT1-Fab#54 complex were pooled and concentrated to 8-12 mg/mL using Amicon Ultra-15 centrifugal filters (30 kDa MWCO; Millipore). Immediately before grid preparation, 450 µM phenylalanine was added to the B^0^AT1-Fab#54 sample.

Grids were glow-discharged in low-pressure air at 10 mA using a PIB-10 vacuum device (Mito, Japan). Samples-including compound-bound, apo, and B^0^AT1-Fab#54 complexes-were applied to freshly glow-discharged Quantifoil R1.2/1.3 Cu/Rh 300-mesh holey carbon grids (SPT Labtech, Melbourn, UK) using a Vitrobot Mark IV (Thermo Fisher Scientific), which were used based on established laboratory practice, as they provided stable ice and reproducible particle distribution in our hands, at 4 °C, with a blotting time of 4–6 s under 99% humidity. Grids were then plunge-frozen in liquid ethane.

### Electron microscopy data collection and processing

The sample grids were loaded onto a Titan Krios G3i transmission electron microscope (Thermo Fisher Scientific) operating at 300 kV, equipped with a Gatan Quantum-LS energy filter (GIF) and a Gatan K3 Summit direct electron detector in correlated double-sampling (CDS) mode. Images were acquired at a nominal magnification of 105,000×, yielding a calibrated pixel size of 0.83 Å/pixel, at the University of Tokyo, Japan.

For B^0^AT1-ACE2 samples:

Movies were dose-fractionated into 48 frames at a dose rate of 13.0–13.7 e⁻/pixel/s in CDS mode, resulting in a total exposure of 49–51 e⁻/Å².

For B^0^AT1-Fab#54 samples:

Movies were acquired in non-CDS mode with a dose rate of 7.57 e⁻/pixel/s over 48 frames, resulting in a total exposure of 52 e⁻/Å².

Data collection was automated using the image-shift method in SerialEM software, with a defocus range of −0.8 to −1.6 μm. The total number of images is listed in Table 1. For all datasets, dose-fractionated movies were subjected to beam-induced motion correction using RELION^31^, and contrast transfer function parameters were estimated using CTFFIND^32^. For the compound 1-bound dataset, 328,074 particles were initially picked from 2364 micrographs using the Topaz picking function in RELION. Particles were extracted by downsampling to a pixel size of 3.2 Å/px and subjected to several rounds of 2D and 3D classifications. The best class contained 193,206 particles, which were re-extracted with a pixel size of 1.107 Å/px and subjected to 3D refinement, yielding a 3D reconstruction of the B^0^AT1-ACE2 dimer at a global resolution of 2.82 Å. For improved resolution, we used the symmetry expansion and focused classification in RELION were employed for a single subunit of the B^0^AT1-ACE2 transmembrane region complex. The second 3D classification yielded four map classes. The best class contained 125,006 particles, which were subjected to further 3D refinement, yielding a map with a global resolution of 2.7 Å, according to the FSC = 0.143 criterion. Finally, the local resolution was estimated using RELION-4. This processing strategy is illustrated in Supplementary Fig. 11.

**Table 1:** Cryoelectron microscopy (cryo-EM) data collection, refinement, and validation statistics

	Compound 1		Compound 2		Compound 3	
	B^0^AT1-ACE2 dimer	B^0^AT1 focused	B^0^AT1-ACE2 dimer	B^0^AT1 focused	B^0^AT1-ACE2 dimer	B^0^AT1 focused
EMDB ID	EMD-62632	EMD-62627	EMD-62633	EMD-62628	EMD-62634	EMD-62629
PDB ID	9KXY	9KXT	9KXZ	9KXU	9KY0	9KXV
Data collection and processing						
Magnification	105,000		105,000		105,000	
Voltage (kV)	300		300		300	
Electron exposure (e-/Å^2^)	50		50.25		48.93	
Defocus range (μm)	−0.8 to −1.6		−0.8 to −1.6		−0.8 to −1.6	
Pixel size (Å)	0.83		0.83		0.83	
Micrographs collected	2364		2826		3870	
Initial Particles after 2D	328,074		1,568,071		804,793	
Final particles						
Symmetry imposed	C2	C1	C2	C1	C2	C1
Final particle images (no.)	193,206	125,006	217,324	125,620	494,816	104,561
Map resolution (Å) (GSFSC = 0.143)	2.82	2.7	2.8	2.87	2.68	2.71
Refinement						
Initial model used (PDB ID)	6M18		6M18		6M18	
Map-to-model resolution (Å) (FSC = 0.5)	2.8	2.66	2.8	2.77	2.59	2.6
Map sharpening B factor (Å^2^)	−71.8	−61.9	−75.5	−77.4	−76.8	−56.9
Model composition						
Non-hydrogen atoms	21,360	5019	22,010	5056	22,018	4887
Protein residues	2634	617	2678	621	2678	603
Ligands	14	3	14	3	14	2
B factors (Å^2^)						
Protein	130.7	74.0	123.0	81.5	115.0	68.6
Ligand	283.4	154.4	267.9	135.9	267.1	71.2
RMS deviations						
Bond lengths (Å)	0.016	0.015	0.015	0.014	0.015	0.015
Bond angles (°)	1.970	2.050	1.790	1.900	1.860	2.130
Validation						
MolProbity score	2.25	2.22	2.44	2.19	2.24	1.83
Clashscore	13.44	7.18	19.42	9.61	14.89	6.27
Poor rotamers (%)	1.93	3.27	2.41	2.17	1.73	1.12
Ramachandran plot						
Favored (%)	94.08	93.35	94.62	93.4	94.36	93.03
Allowed (%)	5.65	6.65	5.23	6.28	5.42	6.64
Disallowed (%)	0.27	0	0.15	0.32	0.22	0.33

	Apo		Apo-antibody	
	B^0^AT1-ACE2 dimer	B^0^AT1 focused	B0AT1-Fab#54 complex	B^0^AT1 focused
EMDB ID	EMD-62635	EMD-62630	EMD-63360	EMD-62631
PDB ID	9KY1	9KXW	9LSZ	9KXX
Data collection and processing				
Magnification	105,000		105,000	
Voltage (kV)	300		300	
Electron exposure (e-/Å^2^)	50.87		52	
Defocus range (μm)	−0.8 to −1.6		−0.8 to −1.6	
Pixel size (Å)	0.83		0.83	
Micrographs collected	3087		3924	
Initial Particles after 2D	860,561		1,320,024	
Final particles				
Symmetry imposed	C2	C1	C1	C1
Final particle images (no.)	507,394	175,435	407,749	125,620
Map resolution (Å) (GSFSC = 0.143)	2.8	2.66	3.11	2.77
Refinement				
Initial model used (PDB ID)	6M18		de novo	
Map-to-model resolution (Å) (FSC = 0.5)	2.8	2.56	3.02	2.9
Map sharpening *B* factor (Å^2^)	−73.4	−55.5	−95.4	−80.4
Model composition				
Non-hydrogen atoms	22,062	4002	6458	4284
Protein residues	2684	616	1610	530
Ligands	14	2	1	1
*B* factors (Å^2^)				
Protein	161.2	74.4	125.5	95.1
Ligand	283.5	67.0	205.5	30.3
RMS deviations				
Bond lengths (Å)	0.015	0.015	0.015	0.014
Bond angles (°)	1.660	1.790	1.876	1.600
Validation				
MolProbity score	2.34	2.41	2.27	2.54
Clashscore	16.56	9.72	4.52	11.08
Poor rotamers (%)	2.15	4.38	5.08	3.84
Ramachandran plot				
Favored (%)	94.63	93.83	91.43	90.38
Allowed (%)	5.15	5.01	8.45	9.05
Disallowed (%)	0.22	0.16	0.12	0.57

The data of compound 2-3-bound or apo B^0^AT1-ACE2 (Supplementary Figs. 12–14) were collected and analyzed in the same manner. For the B^0^AT1-Fab#54 (Supplementary Fig. 15), 1,320,024 particles were initially selected from 3,924 micrographs using the topaz-picking function in RELION. Particles were extracted by downsampling to a pixel size of 3.2 Å/px and subjected to several rounds of 2D and 3D classifications. The best classes contained 407,749 particles, which were re-extracted with a pixel size of 0.83 Å/px subjected to further 3D refinement, yielding a map of the B^0^AT1-Fab#54 complex with a global resolution of 3.11 Å. Subsequent micelle subtraction and nonaligned 3D classification using a mask (without micelles) resulted in three map classes. The best class contained 125,620 particles and was further refined and postprocessed, yielding a map with a global resolution of 2.77 Å, according to the FSC = 0.143 criterion. Finally, the local resolution was estimated using RELION-4. This processing strategy is illustrated in Supplementary Fig. 15.

### Model building and validation

For B^0^AT1-ACE2 structures of apo and compound-bound forms, the Protein Data Bank (PDB) model PDB ID: 6M18 was the starting model. The model of the apo-inward conformation of B^0^AT1-Fab#54 was manually constructed de novo using COOT^33^, guided by the inward-facing structure of SERT (PDB ID: 6DZZ) and an Alphafold2^34^ -predicted model of the Fab fragment. The density map corresponding to the phenylalanine added to the B^0^AT1-Fab#54 sample was not visible and therefore not modeled.

After manual adjustment, models were structurally refined using the Servalcat^35^ pipeline in REFMAC5^36^ and manual real-space refinement in COOT. The 3D reconstruction and model refinement statistics are summarized in Table 1. All structural models in this study were built based on B^0^AT1-focused maps (Supplementary Figs. 16, 17). All molecular graphics were prepared using CueMol (http://www.cuemol.org), PyMOL (http://www.pymol.org/pymol), and UCSF Chimera^37^.

### Cell lines

The Jump-In TI platform cells (hereafter referred to as JTREx-CHO cells*) were used to generate a cell line expressing human SLC6A19 (hSLC6A19) and human Collectrin via R4 integrase-mediated DNA integration at a defined target site in accordance with the Jump-InTM TITM Gateway^®^ Targeted Integration System user guide (www.thermofisher.com)*. JTREx-CHO host cells were co-transfected with pJTI R4 Int and the tetracycline-inducible expression vector pJTR-hB^0^AT1, which carry the hSLC6A19-T2A-hCollectrin synthetic construct (synthesized by GenWiz), using Lipofectamine3000 (ThermoFisher Scientific). Successful integration of the expression vectors at the R4 attP site conferred neomycin resistance to JTREx-CHO cells by cointegrating the EF1a promoter upstream of the promoterless neomycin resistance gene. Retargeted cells were selected for G418 sulfate resistance, cloned, and used for measurements. Mouse SLC6A19 (mSLC6A19)-Collectrin co-expressing cells were similarly established using the pJTR-mB^0^AT1 vector carrying the mSLC6A19-T2A-mCollectrin synthetic construct (synthesized by GenWiz) instead of the pJTR-hB^0^AT1.

### d5-Phenylalanine uptake in B^0^AT1-Collectrin co-expressing cells

JTREx-CHO hB^0^AT1 cells were maintained in Dulbecco’s modified Eagle medium/F-12 (ThermoFisher Scientific) supplemented with 10% fetal bovine serum (HyClone), 0.1 mg/mL hygromycin B, and 0.5 mg/mL G418 at 37 °C in a humidified 5% CO₂ atmosphere. Cells were seeded at 2.0 × 10^5^ cells/well in 24-well plates with or without 2.5 µM doxycycline and cultured for 24 h. The medium was removed, the cells were washed twice and pre-incubated with extracellular fluid buffer (100 mM NaCl, 2 mM KCl, 1.8 mM CaCl_2_, 1 mM MgSO_4_, and 5 mM HEPES; pH 7.4) at 37 °C for 60 min with or without inhibitor. After preincubation, uptake was initiated by adding d5-phenylalanine solution to a final concentration of 200 µM. Uptake was completed by removing the uptake buffer and washing thrice with ice-cold buffer, followed by solubilization in 1 N NaOH. Cell lysates were deproteinized by adding acetonitrile containing d8-phenylalanine as an internal standard. The d5-phenylalanine concentration was quantified by liquid chromatography-tandem mass spectrometry (LC-MS/MS) using the internal standard method. Cellular protein content was determined using a bicinchoninic acid protein assay (ThermoFisher Scientific). d5-Phenylalanine uptake was expressed as the cell-to-medium ratio (μL/mg-protein; the ratio of cellular concentration [pmol/mg-protein] to medium concentration [pmol/µL]).

For inhibition analysis, the cell-to-medium ratio of cells incubated without doxycycline was used as the background. Specific d5-phenylalanine uptake was calculated by subtracting this background from the total cell-to-medium ratio and normalizing to the uptake achieved without the inhibitor. Kinetic parameters were calculated by nonlinear regression using GraphPad Prism 8.4.3 (San Diego, CA, USA; Supplementary Fig. 18).

For pharmacological evaluation of amino acid transport activity, d5-phenylalanine uptake assays were performed using B^0^AT1 co-expressed with Collectrin, a physiological partner of B^0^AT1 in renal transport studies. Other biochemical and binding assays were conducted using the indicated expression systems.

### Thermostability measurement

Detergent-solubilized proteins were analyzed via fluorescence-detection size-exclusion chromatography-based thermostability assay (FSEC-TS) to monitor intrinsic tryptophan fluorescence using an ACQUITY UPLC BEH SEC 4.6 mm × 150 mm column (Waters). Purified B^0^AT1-ACE2 was mixed with the compound dissolved in DMSO (or DMSO only as control) to a final concentration of 0.1 µM B^0^AT1-ACE2, 500 µM compound (or no compound), and 0.5% DMSO and incubated at 4 °C for 60 min. The samples were then aliquoted into PCR tubes, 45 µL each, and incubated at 4, 10, 20, 25, 30, 35, 40, 42.5, 45, 47.5, 50, 52.5, 55, 60, 65, and 70 °C for 10 min in a PCR Thermal Cycler SP (Takara Bio). The samples were centrifuged again (20,000 × *g*, 60 min, 4 °C) to clear the lysate, and 3 μL of the supernatant was placed in the SEC column, pre-equilibrated with buffer containing 50 mM Tris, pH 8.0, 150 mM NaCl, and 0.02% GDN (Anatrace).

### B^0^AT1 inhibitor binding assay

B^0^AT1 inhibitor binding was measured using a membrane-based assay according to a previously reported method with minor modifications^38^. FreeStyle 293-F cells were co-transfected with hB^0^AT1 and hACE2 plasmids. The cells were collected and disrupted by sonication in a hypotonic buffer (50 mM Tris-HCl [pH 7.5], 10 mM KCl, and a protease inhibitor cocktail). Cell debris was removed by centrifugation (2000 × *g*, 5 min, 4 °C). The membrane fraction was collected by ultracentrifugation (112,000 × *g*, 30 min, 4 °C) and stored at −80 °C before use. The crude membrane (250 µg per sample) was incubated with the inhibitor in an assay buffer (100 mM NaCl and 10 mM HEPES/Tris, pH 7.4) or Na^+^-free assay buffer (100 mM choline chloride and 10 mM HEPES/Tris, pH 7.4) at 25 °C for 4 h. The reactions were terminated by filtering through a GF/C filter plate (Corning) presoaked in assay buffer containing 0.1% bovine serum albumin. The sample in the filter plate was washed thrice with assay buffer and eluted with acetonitrile:water (80:20, v/v). The solution extracted from the filter plate was diluted with formic acid:water (1:1000, v/v) containing verapamil as the internal standard. Inhibitor concentration was quantified using LC-MS/MS.

Nonspecific binding was measured using the crude membrane of non-transfected FreeStyle 293-F cells. Specific binding was calculated by subtracting nonspecific binding from the binding of hB^0^AT1-expressing cells. The equilibrium dissociation constant (*K*_d_) and maximum number of binding sites (*B*_max_) were calculated via nonlinear regression using GraphPad Prism 8.4.3.

### Quantification of B^0^AT1 inhibitors bound to the crude membrane and d5-phenylalanine in cell lysates using LC-MS/MS

The concentrations of the extract solution from the filter plate and cell lysate were quantified using a tandem mass spectrometer QTRAP6500 System (SCIEX) coupled with an ACQUITY UPLC system (Waters) using the internal standard method. Mobile phases A and B consisted of formic acid:water (1:1000, v/v) and acetonitrile, respectively. Chromatographic separation was performed on an ACQUITY UPLC BEH C18 column (2.1 mm × 100 mm, 1.7 μm; Waters) at 50 °C with the following gradient of mobile phase B: 2% (0.00–0.50 min), 2–98% (0.50–2.00 min), 98% (2.00–2.50 min), and 2% (2.51–3.00 min); the flow rate was 0.4 mL/min. Mass spectrometric detection was performed by multiple reaction monitoring in the electrospray ionization positive-ion mode controlled by Analyst 1.6.2, using m/z 255.3/102.8 for compound 1; 301.2/199.0 for compound 2; 356.2/198.9 for compound 3; 171.2/125.0 for d5-phenylalanine; and 174.2/128.0 for d8-phenylalanine.

### Preparation of compound and phenylalanine administration solution

The test compound was weighed and suspended in 0.5% carboxymethyl cellulose. L-phenylalanine (Sigma-Aldrich) was dissolved in physiological saline to prepare a 25 mg/mL solution. This solution was prepared on the day of phenylalanine administration.

### Administration of compound and collection of biospecimen

Male *Pah*^*enu2*^ mice (12 weeks old, The Jackson Laboratory Japan, Inc., Yokohama, Japan) were used as test animals. Two days before the experiment, mice were placed in metabolic cages for urine collection. After habituation, mice were randomly assigned into two groups with matched mean body weights and variances, verified by an in-house algorithm. The test compound was orally administered at a dose of 30 mg/10 mL/kg. At 4 h after administration, urine samples were collected, and blood samples were collected from the tail vein. Blood was collected in a heparinized capillary tube and centrifuged to obtain plasma.

All experiments were conducted using male mice. All animal experiments were approved by the Institutional Animal Care and Use Committee of Mitsubishi Tanabe Pharma Co., Ltd. and were conducted in accordance with institutional guidelines. We have complied with all relevant ethical regulations for animal use.

### Quantification of phenylalanine in mouse urine and plasma via LC-MS/MS

Mouse plasma and urine concentrations were quantified using a tandem mass spectrometer (Quattro Premier XE, Waters, Milford, Massachusetts, USA) coupled to an ACQUITY UPLC system (Waters, Milford, MA, USA) using the internal standard method. Samples were diluted in saline, deproteinized with acetonitrile containing d5-phenylalanine as an internal standard, and centrifuged, and the supernatant was subjected to LC-MS/MS. Mobile phases A and B were heptafluorobutyric acid:water (0.25/1000, v/v) and acetonitrile, respectively. Chromatographic separation was performed on an ACQUITY UPLC HSS C18 column (2.1 mm × 100 mm, 1.8 µm; Waters) at 50 °C, with the following gradient of mobile phase B: 2% (0.00–0.50 min), 2–98% (0.50–3.50 min), 98% (3.50–4.25 min), and 2% (4.26–5.00 min); the flow rate was 0.4 mL/min. Mass spectrometric detection was performed using multiple reaction monitoring in the electrospray ionization positive-ion mode controlled by MassLynx using m/z 166.1/120.0 for phenylalanine and 171.2/125.0 for d5-phenylalanine.

### Statistics and reproducibility

For the in vivo experiments, statistical analyses were performed using SAS 10.1 (SAS Institute, Inc.). The effect of test compounds on Phe excretion in the urine and levels in the plasma was analyzed using an unpaired, two-tailed Student’s *t*-test for comparisons between two groups. *p*-values < 0.01 were considered statistically significant. Transport and binding assays were performed in three independent experiments unless otherwise stated. Sample sizes and definitions of replicates for all experiments are provided in the corresponding figure legends. Cryo-EM experiments were performed using independently prepared protein samples. Representative data are shown where appropriate.

### Reporting summary

Further information on research design is available in the Nature Portfolio Reporting Summary linked to this article.

## Supplementary information

Transparent Peer Review file

Supplementary information

Description of Additional Supplementary Files

Supplementary Data 1

Supplementary Data 2

Supplementary Data 3

Supplementary Data 4

Reporting Summary

## Data Availability

Cryo-EM density maps were deposited in the Electron Microscopy Data Bank under the accession codes EMD-62632 (B^0^AT1-ACE2 complex with compound 1), EMD-62627 (B^0^AT1 with compound 1 focused refinement), EMD-62633 (B^0^AT1-ACE2 complex with compound 2), EMD-62628 (B^0^AT1 with compound 2 focused refinement), EMD-62634 (B^0^AT1-ACE2 complex with compound 3), EMD-62629 (B^0^AT1 with compound 3 focused refinement), EMD-62635 (apo B^0^AT1-ACE2 outward-facing), EMD-62630 (apo B^0^AT1 outward-facing focused refinement), EMD-63360 (B^0^AT1 antibody-bound inward-open conformation) and EMD-62631 (apo B^0^AT1 inward-open conformation focused refinement). The atomic coordinates have been deposited in the Protein Data Bank under IDs 9KXY (B^0^AT1-ACE2 complex with compound 1), 9KXT (B^0^AT1 with compound 1 focused refinement), 9KXZ (B^0^AT1-ACE2 complex with compound 2), 9KXU (B^0^AT1 with compound 2 focused refinement), 9KY0 (B^0^AT1-ACE2 complex with compound 3), 9KXV (B^0^AT1 with compound 3 focused refinement), 9KY1 (apo B^0^AT1-ACE2 outward-facing), 9KXW (apo-B^0^AT1 outward-facing focused refinement), 9LSZ (B^0^AT1 antibody-bound inward-open conformation) and 9KXX (apo B^0^AT1 inward-open conformation focused refinement). The supplementary data are provided in this study. Mass spectrometry-based quantitative data supporting this study have been deposited in Figshare at https://doi.org/10.6084/m9.figshare.32616318. All other data supporting the findings of this study are available from the corresponding author upon reasonable request.
