# Supplementary material for: Structure-guided development of a potent human B0AT1 inhibitor effective in a mouse model of phenylketonuria
Source: Commun Biol. 2026 Jun 25;9:1350. doi: 10.1038/s42003-026-10535-y (PMC13669362; doi:10.1038/s42003-026-10535-y)
Supplement: Supplementary file 1 [file 42003_2026_10535_MOESM1_ESM.pdf]

nature portfolio

Peer Review File

Structure-guided development of a potent human B0AT1 inhibitor effective in a mouse model of phenylketonuria

Corresponding Author: Mr Takuya Imazu

This file contains all reviewer reports in order by version, followed by all author rebuttals in order by version.

Version 0:

Reviewer comments:

Reviewer #1

(Remarks to the Author)
In their manuscript, Imazu et al. utilized cryo-EM to guide inhibitor synthesis for SLC6A19 neutral amino acid transporter, crucial for phenylalanine absorption and reuptake, with implications for treating phenylketonuria. They present in total five structures: two apo and three with different compounds, bound at an extracellular allosteric site. Based on the structure with cinromide, they synthesized two additional compounds targeting this site and characterized their binding affinities, inhibitory potency, thermal and metabolic stability, membrane permeability, lipophilicity and solubility. The pharmacological effects of the most potent inhibitor (compound 3) were additionally evaluated in vivo in mice.

Major concerns:
The cryo-EM structure determination is well performed, yielding high resolution structures in multiple conformations, however, the structural description in the main text lacks clarity and context for non-specialist readers. For example, it is never mentioned if the transporter acts as a dimer or a monomer and the authors do not discuss that they got both, dimers in all ACE2 bound structures and a monomer in Fab bound structure, which is only evident from the supplementary data. The authors should address it and also put the observed oligomeric states in respect to previously reported structures. Similarly, even though it is clear from the methods section, the authors should indicate in the main text that they performed symmetry expansion and focused classifications to get higher resolution at the inhibitor binding site and state this improved resolution in either the figure caption or the text, because this is one of the aspects that is especially important in drug design and strengthens their story. They should also clearly state in figure captions that they are displaying results from focused classifications.

In general, the main story line about cryo-EM based optimization of inhibitors reads rather well, but since it is not the first one for this transporter, the authors should be more open of what is new about this particular study and what is similar to the previous ones. For example, from lines 34 and 39 in the abstract, it sounds as if the authors identified a new “druggable allosteric site”, but this site has been already described in Xu et al., 2024. Also, they finish their manuscript stating: “Overall, this study demonstrates that targeting non-canonical, conformationally dynamic allosteric sites can yield selective and effective inhibitors, providing a promising strategy for drug discovery beyond conventional substrate-binding approaches.”(Lines 281-283). While this statement is correct, it does not reflect a novel conceptual advance and therefore weakens the impact of the closing paragraph. The manuscript would benefit from a clearer positioning of this work relative to Xu et al., 2024, explicitly stating that the allosteric site has been previously identified and characterized, and emphasizing what new structural or pharmacological insights are provided here, for example, properties of new inhibitor, higher resolution, allowing better details at the binding site, including coordination by water molecules, more detailed conformational analysis, in vivo studies.

Similarly, in lines 101-103, the authors should mention that stabilizing SLC6A19 with ACE2 is a common practice and add citations for this. In general, the authors do cite the relevant papers, but not at all places necessary to non-expert readers to get the full view.

Minor comments:
1. In Figure 2, the same chemical structures of the three compounds should not be shown twice. The density should be displayed for all compounds, not only 2 and 3, for consistency. Figure caption should be more precise and more informative.
2. Similarly, the legend of Figure 5 should be extended to describe the full sketch, not only its left side.

3. The display of Na2 site in Extended Data Fig 1a is not very convincing.

Overall, this study provides high-quality structural data and promising pharmacological characterization. Addressing the mentioned issues, particularly regarding clarity of presentation and positioning relative to prior work, would significantly strengthen the manuscript.

Reviewer #2

(Remarks to the Author)
In this manuscript, the authors report allosteric inhibitors of B0AT1 that reduce plasma phenylalanine in PKU model mice, and present cryo-EM structures showing that the binding site is exclusive to the outward-open conformation and that inhibitor binding induces an outward-occluded state independent of substrate.
It is a well-written and thorough manuscript. Addressing the following points would further strengthen it.
1. Why was the B0AT1-Collectrin co-expressing cell line used for d5-phenylalanine uptake assays rather than B0AT1-ACE2 co-expressing cells, as used for the cell-based inhibitor binding assays for compounds 1–3?
2. In line 158-159, “Cell-based binding assays using human B0AT1-ACE2 co-expressing cells… binding affinities (Kd) of compounds 1-3”, the authors mention the cell-based assays, but no detailed methods are provided for these experiments. Please include them.
3. sB0AT1 combines a lot of mutations (⊿2-7, Δ621-634, C500A, C610A, E81L, L114I, S290A, F471L, Y507W, and M542L). Has amino acid transport activity been thoroughly validated relative to wild-type? More details on this construct will benefit the audience.
4. Protocols for expressing and purifying different B0At1 constructs vary in the manuscript. Like use of Freestyle 293-F vs HEK293 GnTi- cells for expression. For purification, sB0AT1 was solubilized and purified using DDM:CHS, while wild-type B0AT1-ACE2 was purified using GDN. Please add a brief explanation for this.
5. Why Quantifoil R1.2/1.3 Cu/Rh 300-mesh holey carbon grids? In previous B0AT1 or related publications, R1.2/1.3 Au or Cu. Was there a specific reason to use Cu/Rh grids?
6. In line 191 “Although compound 3 shows low membrane permeability (Papp A to B, 24.2 × 10-7 cm/s; Fig. 2e)” and figure legends, what does “Papp A to B” stand for? Please clarify in the manuscript.

Reviewer #3

(Remarks to the Author)
While several structures of B0AT1 have been reported, this study provides important new insight. Most notable the position of the cotransported Na+ ion at the Na2 site. In addition, two conformational states are reported that are slightly different to previous structures. It is also interesting to see that inhibitors were found that have the same affinity for the mouse and human transporter.
My comments are minor and mostly for clarification.
Line 89: This statement is not quite up do date. Please see https://pmc.ncbi.nlm.nih.gov/articles/PMC11601558/
Line 125: I think you are referring to figure 2 here.
Line 196: The experiments cannot discriminate between the renal and intestinal effects of the inhibitor. Perhaps a note of caution would be in order here. The current statement seems to suggest that inhibition of the intestinal transport promotes renal excretion. However, both processes are quite independent of each other.
Line 206: The structures suggest that the Na2 site is used in B0AT1. This is a bit surprising because the Na+ concentration has an effect on the substrate Km and vice versa. Do the authors have an explanation for this?
Line 322: The design of a stabilized B0AT1 is described in the methods but it is unclear where this modified protein was used in the study. Please clarify.

Version 1:

Reviewer comments:

Reviewer #1

(Remarks to the Author)
The authors addressed all my concerns, so I can only recommend this manuscript for publication.

Reviewer #2

(Remarks to the Author)
The authors have addressed all of my critiques satisfactorily, and the manuscript has been updated to reflect their responses.

Reviewer #3

(Remarks to the Author)
The authors have addressed all comments made by this assessor.

Open Access This Peer Review File is licensed under a Creative Commons Attribution 4.0 International License, which permits use, sharing, adaptation, distribution and reproduction in any medium or format, as long as you give appropriate credit to the original author(s) and the source, provide a link to the Creative Commons license, and indicate if changes were made.
In cases where reviewers are anonymous, credit should be given to 'Anonymous Referee' and the source.
The images or other third party material in this Peer Review File are included in the article's Creative Commons license, unless indicated otherwise in a credit line to the material. If material is not included in the article's Creative Commons license and your intended use is not permitted by statutory regulation or exceeds the permitted use, you will need to obtain permission directly from the copyright holder.
To view a copy of this license, visit https://creativecommons.org/licenses/by/4.0/

Response to Reviewer #1

We thank the reviewer for the constructive comments and helpful suggestions. All points have been carefully addressed as follows.

R1-1. Comment 1

The cryo-EM structure determination is well performed, yielding high resolution structures in multiple conformations, however, the structural description in the main text lacks clarity and context for non-specialist readers. For example, it is never mentioned if the transporter acts as a dimer or a monomer and the authors do not discuss that they got both, dimers in all ACE2 bound structures and a monomer in Fab bound structure, which is only evident from the supplementary data. The authors should address it and also put the observed oligomeric states in respect to previously reported structures.

Response

We thank the reviewer for this helpful suggestion. We agree that clarifying the oligomeric state of the transporter will improve accessibility for non-specialist readers.

In previously reported structures, B^0AT1 forms a complex with ACE2, which itself forms a homodimer, resulting in a dimeric assembly of the ACE2-B^0AT1 complex. In contrast, in the Fab-bound structure determined in this study, B^0AT1 is observed as a monomer. Importantly, B^0AT1 itself does not appear to form direct dimerization interfaces, and the dimeric architecture arises from the ACE2 homodimer.

We have now clarified this point in the main text and added references to previous structural studies describing the ACE2-B^0AT1 complex.

Changes in manuscript

Text describing the oligomeric state and its relationship to previously reported structures has been added in the Results section (page 4, lines 113-120).

R1-2. Comment

Similarly, even though it is clear from the methods section, the authors should indicate in the main text that they performed symmetry expansion and focused classifications to get higher resolution at the inhibitor binding site and state this improved resolution in either the figure caption or the text.

Response

We thank the reviewer for pointing this out. We agree that highlighting this analysis in the main text strengthens the presentation of the structural results.

We have now added a description in the Results section stating that symmetry expansion and focused classification were applied to improve the local resolution at the inhibitor-binding site. The improved local resolution has also been indicated in the figure captions.

Changes in manuscript

Additional text describing symmetry expansion and focused classification has been added to the Results section (page 4, lines 113-120), and the figure captions have been updated accordingly (page 32, lines 767-768).

R1-3. Comment

The authors should be more open of what is new about this particular study and what is similar to the previous ones. For example, from lines 34 and 39 in the abstract, it sounds as if the authors identified a new “druggable allosteric site”, but this site has been already described in Xu et al., 2024. Similarly, in lines 101–103, the authors should mention that stabilizing SLC6A19 with ACE2 is a common practice and add citations.

The authors should be more open of what is new about this particular study and what is similar to the previous ones. For example, from lines 34 and 39 in the abstract, it sounds as if the authors identified a new “druggable allosteric site”, but this site has been already described in Xu et al., 2024. Similarly, in lines 101–103, the authors should mention that stabilizing SLC6A19 with ACE2 is a common practice and add citations.

Response

We thank the reviewer for this important comment. We agree that the previous identification of this allosteric binding site should be more clearly acknowledged.

In the revised manuscript, we explicitly state that the allosteric site was previously reported by Xu et al. (2024). We have revised the abstract and main text to clarify this point and to better position our study relative to prior work.

Specifically, our study provides higher-resolution structural information, detailed characterization of newly designed inhibitors, and in vivo validation of the most potent compound.

Changes in manuscript

Relevant sentences in the Abstract and Introduction have been revised to acknowledge the prior

identification of the allosteric site (page 2, lines 35-36, 37-39 and 41-42, page 3, line 83-84, page 4, lines 111-120, page 10, lines 297-307).

R1-4. Comment

Similarly, in lines 101-103, the authors should mention that stabilizing SLC6A19 with ACE2 is a common practice and add citations for this. In general, the authors do cite the relevant papers, but not at all places necessary to non-expert readers to get the full view.

Response

We have revised the text accordingly to explicitly state this point and added the relevant citations.

Changes in manuscript

To improve clarity for non-expert readers, we have revised the text to explicitly state that stabilization of B^0AT1 through complex formation with ACE2 is a commonly used strategy in previous structural studies of B^0AT1 and related transporters, and have added the appropriate citations (page 4, lines 108-109, refs. 19–21)

Minor comments

R1-5. Comment

In Figure 2, the same chemical structures of the three compounds should not be shown twice. The density should be displayed for all compounds, not only 2 and 3, for consistency. Figure caption should be more precise and more informative.

Response

We thank the reviewer for this suggestion. The figures have been revised to avoid redundant presentation of the chemical structures.

Changes in manuscript

The figure caption has been updated accordingly (page 32, lines 772-778).

R1-6. Comment

The density should be displayed for all compounds, not only 2 and 3.

Response

We thank the reviewer for the comment. Cryo-EM density is shown for all compounds: compound 1 in Extended Data Fig. 2c, and compounds 2 and 3 in Extended Data Fig. 5c and 5f.

Changes in manuscript

No changes were made to the manuscript, as the density for all compounds was already included in the original figures.

R1-7. Comment

The legend of Figure 5 should be extended to describe the full sketch.

Response

We thank the reviewer for this suggestion. The figure legend has been expanded to describe all elements of the schematic representation.

Changes in manuscript

The figure legend has been expanded to describe all elements of the schematic representation (page 33, lines 801-813).

R1-8. Comment

The display of Na2 site in Extended Data Fig 1a is not very convincing.

Response

We thank the reviewer for this suggestion. We have revised the figure to improve the visualization of the Na2 density and its coordination environment.

Changes in manuscript

We have revised the Results section to clarify the description and the figure to improve the visualization of the Na2 density and its coordination environment (page 4-5, lines 124-125, Extended Data Fig 1a).

Response to Reviewer #2

We thank the reviewer for the constructive comments and helpful suggestions. All points have been carefully addressed as follows.

R2-1. Comment

Why was the B^0AT1-Collectrin co-expressing cell line used for d5-phenylalanine uptake assays rather than B^0AT1-ACE2 co-expressing cells, as used for the cell-based inhibitor binding assays for compounds 1–3?

Response

We thank the reviewer for this important question. For pharmacological evaluation of B^0AT1 transport function, we employed a co-expression system with Collectrin, which is the physiological partner of B^0AT1 in the human kidney. We considered that the B^0AT1-Collectrin system more closely reflects in vivo renal transport function and allows robust assessment of intrinsic B^0AT1 activity. Consistent with this view, Collectrin co-expression has been widely used in previous studies to evaluate renal amino acid transport by B^0AT1 (e.g. refs. 21 and 23), and we therefore adopted this system for the d5-phenylalanine uptake assays.

Importantly, previous reports have shown that wild-type B^0AT1 exhibits comparable transport activity when co-expressed with either Collectrin or ACE2. For example, co-expression with either partner in Xenopus oocytes resulted in similar increases in leucine uptake (Gastroenterology 2009; 136: 872–882). Thus, the use of Collectrin rather than ACE2 is not expected to bias the interpretation of the transport assay results.

Moreover, Collectrin lacks the enzymatic activity associated with ACE2, allowing more direct assessment of amino acid transport. We have therefore clarified in the manuscript that pharmacological transport assays were conducted using B^0AT1 co-expressed with Collectrin.

Changes in manuscript

An additional description has been included in the Online Methods section (page18 lines 533-536).

R2-2. Comment

In line 158-159, "Cell-based binding assays using human B^0AT1-ACE2 co-expressing cells… binding affinities (Kd) of compounds 1-3", the authors mention the cell-based assays, but no detailed methods are provided for these experiments. Please include them.

Response:

We thank the reviewer for this helpful comment. The experimental procedure for the binding assay is described in the Methods section ("B^0AT1 inhibitor binding assay"). However, we agree that this was not sufficiently clear in the main text.

Changes in manuscript

We have revised the manuscript to clarify that the assay is a cell membrane-binding assay rather than a cell-based binding assay (page6, line 174, page18, line 551).

R2-3. Comment

sB^0AT1 combines a lot of mutations (⊿2-7, Δ621-634, C500A, C610A, E81L, L114I, S290A, F471L, Y507W, and M542L). Has amino acid transport activity been thoroughly validated relative to wild-type? More details on this construct will benefit the audience.

Response

We thank the reviewer for this important point. As the purpose of sB^0AT1 was structural stabilization, functional transport activity was not assessed. The stabilized B^0AT1 (sB^0AT1) construct was engineered specifically for structural studies and was not intended for functional characterization. However, our purification experiments confirmed that sB^0AT1 forms a stable complex with ACE2. All functional transport assays reported in this study were performed using wild-type B^0AT1 constructs.

Changes in manuscript

An additional description has been included in the Results section (page 4, lines 111-113).

R2-4. Comment

Protocols for expressing and purifying different B^0At1 constructs vary in the manuscript. Like use of Freestyle 293-F vs HEK293 GnTi- cells for expression. For purification, sB^0AT1 was solubilized and purified using DDM:CHS, while wild-type B^0AT1-ACE2 was purified using GDN. Please add a brief explanation for this.

Response

We thank the reviewer for this suggestion. The sB^0AT1 construct was designed and evaluated for crystallographic studies prior to the publication of reference 19. Therefore, it was expressed in HEK293 $GnTI^{-}$ cells and solubilized and purified using DDM:CHS, following the strategies

commonly used for membrane protein crystallography at that time.

In contrast, the wild-type B^0AT1-ACE2 complex was expressed and purified based on the protocol reported in reference 19, which was optimized for cryo-EM analysis. Therefore, Freestyle 293 F cells were used for expression and GDN was employed for purification to improve particle stability and homogeneity.

Changes in manuscript

An additional description has been included in the Online Methods section (page 13, lines 374-377).

R2-5. Comment

Why Quantifoil R1.2/1.3 Cu/Rh 300-mesh holey carbon grids? In previous B^0AT1 or related publications, R1.2/1.3 Au or Cu. Was there a specific reason to use Cu/Rh grids?

Response

We thank the reviewer for this comment. The choice of Quantifoil R1.2/1.3 Cu/Rh 300-mesh grids was not based on a specific experimental requirement, but rather on established laboratory practice. These grids have been routinely used in our laboratory and in previous structural studies of related membrane proteins, where they provided stable ice and reproducible particle distribution (e.g., ref. 41). In the present study, they yielded high-quality cryo-EM data, and no grid-related limitations were observed.

Changes in manuscript

A brief explanation of the grid choice has been added to the Methods section (page 15, lines 442-443).

R2-6. Comment

In line 191 "Although compound 3 shows low membrane permeability (Papp A to B, 24.2 × 10-7 cm/s; Fig. 2e)" and figure legends, what does "Papp A to B" stand for? Please clarify in the manuscript.

Response

We thank the reviewer for this suggestion. We have now clarified the definition of "Papp A to B" in the Results section.

Changes in manuscript

Additional descriptions have been included in the Results section (page 7, lines 207-208) and the

figure caption (page 32, lines 780-781).

Response to Reviewer #3

We thank the reviewer for the constructive comments and helpful suggestions. All points have been carefully addressed as follows.

R3-1. Comment

Line 89: This statement is not quite up do date. Please see

https://pmc.ncbi.nlm.nih.gov/articles/PMC11601558/

Response

We thank the reviewer for this helpful suggestion. We have updated the relevant statement in the Introduction and included the suggested reference to ensure that the description reflects the most recent findings.

Changes in manuscript

A revised description including the suggested reference has been added in the Introduction (page 3, lines 90–93).

R3-2. Comment

Line 125: I think you are referring to figure 2 here.

Response

We thank the reviewer for pointing this out. The figure reference was incorrect in the original manuscript and has been corrected to Fig. 1b.

Changes in manuscript
The figure reference has been corrected in the Results section (page 5, line 141).

R3-3. Comment

Line 196: The experiments cannot discriminate between the renal and intestinal effects of the inhibitor. Perhaps a note of caution would be in order here. The current statement seems to suggest that inhibition of the intestinal transport promotes renal excretion. However, both processes are quite independent of each other.

Response

We thank the reviewer for this helpful suggestion. We have revised the Results section to clarify that the present experiments do not allow discrimination between intestinal and renal effects of the inhibitor, and that these potentially independent processes are interpreted with appropriate caution.

Changes in manuscript

A clarified description has been included in the Results section (page 7, lines 213-216) to explicitly note that the experiments do not distinguish between intestinal and renal contributions to the observed reduction in systemic phenylalanine levels.

R3-4. Comment

Line 206: The structures suggest that the Na2 site is used in B^0AT1. This is a bit surprising because the Na+ concentration has an effect on the substrate Km and vice versa. Do the authors have an explanation for this?

Response

We thank the reviewer for this insightful comment. SLC6 transporters, including B^0AT1, couple substrate transport to the Na^+ gradient, but Na^+ binding and substrate binding are not necessarily strictly simultaneous events.

The presence of Na^+ density at the Na2 site in the apo structure likely is likely to reflect stabilization of the outward-facing conformation rather than indicating a fully substrate-bound transport state. In related SLC6 transporters, Na^+ binding is known to precede and facilitate substrate binding and can stabilize specific conformational states of the transporter.

We have added a sentence in the manuscript to clarify that the observed Na^+ density at the Na2 site is consistent with a Na^+-bound, substrate-free intermediate state and does not imply simultaneous substrate occupancy.

Changes in manuscript

An additional description has been included in the Results section (page 8, lines 227-228).

R3-5. Comment

Line 322: The design of a stabilized B^0AT1 is described in the methods but it is unclear where this modified protein was used in the study. Please clarify.

Response

We thank the reviewer for this suggestion. We have now added a description to clarify where and how the stabilized B^0AT1 construct was used in this study.

Changes in manuscript

Additional descriptions have been included in the Online methods section (page 12, lines 366-367).
