# Supplementary material for: Structure-guided development of a potent human B0AT1 inhibitor effective in a mouse model of phenylketonuria
Source: Commun Biol. 2026 Jun 25;9:1350. doi: 10.1038/s42003-026-10535-y (PMC13669362; doi:10.1038/s42003-026-10535-y)

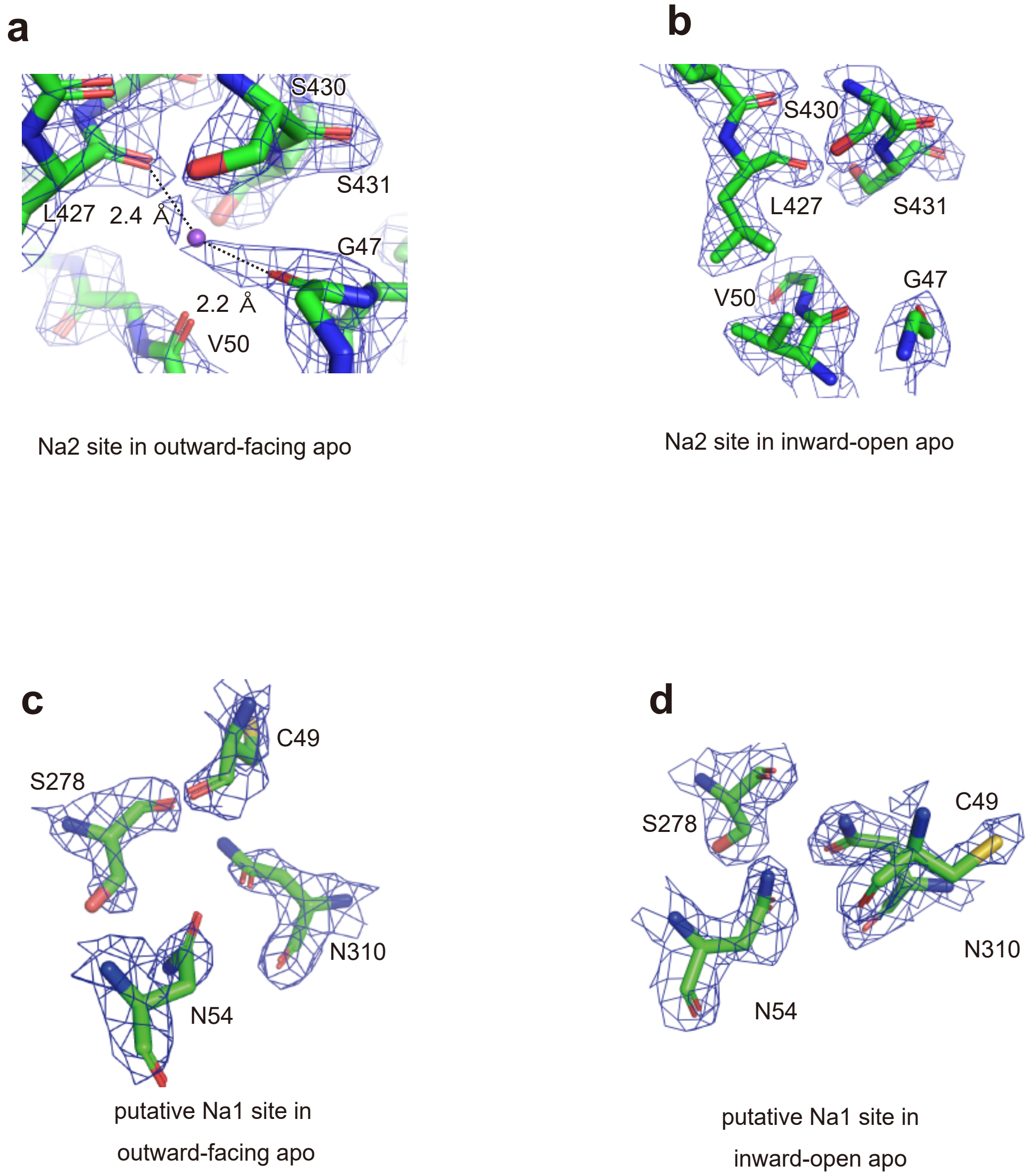

Supplementary Figure 1 Cryo-EM density maps of apo Na2 site, and apo putative Na1 site in B^0AT1
a) Na2 site of the outward-facing apo B^0AT1, with five surrounding atoms coordinated to Na^+. b) Inward-open form of apo B^0AT1. c, d) The putative Na1 sites of the the outward-facing and inward open apo B^0AT1 show no density map corresponding to Na^+. Contour levels of cryo-EM density are 2.8σ (a), and 2.0σ (b-d).

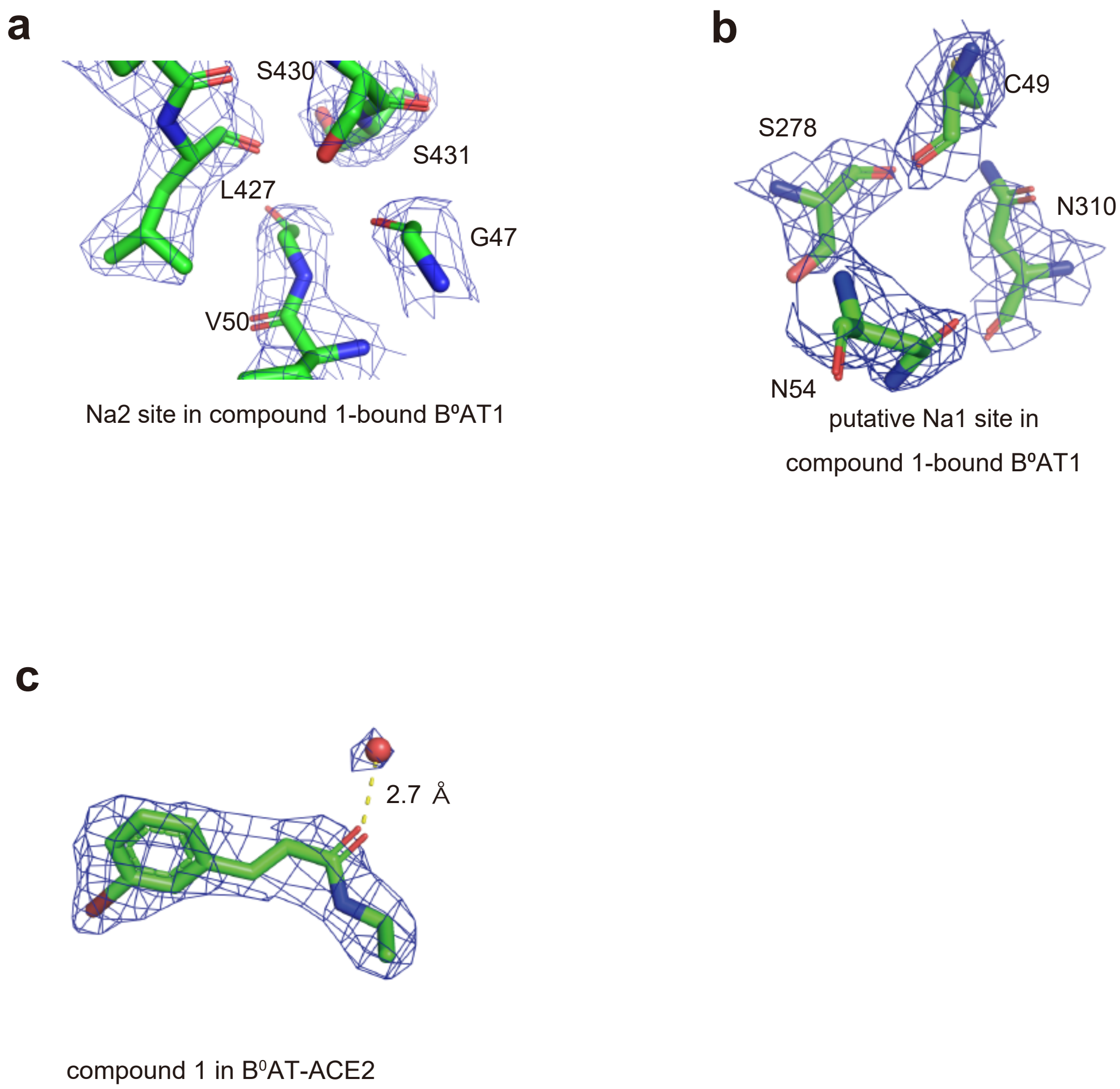

Supplementary Figure 2 Cryo-EM density maps of Na2 and putative Na1 sites in compound 1-bound B^{0}AT1

a) The Na2 site, b) The putative Na1 site of the compound 1-bound structure. c) Compound 1 and its bound water molecules. Contour levels of cryo-EM density are 2.0σ (a-c).

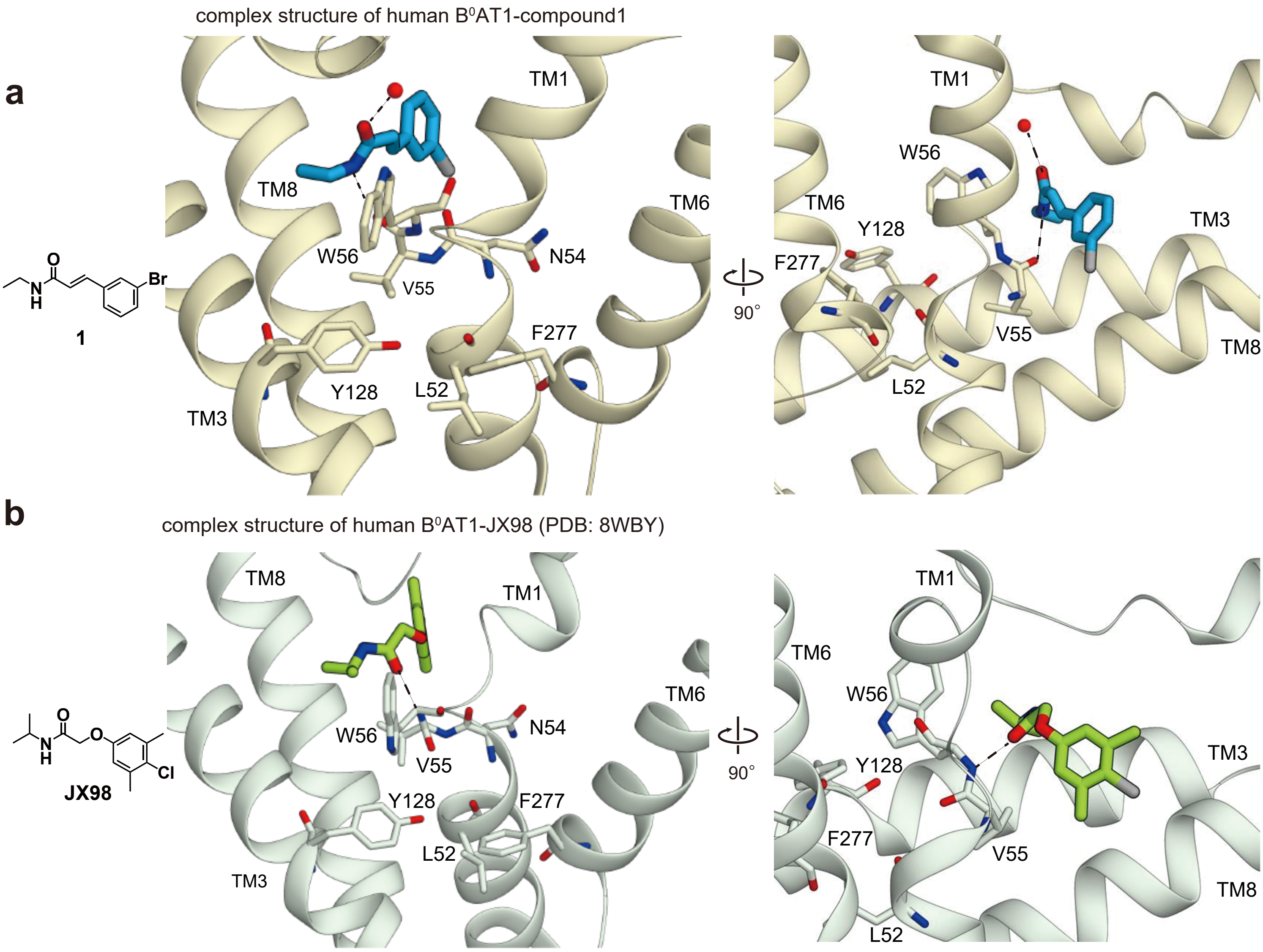

Supplementary Figure 3 Comparison of binding sites for compound 1, and JX98

a) The amide group of compound 1 forms a hydrogen bond with V55. b) The carbonyl of JX98 directs to W56 to form a hydrogen bond. The complex structure of human B^0AT1-JX98 was generated using PDB: 8WBY.

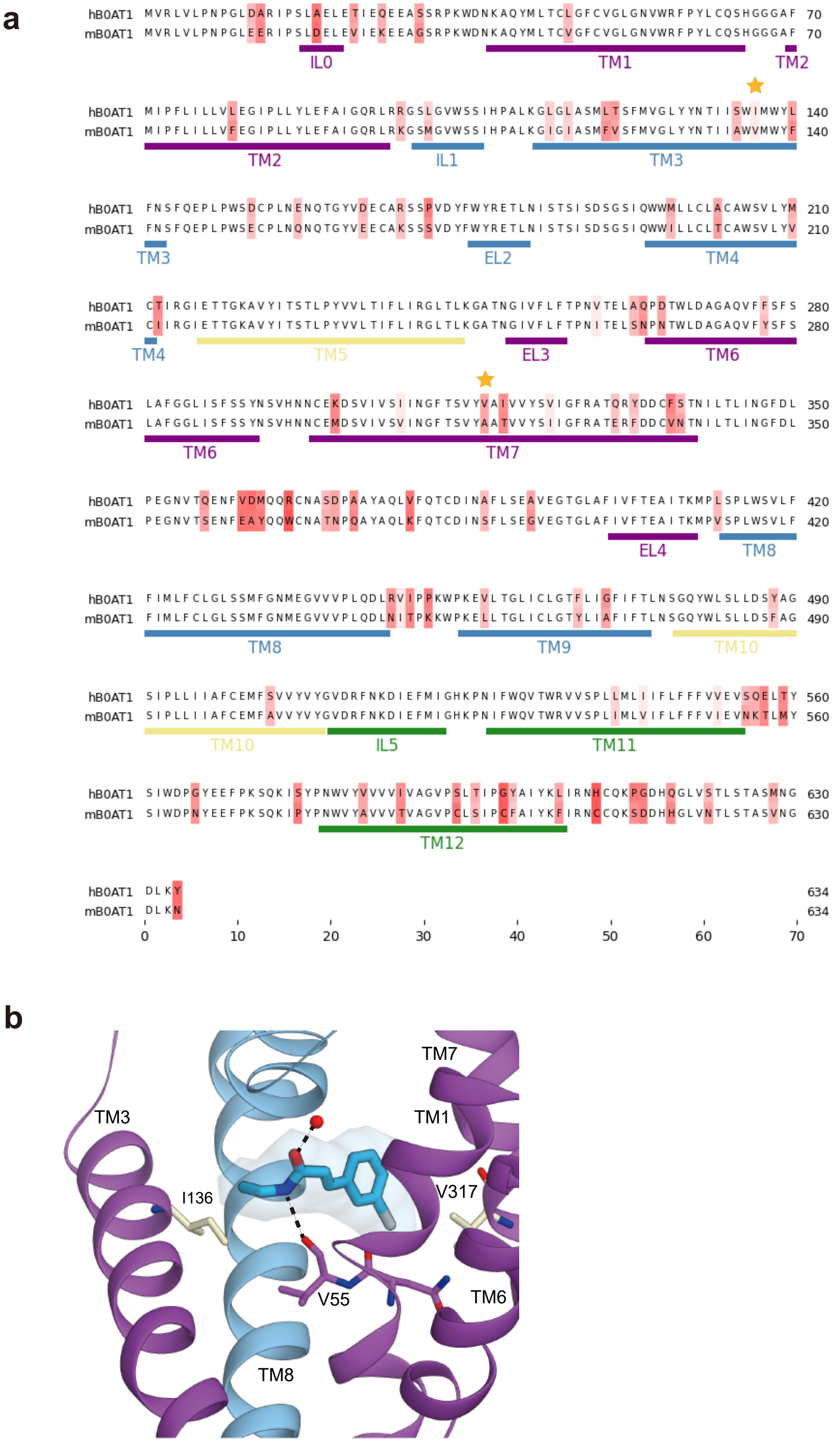

Supplementary Figure 4 Amino acid sequence comparison between human and mouse

a) Amino acid sequence comparison between human and mouse. The star represents the amino acids that differ between human B⁰AT1 and mouse B⁰AT1 around the compound 1 binding site. b) Complex structure of human B⁰AT1-compound 1. Amino acids around the binding site of compound 1 that differ from mouse are indicated in white.

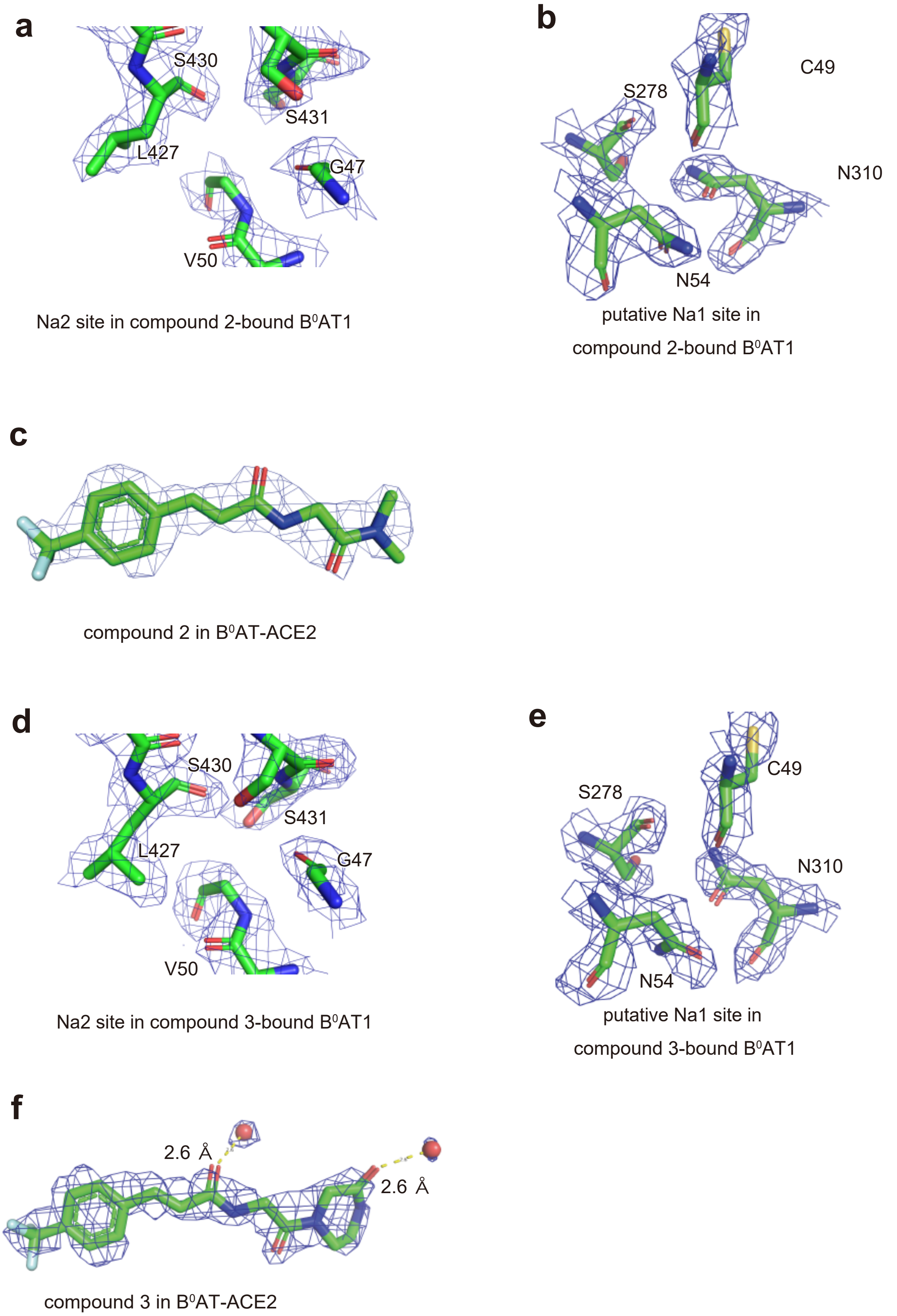

Supplementary Figure 5 Cryo-EM density maps of Na2, putative Na1 sites and compounds in compound 2, 3-bound B^0AT1 structures a) The Na2 site, b) The putative Na1 sites in compound 2-bound B^0AT1 and c) Compounds 2 and its bound water molecules. d) The Na2 site, e) The putative Na1 sites in compound 3-bound B^0AT1 and f) Compounds 3 and its bound water molecules. Contour levels of cryo-EM density are 2.0σ.

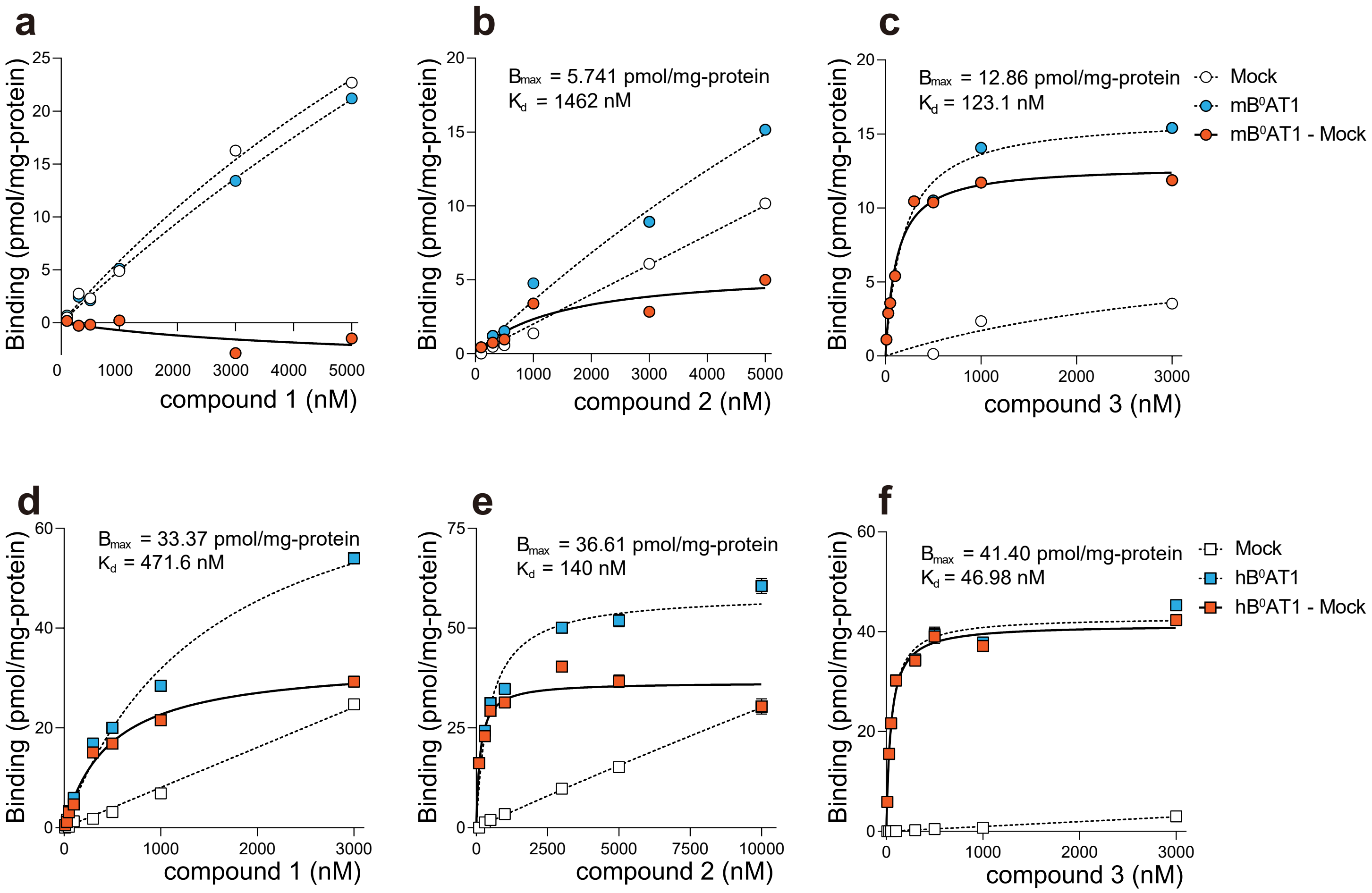

Supplementary Figure 6 Comparison of binding affinity of compound 1-3 to human and mouse B^{0}AT1

a-c) Binding affinity of compound 1-3 using Mock cell and mouse B^{0}AT1 and ACE2-expressing cell. d-f) Binding affinity of compound 1-3 using Mock cell and human B^{0}AT1 and ACE2-expressing cell. Specific binding (black) was calculated by subtracting the binding of mock cells (white) from the binding of B^{0}AT1 and ACE2 expressing cells (gray). Each point represents the mean ± SEM (n = 3, technical replicates).

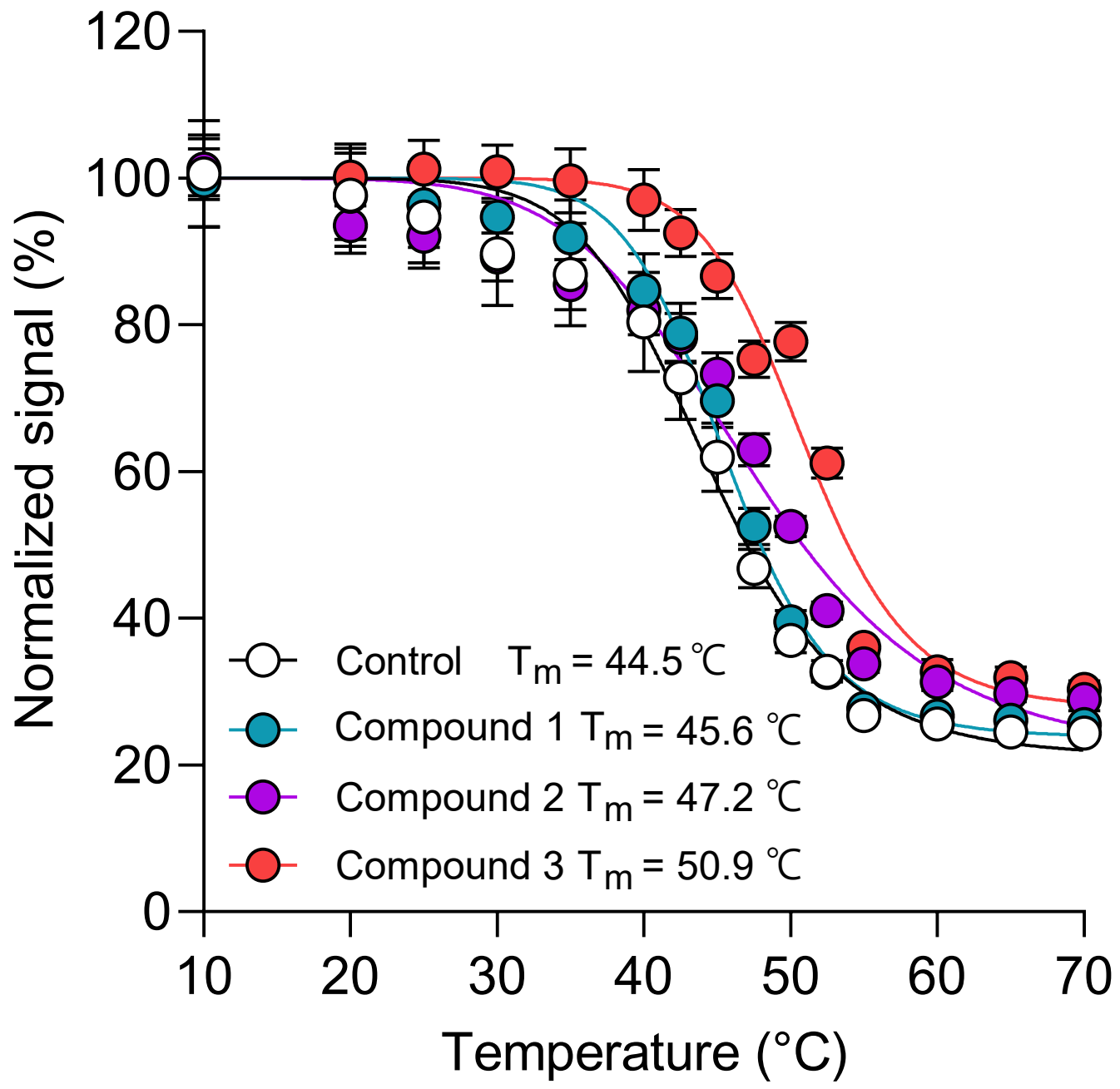

Supplementary Figure 7 Confirmation of thermal stabilization effect using FSEC

FSEC-based thermal shift assay using purified human B^0AT1-ACE2 in the presence or absence of inhibitors. Each point represents the mean ± SEM (n = 3, technical replicates).

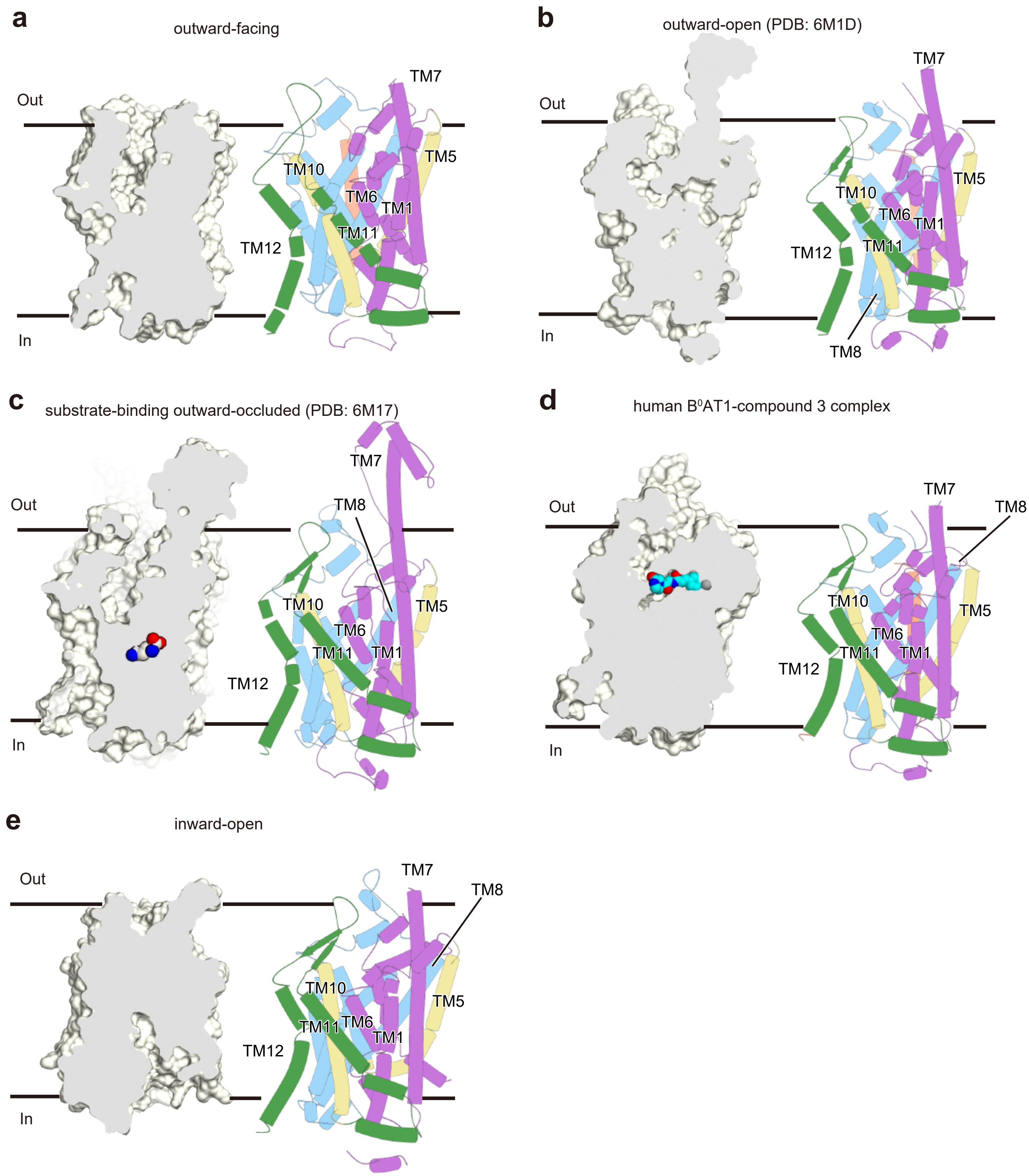

Supplementary Figure 8 Comparing structure of state for human B^0AT1

a) Outward-facing. b) Outward open. c) Substrate-binding outward-occluded. d) Compound1-bound outward-facing. e) Antibody-bound inward-open structures. For the cylindal structures, the bundle domain (TM1,2,6,7) is in purple, the hash domain (TM3,4,8,9) in blue, the gate helices (TM5,10) in yellow, the rest of the protein (TM11,12) in green. The cavity for compound binding appears only in an outward-open conformation when there is no compound.

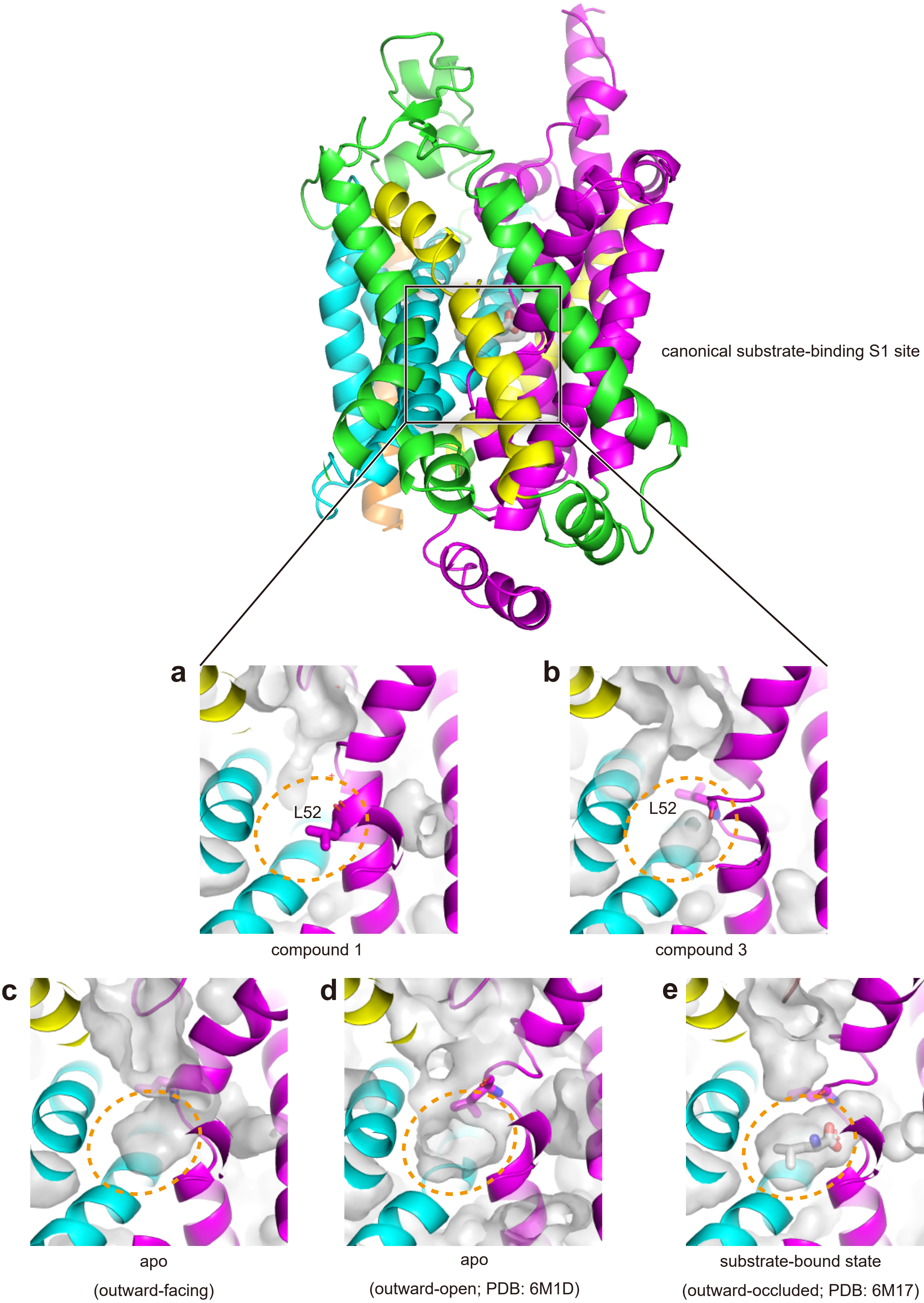

Supplementary Figure 9 Structure of canonical substrate binding S1 sites in each state

a) When compound 1 binds, the side chain of L52 flips, and the substrate binding site is completely lost. b) In the case of compound 3, the side chain of L52 does not flip, so an occluded space remains. c, d) The outward-facing, outward-open structure indicates that the substrate binding site is connected to the outside of the cell. e) The substrate binding state indicates that the substrate is in the occluded space.

a

	TM1a – TM1b	TM3	TM5
SLC6A19	51 GLGNVWRFPYLCQS 64	130 NTIISWIMW 138	228 TLPYVVLTIF 237
SLC6A1	63 GLGNVWRFPYLCGK 76	141 IVIISWAIY 149	245 TYPYIMLIIL 254
SLC6A3	79 DLANVWRFPYLCYK 92	157 NVIIAWALH 165	271 TMPYVVLTAL 280
SLC6A4	98 DLGNVWRFPYICYQ 111	177 NTIMAWALY 185	286 TFPYIILSVL 295
SLC6A18	36 GLGNIWRFPYLCQT 49	116 NTIVAWVLW 124	214 LFPYLVLTIF 223
SLC6A20	24 GLGNVWRFPYLCQM 37	103 NVINAWAFW 111	201 SLPYCVLIIY 210
LeuT	20 GNAVGLGNFLRFPV 33	109 VYIESWTLG 117	199 MPTLFILAVF 208
SLC5A2	77 GSGHFVGLAGTGAA 90	159 MFSGAVFIQ 167	205 TFVILGGACI 214

	TM7	EL4b – TM8
SLC6A19	313 TSVYVAIVVY 322	393 VEGTGLAFIVFTEAITKMPLSPLWSVLFFIMLFCL 424
SLC6A1	330 TSMFAGFVIF 339	359 -SGPGLAFLAYPEAVTQLPISPLWAILFFSMLLML 392
SLC6A3	356 TSFSSGFVVF 365	384 D-GPGLIFIIYPEAIATLPLSSAWAVVFFIMLLTL 418
SLC6A4	371 TSFVSGFVIF 380	400 DAGPSLLFITYAEAIANMPASTFFAIIFFLMLITL 434
SLC6A18	299 TSLYASIAVF 308	379 ASGPGLAFVVFTETDLHMPGAPVWAMLFFGMLFTL 413
SLC6A20	286 TSIFASIVTF 295	368 VQGTGLAFIVYTEAIKNMEVSQLWSVLYFFMLLML 402
LeuT	289 AEVILGGSIS 298	317 AGAFNLGFITLPAIFSQTAGGTFLGFLWFFLLFFA 351
SLC5A2	321 KLTPMFLMVM 330	360 -----GCSNIAYPRLVVKLMPNGLRGLMLAVMLAA 389

b

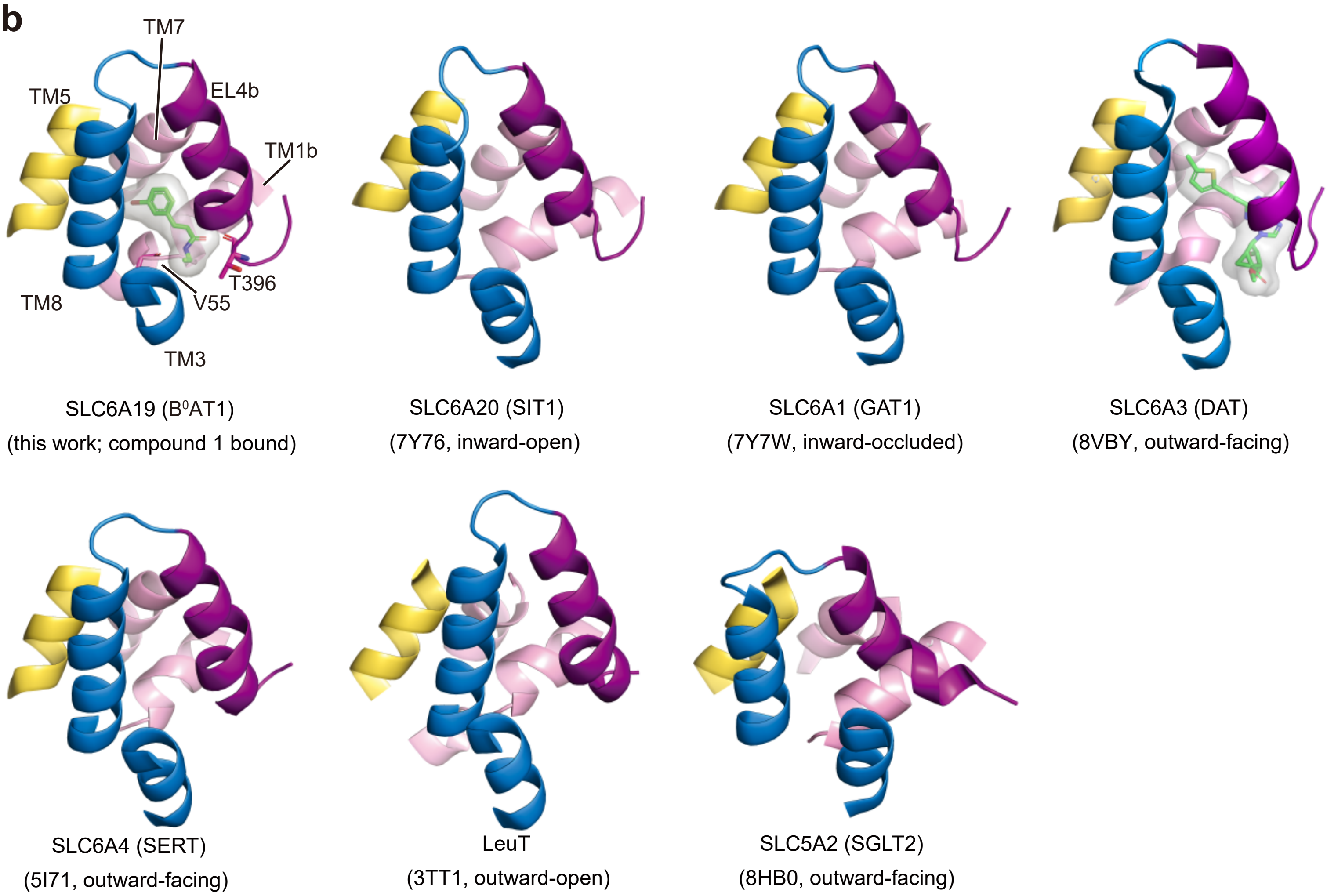

Supplementary Figure 10 Comparison of the allosteric sites among SLC5 and SLC6 transporters

a) Sequence comparison: The six α-helices that make up the site are conserved in all the transporters. b) Structural comparison: The binding site formed by the six α-helices is conserved in all transporters. Other than B^0AT1, it has been reported that a compound is bounded to the same site of DAT (SLC6A3).

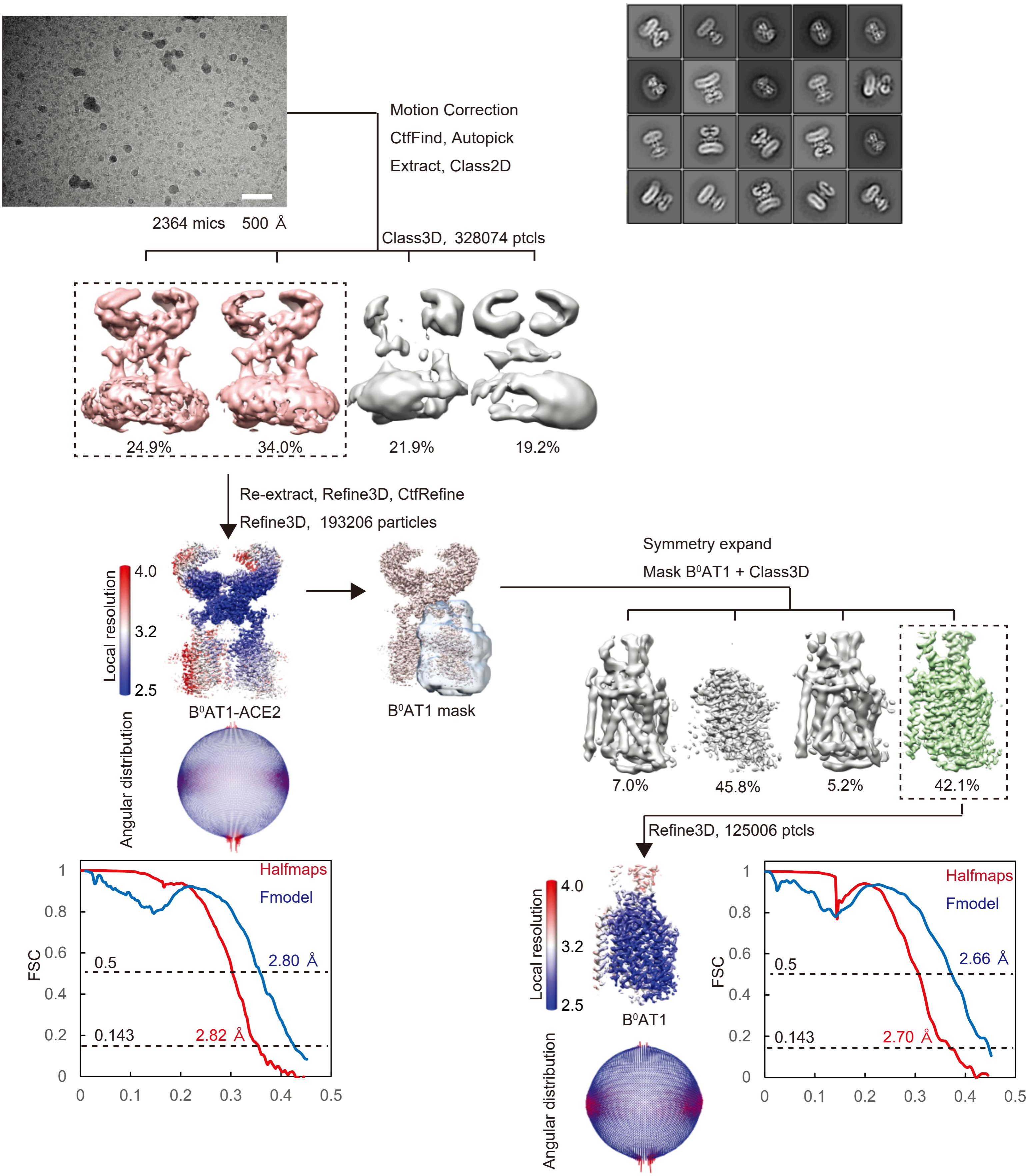

Supplementary Figure 11 Structure determination of the compound 1 bound B^0AT1-ACE2

Flow chart for determining the structure of the B^0AT1-ACE2 complex in the presence of compound 1.

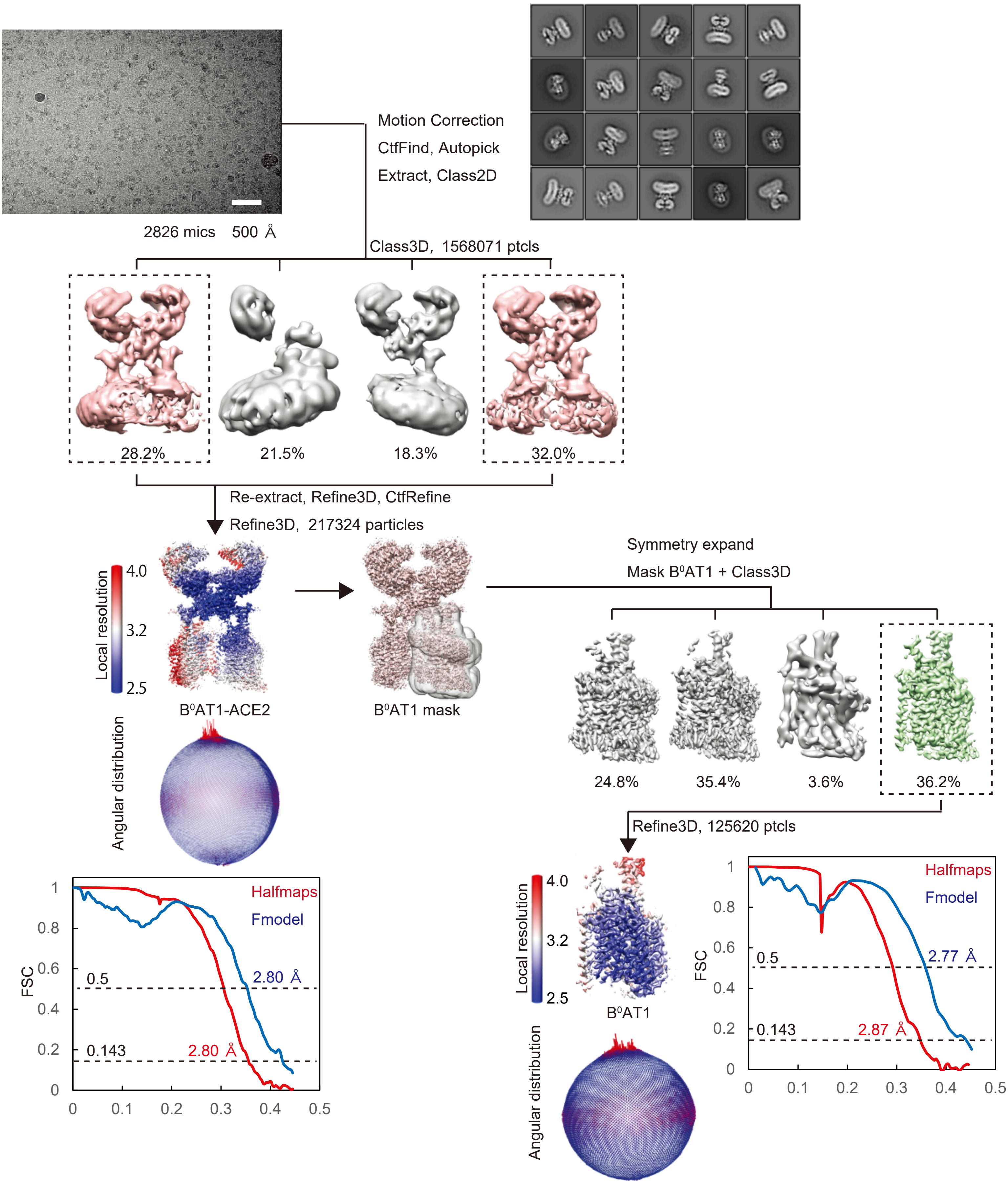

Supplementary Figure 12 Structure determination of the compound 2 bound B⁰AT1-ACE2

Flow chart for determining the structure of the B⁰AT1-ACE2 complex in the presence of compound 2.

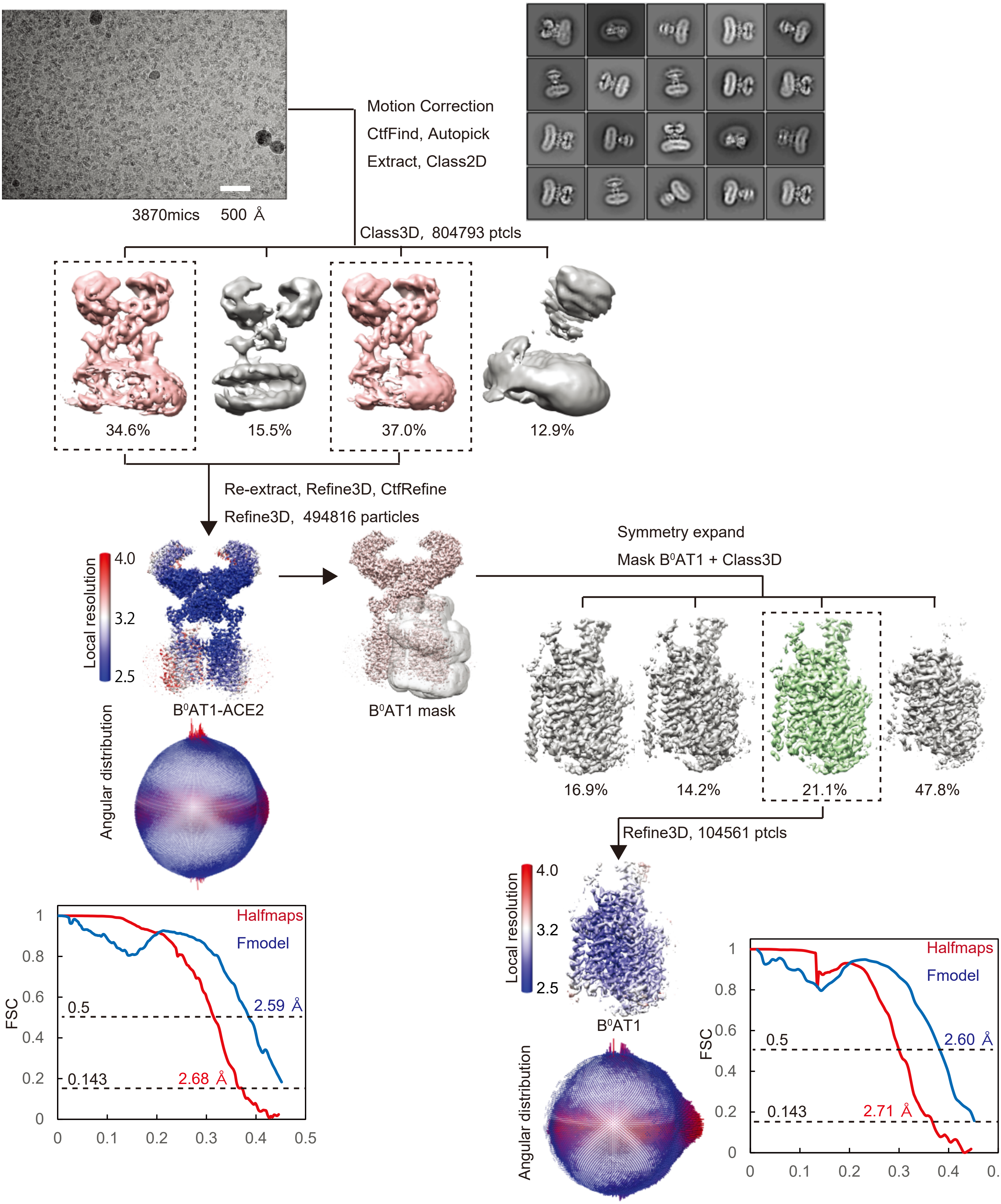

Supplementary Figure 13 Structure determination of the compound 3 bound B⁰AT1-ACE2

Flow chart for determining the structure of the B⁰AT1-ACE2 complex in the presence of compound 3.

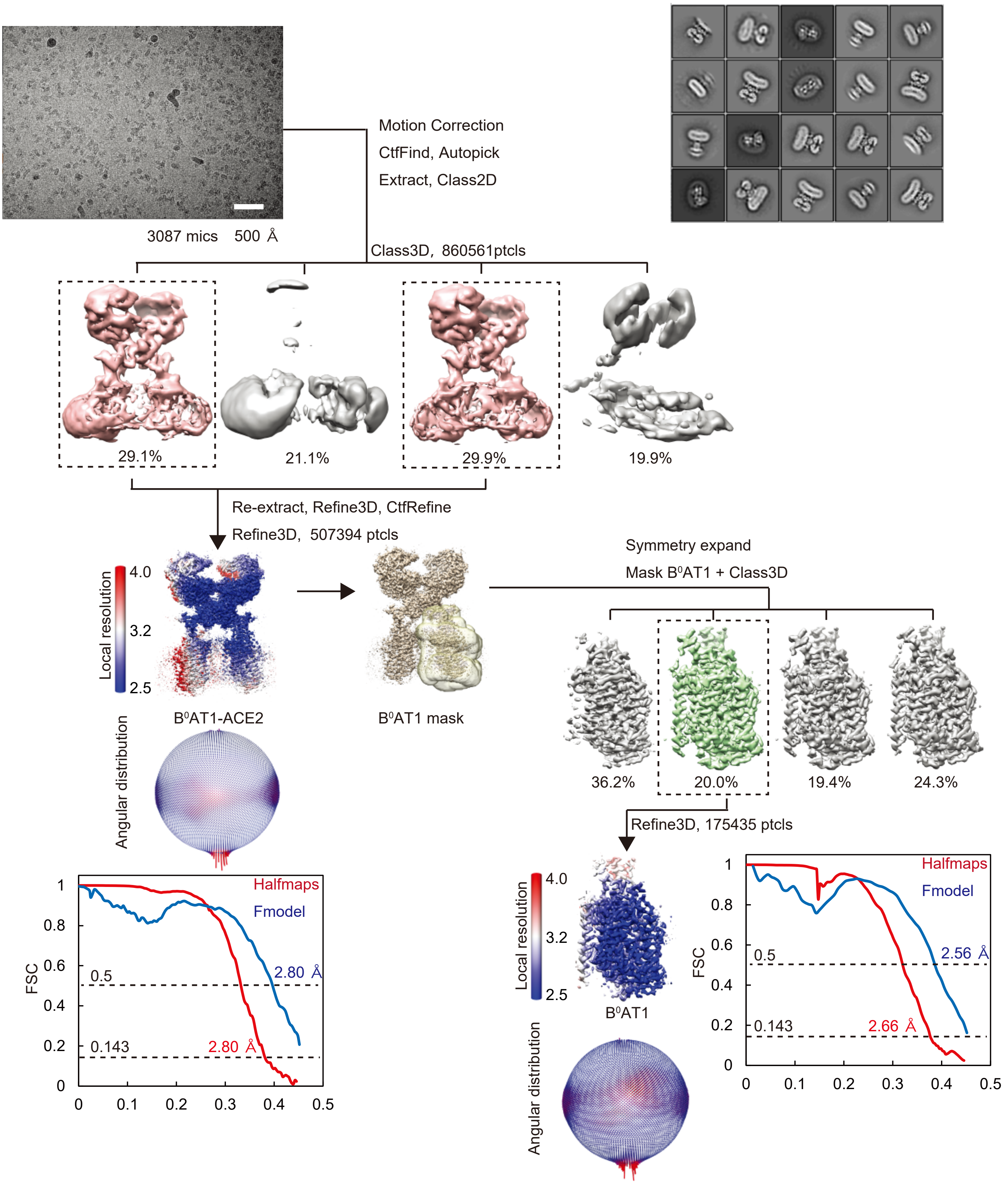

Supplementary Figure 14 Structure determination of apo B^0AT1-ACE2

Flow chart for determining the structure of the B^0AT1-ACE2 complex.

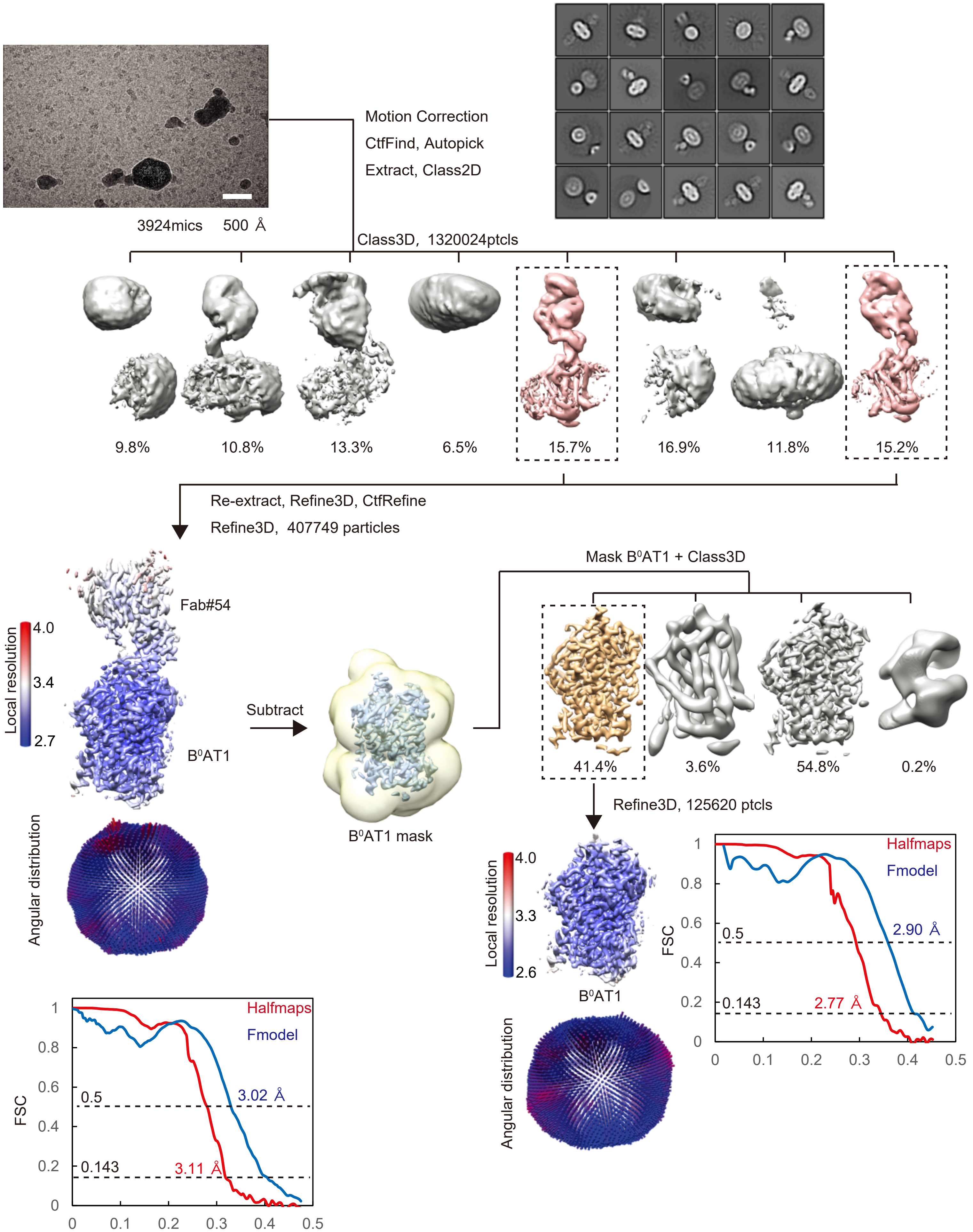

Supplementary Figure 15 Structure determination of the inward-open form of apo B^0AT1-Fab#54

Flow chart for determining the structure of the inward-open form of apo B^0AT1-Fab#54 complex.

a TMs of B⁰AT1-ACE2 bound with compound 1

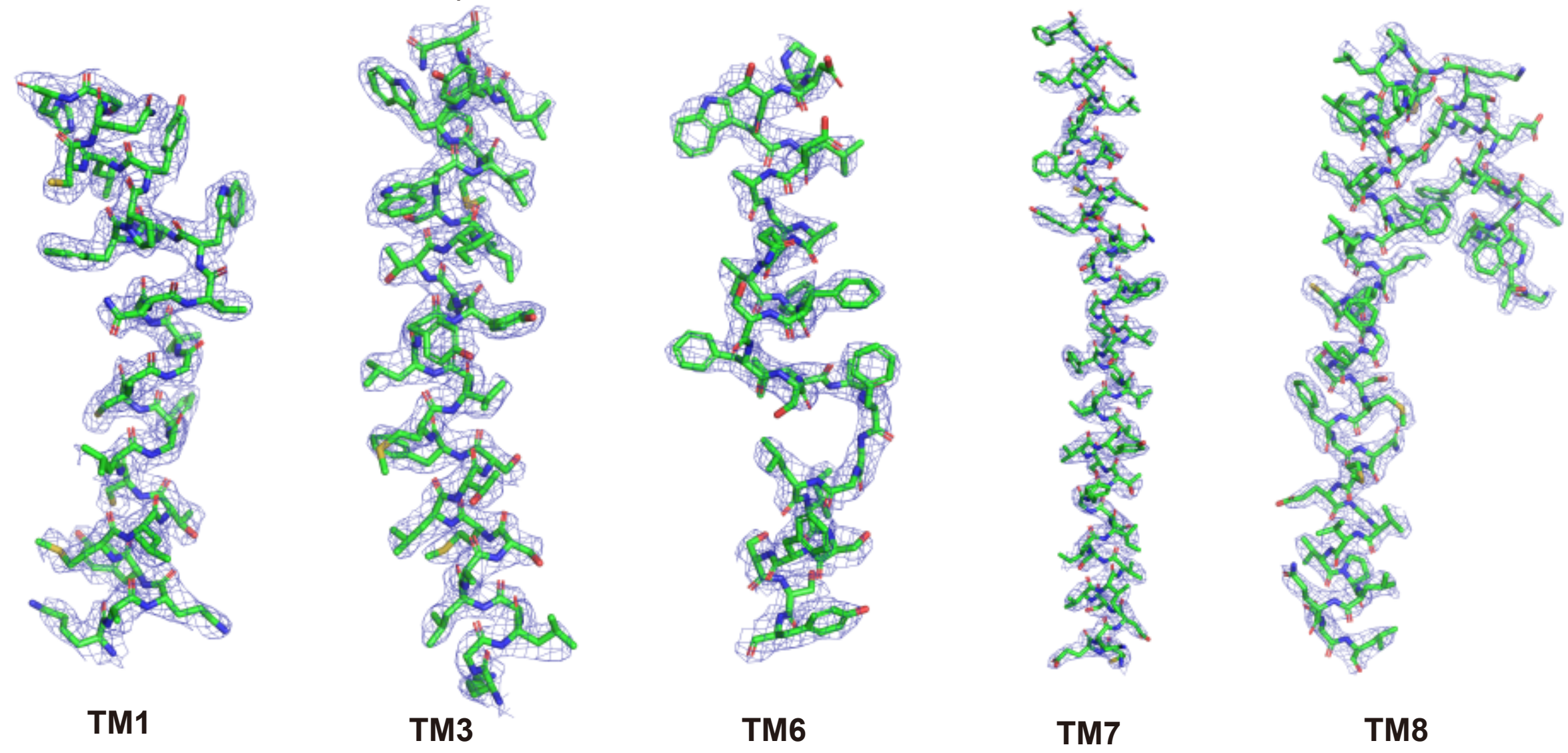

b TMs of B⁰AT1-ACE2 bound with compound 2

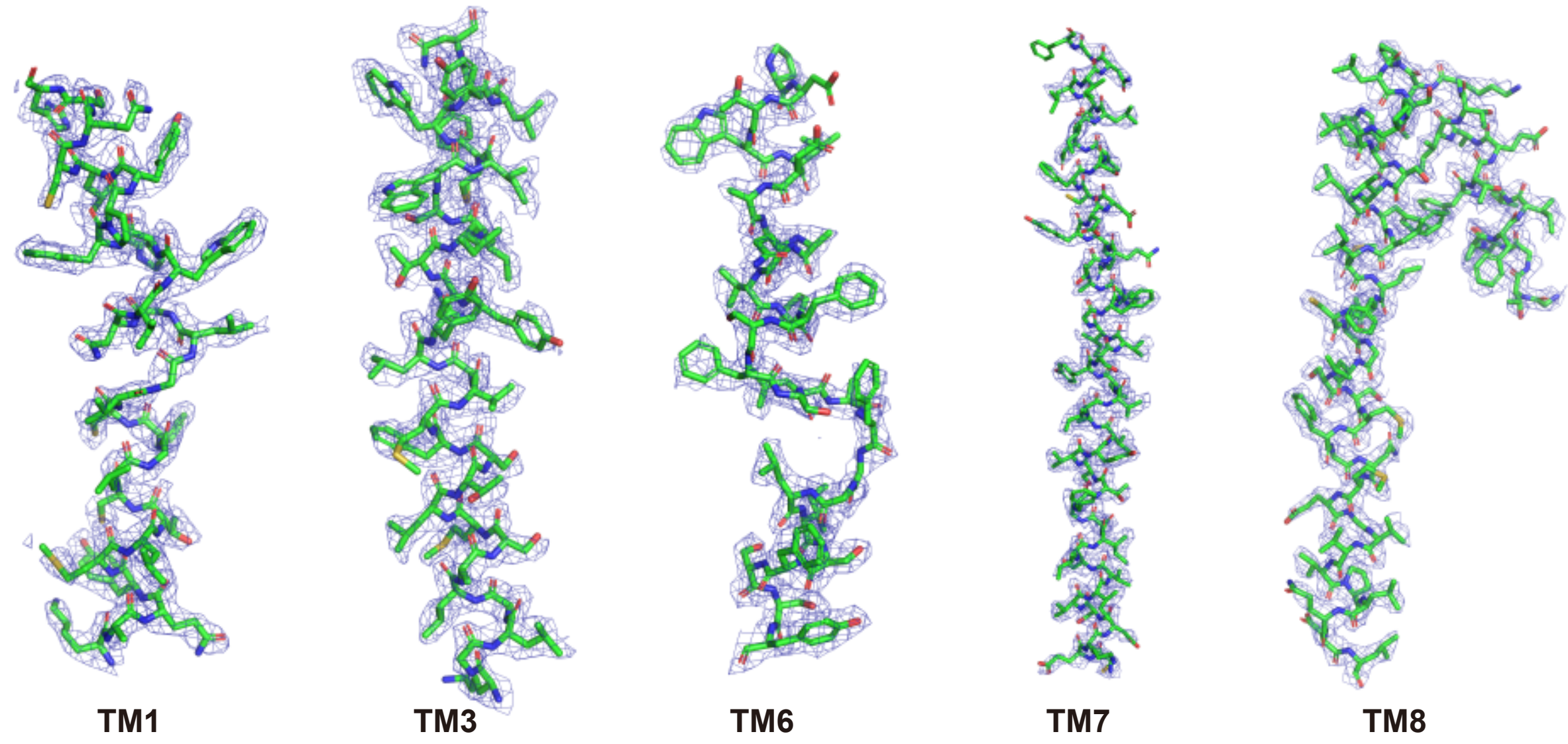

c TMs of B⁰AT1-ACE2 bound with compound 3

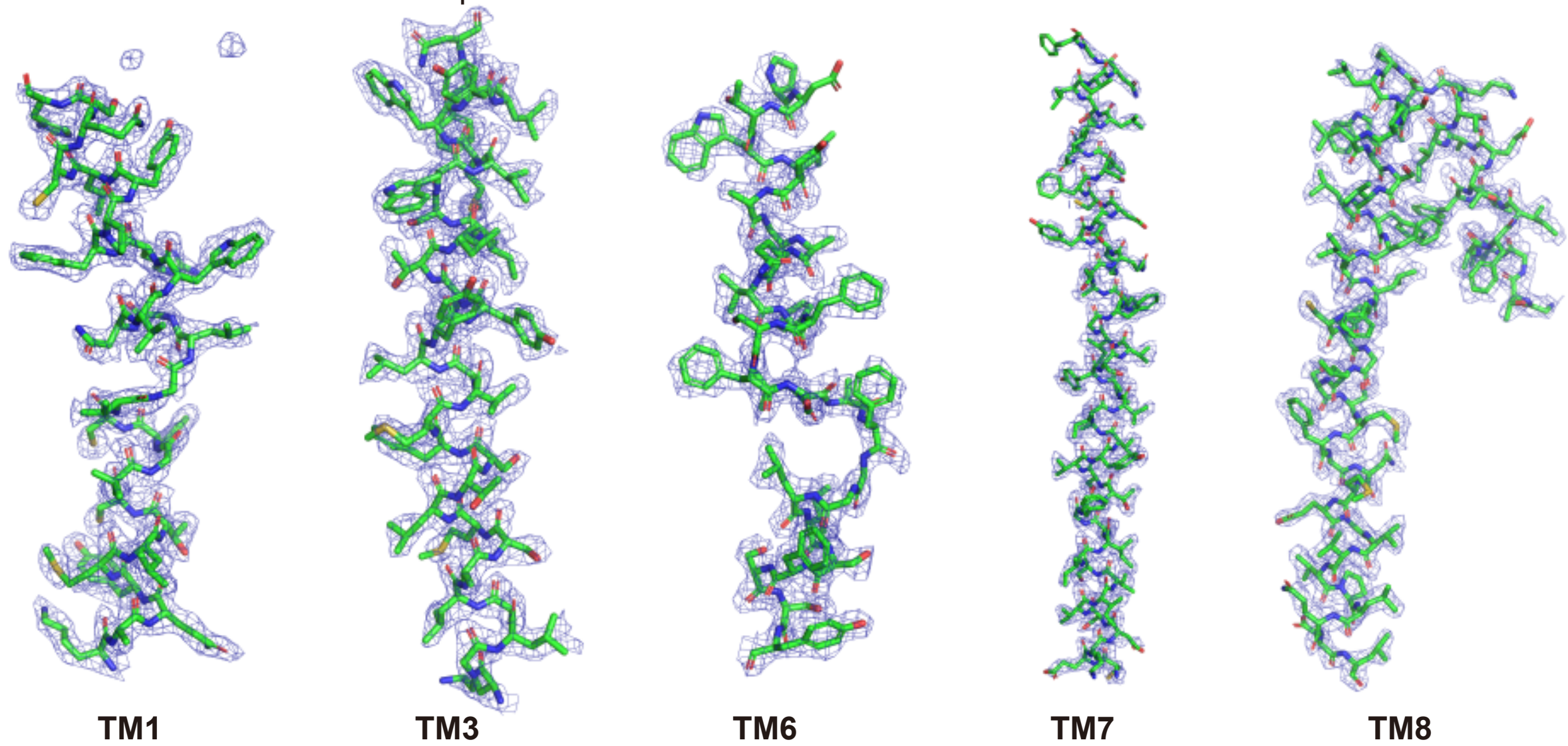

Supplementary Figure 16 Cryo-EM density maps of B⁰AT1-ACE2 bound with inhibitors

Cryo-EM density maps shown at threshhold of 2σ (a-c).

a TMs of the outward-facing apo B⁰AT1-ACE2

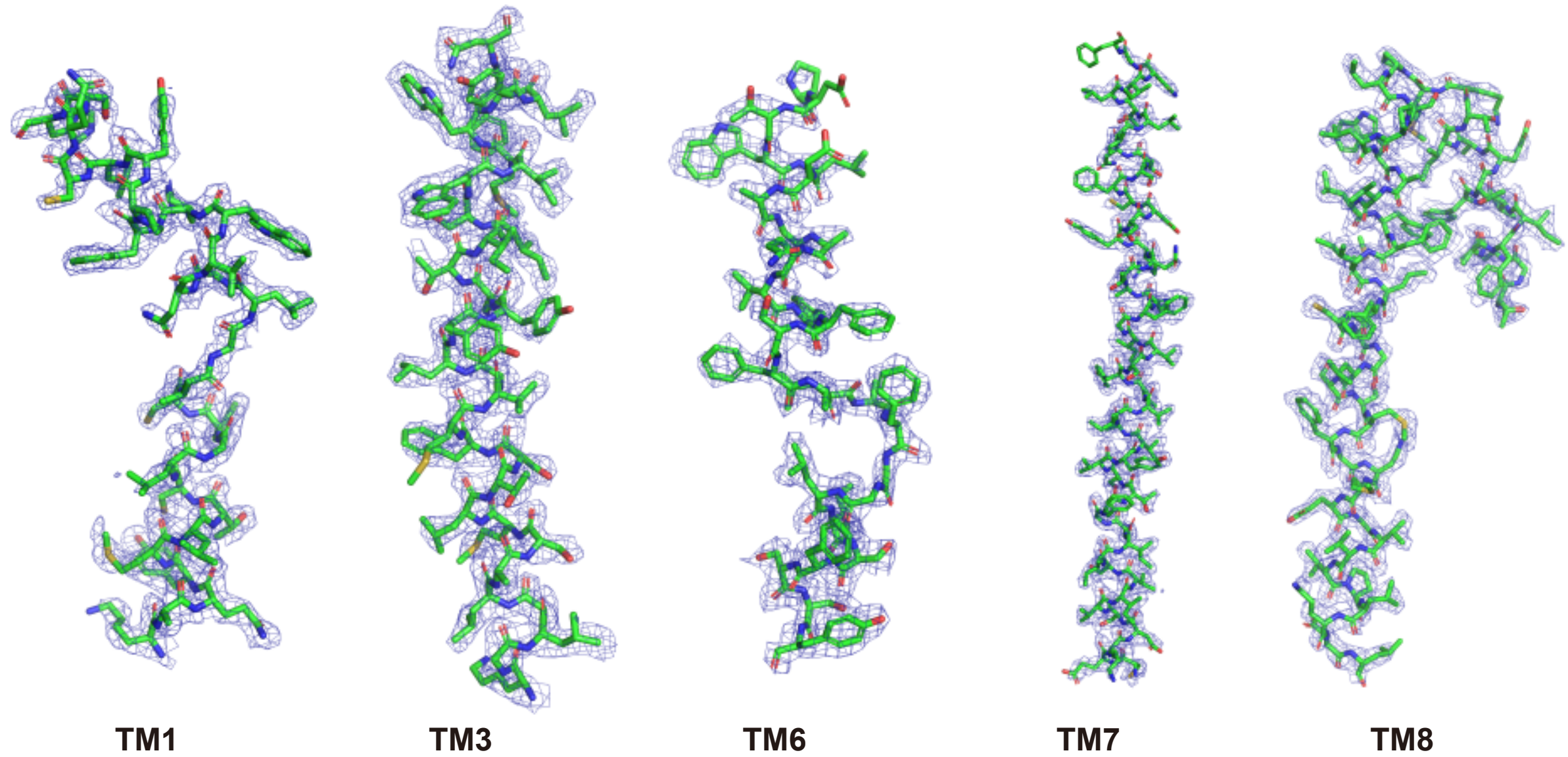

b TMs of the inward open apo B⁰AT1-Fab#54

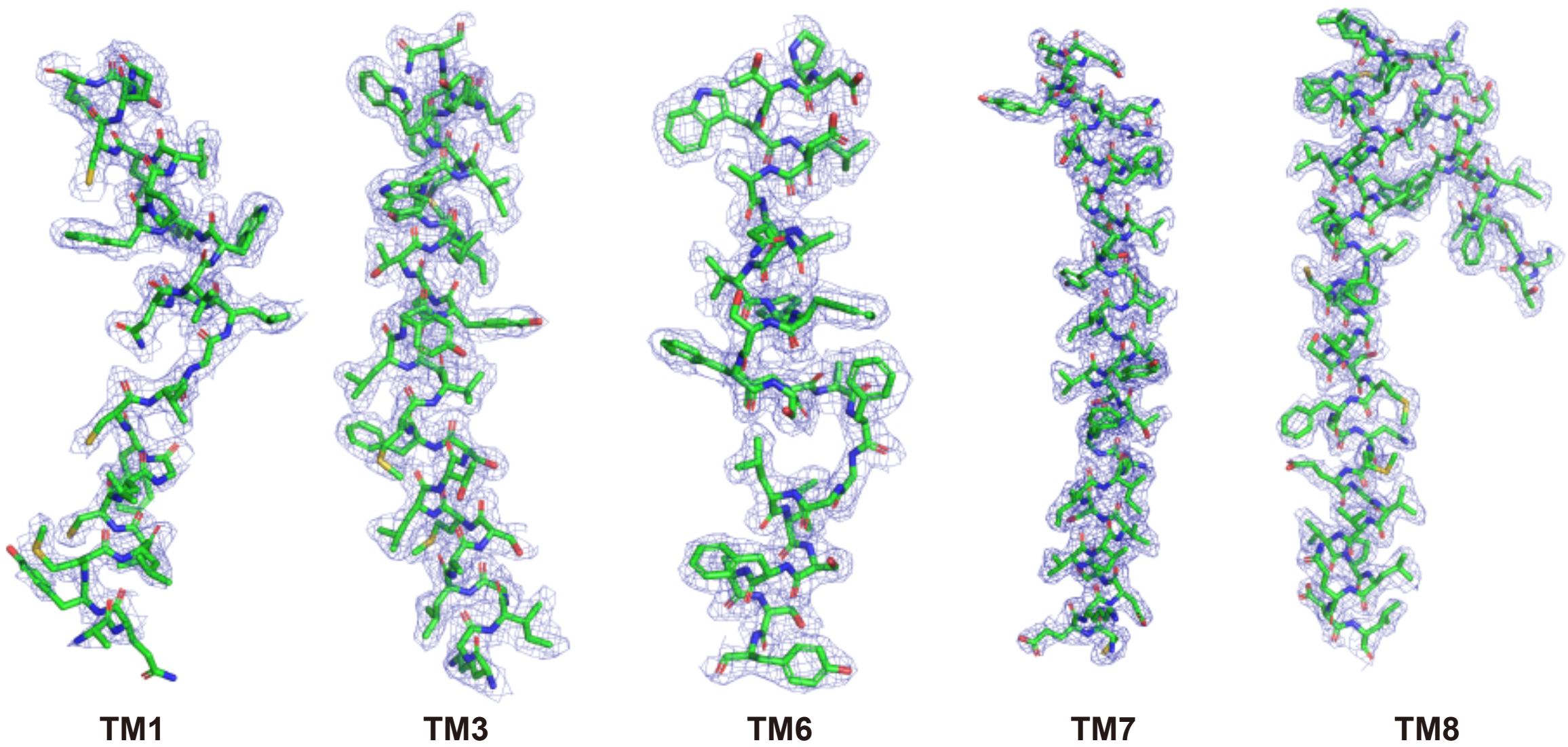

Supplementary Figure 17 Cryo-EM density maps of apo B⁰AT1-ACE2 and apo B⁰AT1-Fab#54

Contour levels of cryo-EM density are 1.0σ (a-b).

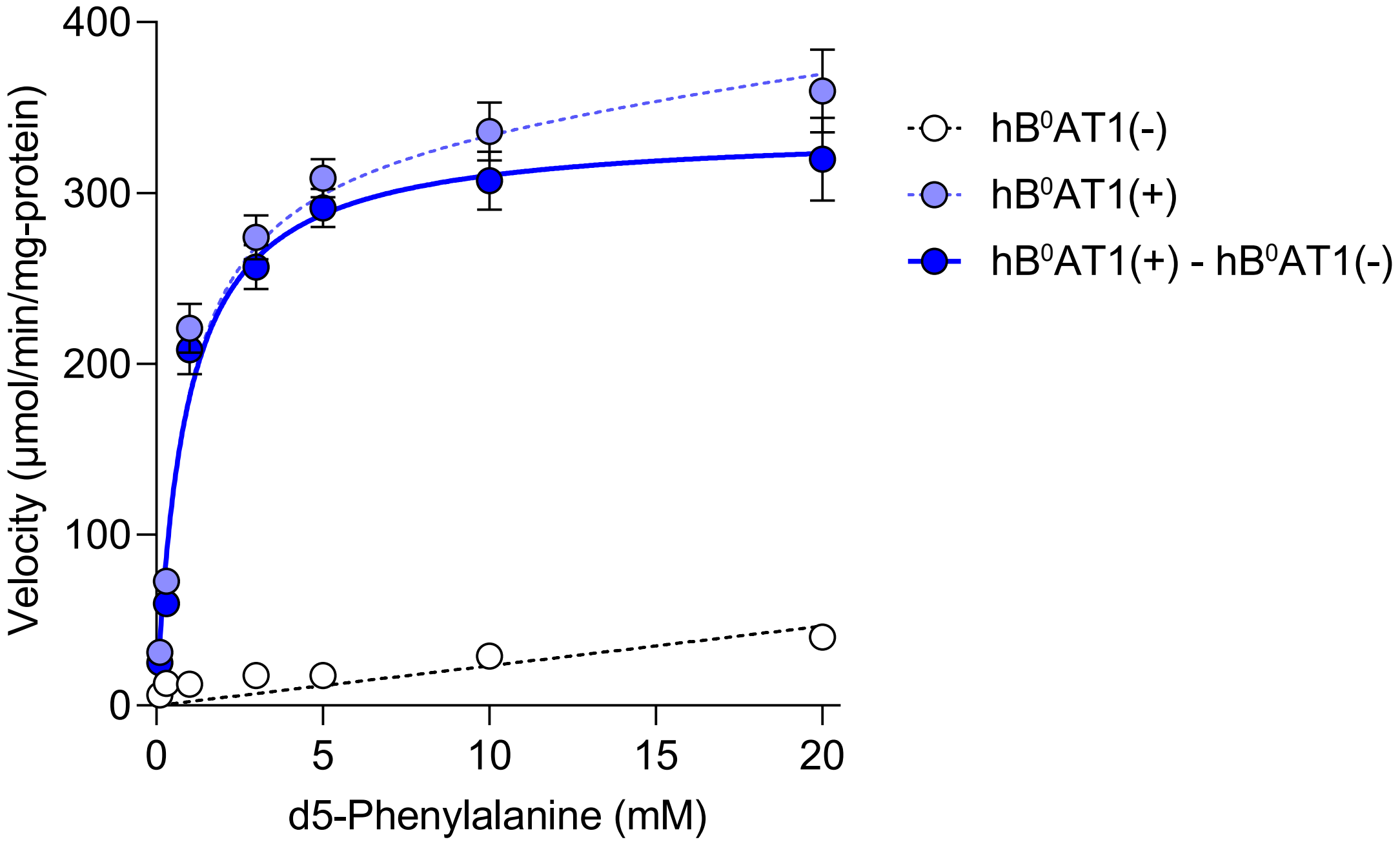

Supplementary Figure 18 Concentration dependence of d5-phenylalanine uptake by hB^0AT1 and ACE2-expressing cells and non-expressing cells

B^0AT1-mediated uptake was calculated by subtracting the uptake by non-expressing cells from that of hB^0AT1 and ACE2-expressing cells. Each point represents the mean ± SEM (n = 3, biological replicates).

Supplementary Methods Experimental procedures for compound synthesis

General Methods for Chemistry. The progress of the reactions was monitored using analytical thin layer chromatography (TLC) on glass plates (TLC Silica gel 60 F254), and the products were visualized with UV light or LC/MS (Waters, ACQUITY QDa or ACQUITY SQD). For column chromatography, silica gel (Wakogel® 60 N, FUJIFILM Wako, Osaka, Japan) or a pre-packed silica gel column (Hi-Flash-M, L, 2L, 3 L or 5L, YAMAZEN, Osaka, Japan) was used. Nuclear magnetic resonance (NMR) spectra were recorded on a Bruker AVANCE 400 spectrometer (400 MHz) at 400 and 100 MHz for ^{1}H and ^{13}C, respectively, and referenced to tetramethylsilane as an internal standard. NMR data were processed using ACD/Spectrus Processor software and recorded as follows: ^{1}H NMR-chemical shift (δ, ppm), multiplicity (s, singlet; d, doublet; t, triplet; q, quartet; m, multiplet), coupling constant (Hz), and integration; ^{13}C NMR-chemical shift (δ, ppm). High-resolution mass spectra (HRMS) were recorded using a Thermo Scientific LTQ Orbitrap XL Exact mass spectrometer. The purity of biologically tested compounds was assessed by ultra-high performance liquid chromatography (UHPLC) analysis at 200-400 nm. The UHPLC analysis was performed on a Shimadzu Nexera X2 system with a Waters ACQUITY UPLC BEH C18 (1.7 μm, 2.1 × 100 mm) column.

Experimental procedure for compound 2, 3.

Schemes

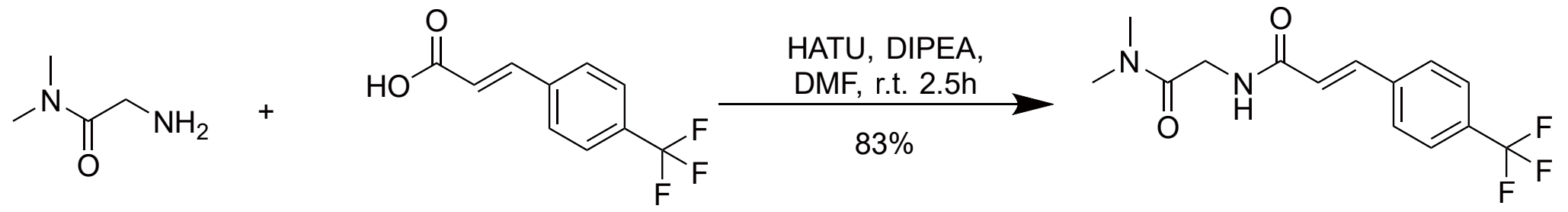

1 **2** **compound 2**

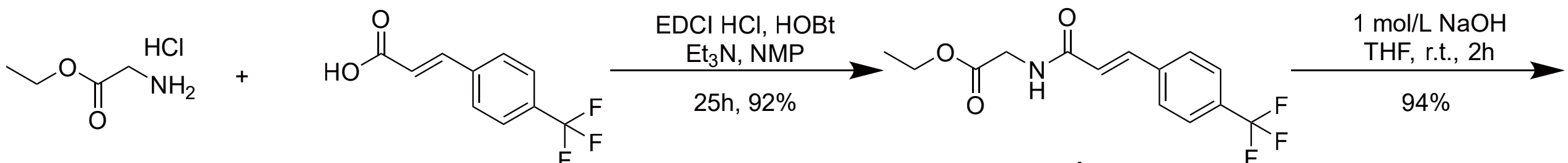

3 **2** **4**

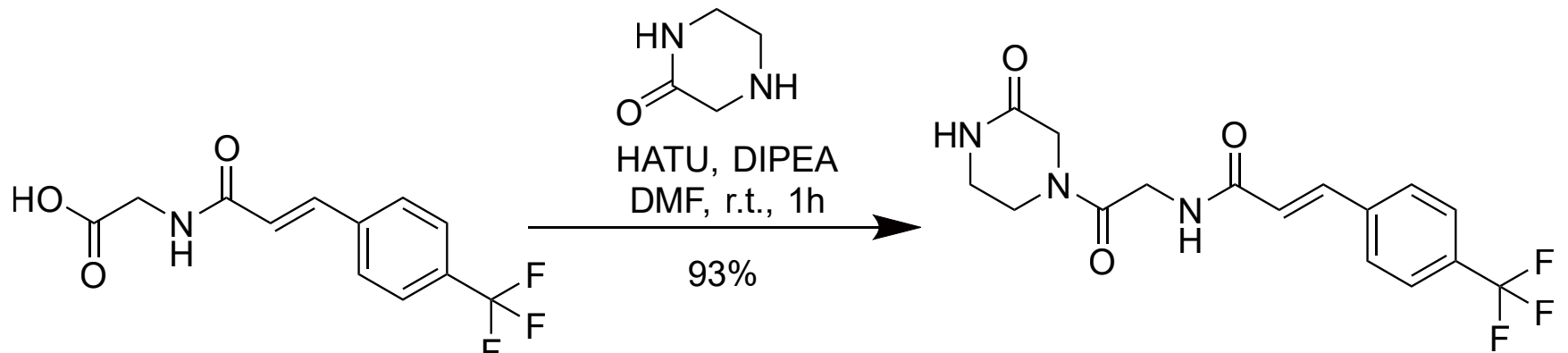

5 **compound 3**

(E)-N-[2-(dimethylamino)-2-oxo-ethyl]-3-[4-(trifluoromethyl)phenyl]prop-2-enamide (compound 2)

(E)-3-[4-(trifluoromethyl)phenyl]prop-2-enoic acid (199 mg, 0.92 mmol, 1.00 eq), 2-amino-N,N-dimethyl-acetamide (116 mg, 1.14 mmol, 1.20 eq), DIPEA(0.19 mL, 1.10 mmol, 1.20 eq) and HATU (391 mg, 1.03 mmol, 1.10 eq) were added to DMF (5 mL). The mixture was stirred for 2.5 h at rt. Then, water was added, precipitated solid was separated with filtration. The filtered compound was washed with water and dried over. White solid, yield 230 mg (0.77 mmol, 83 %).

^{1}H NMR (400 MHz, DMSO-d_6): δ [ppm] = 2.86 (s, 3H), 2.98 (s, 3H), 4.08 (d, J = 5.4 Hz, 2H), 7.00 (d, J = 15.8 Hz, 1H), 7.50 (d, J = 15.8 Hz, 1H), 7.75-7.83 (m, 4H), 8.25 (t, J = 5.4 Hz, 1H). ^{13}C NMR (100 MHz, DMSO-d_6): δ [ppm] = 35.1, 35.7, 40.8, 120.1, 122.8, 124.9, 125.5, 125.8, 128.2, 129.2, 137.2, 139.0, 164.6, 168.0. HRMS (ESI): m/z = 301.1158 (calcd. 301.1157 for $C_{14}H_{16}F_3N_2O_2$ [M+H]$^+$). Purity (HPLC): 99.8 %.

Compound 2 ^{1}H NMR (400 MHz, DMSO-d_6)

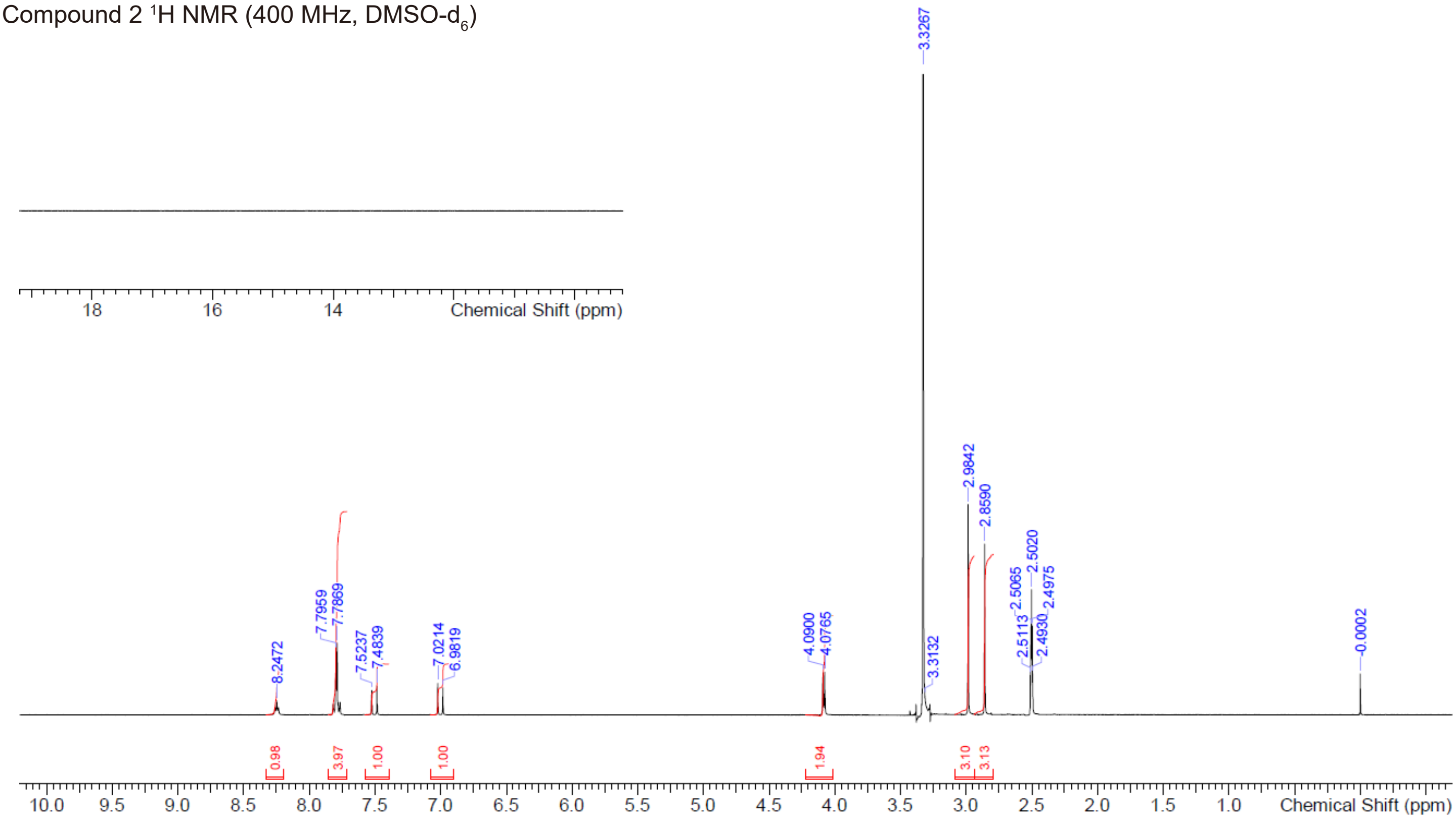

Compound 2 ^{13}C NMR (100 MHz, DMSO-d₆)

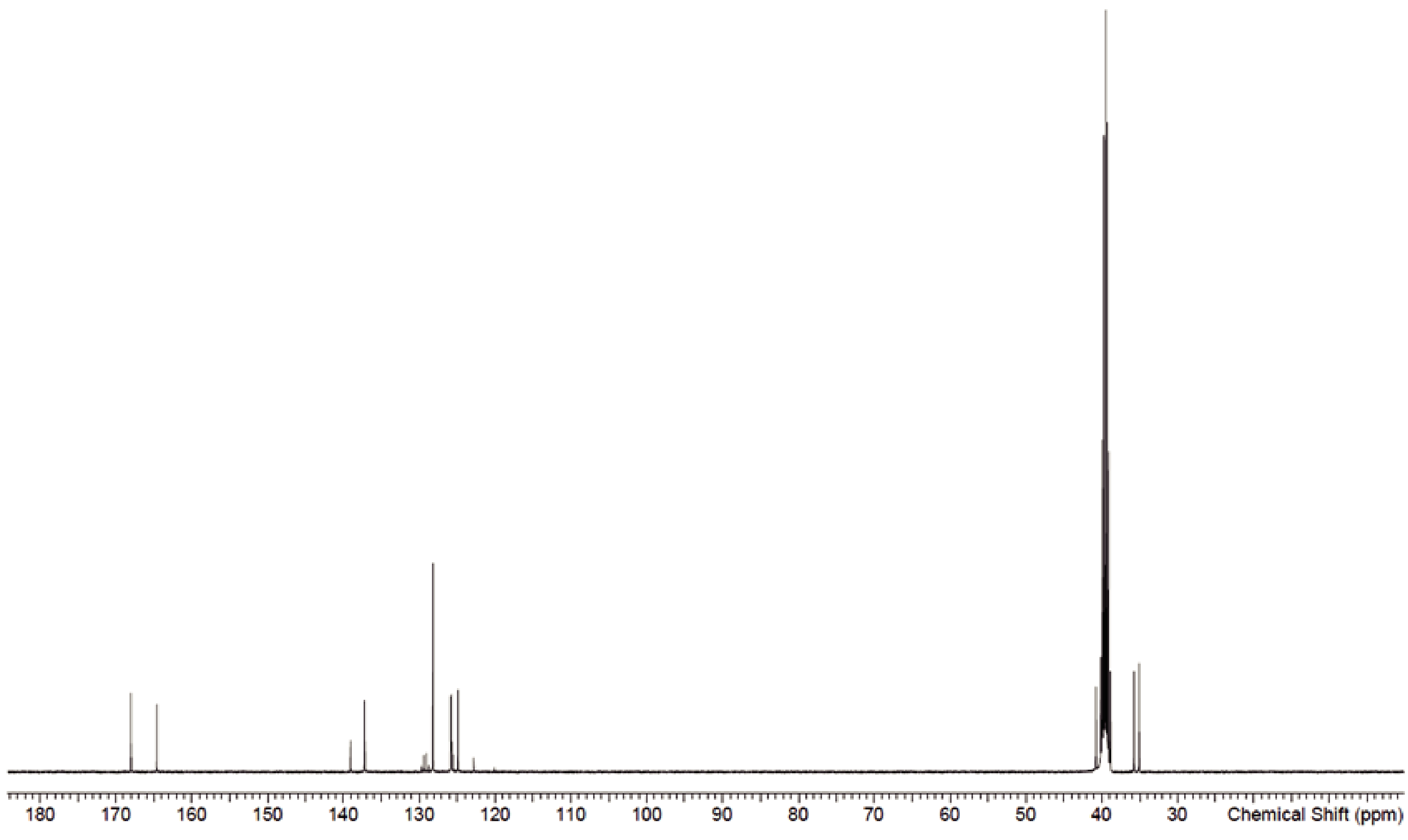

Ethyl 2-[[(E)-3-[4-(trifluoromethyl)phenyl]prop-2-enoyl]amino]acetate (4)

(E)-3-[4-(trifluoromethyl)phenyl]prop-2-enoic acid (105 g, 485.75 mmol, 1.00 eq), ethyl 2-aminoacetate;hydrochloride (72.1 g, 517 mmol, 1.10 eq), Et_3N (150 mL, 1080 mmol, 2.20 eq), WSC HCl (100 g, 521.65 mmol, 1.10 eq) and HOBt (72.2 g, 534 mmol, 2.20 eq) were added to NMP (800 mL). The mixture was stirred for 25 h at rt. Then, water (3 L) was added, precipitated solid was separated with filtration. The filtered compound was washed with water and dried over. White solid, yield 135 g (448.10 mmol, 92 %).

^{1}H NMR (400 MHz, $CDCl_3$): δ [ppm] = 1.33 (t, J = 7.2 Hz, 3H), 4.20 (d, J = 5.0 Hz, 2H), 4.27 (q, J = 7.2 Hz, 2H), 6.20 (t, J = 5.0 Hz, 1H), 6.55 (d, J = 15.6 Hz, 1H), 7.60-7.68 (m, 4H), 7.68 (d, J = 15.6 Hz, 1H). ^{13}C NMR (100 MHz, DMSO-d_6): δ [ppm] = 14.4, 41.4, 61.2, 120.4, 123.1, 124.3, 125.8, 126.8, 128.8, 129.9, 138.5, 139.0, 165.8, 170.3. HRMS (ESI): m/z = 302.0999 (calcd. 302.0997 for $C_{14}H_{15}F_3NO_3$ $[M+H]^+$).

Ethyl 2-[[(E)-3-[4-(trifluoromethyl)phenyl]prop-2-enoyl]amino]acetate (4) ^{1}H NMR (400 MHz, $CDCl_3$)

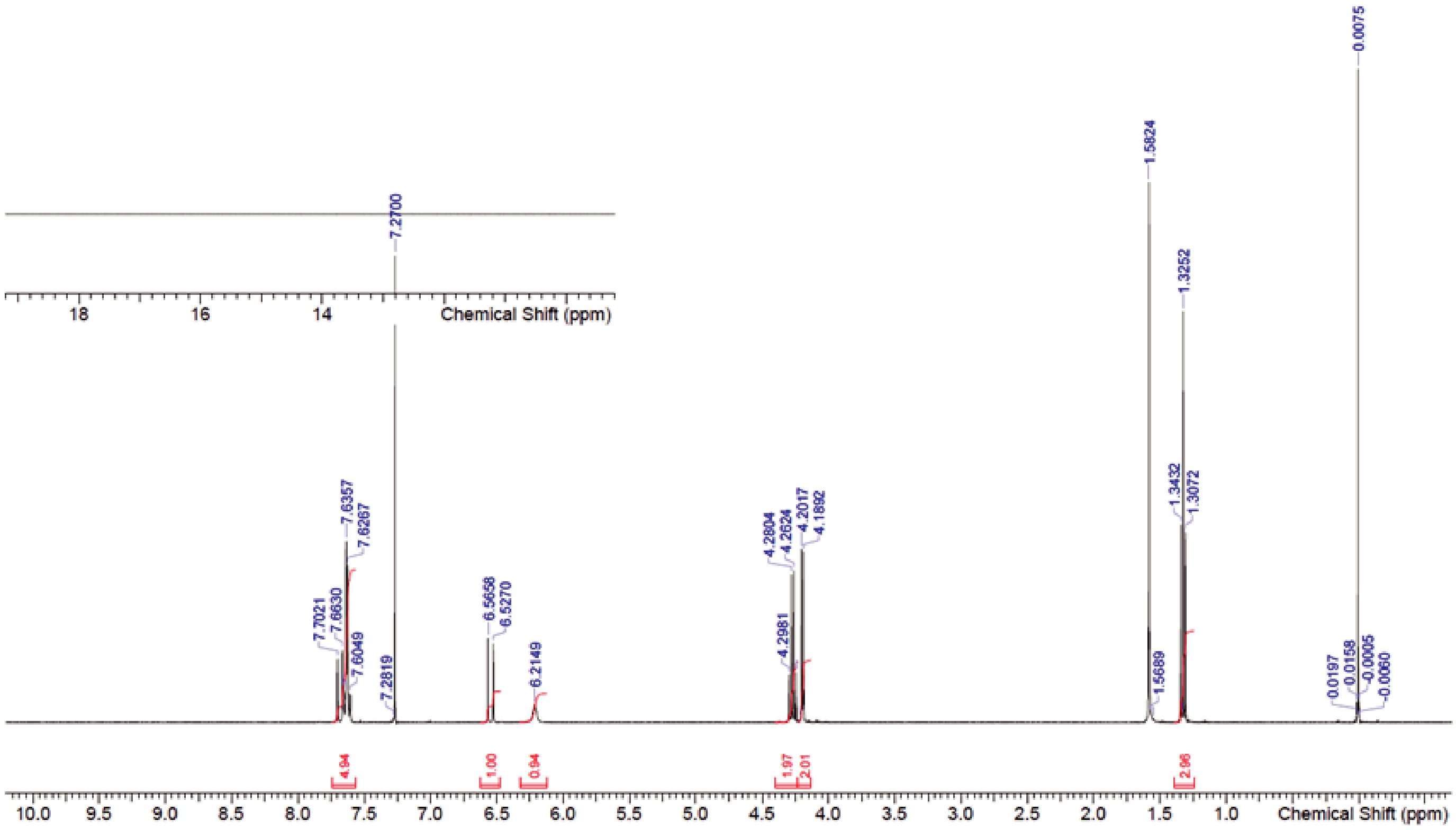

Ethyl 2-[[(E)-3-[4-(trifluoromethyl)phenyl]prop-2-enoyl]amino]acetate (4) ^{13}C NMR (100 MHz, DMSO-d_6)

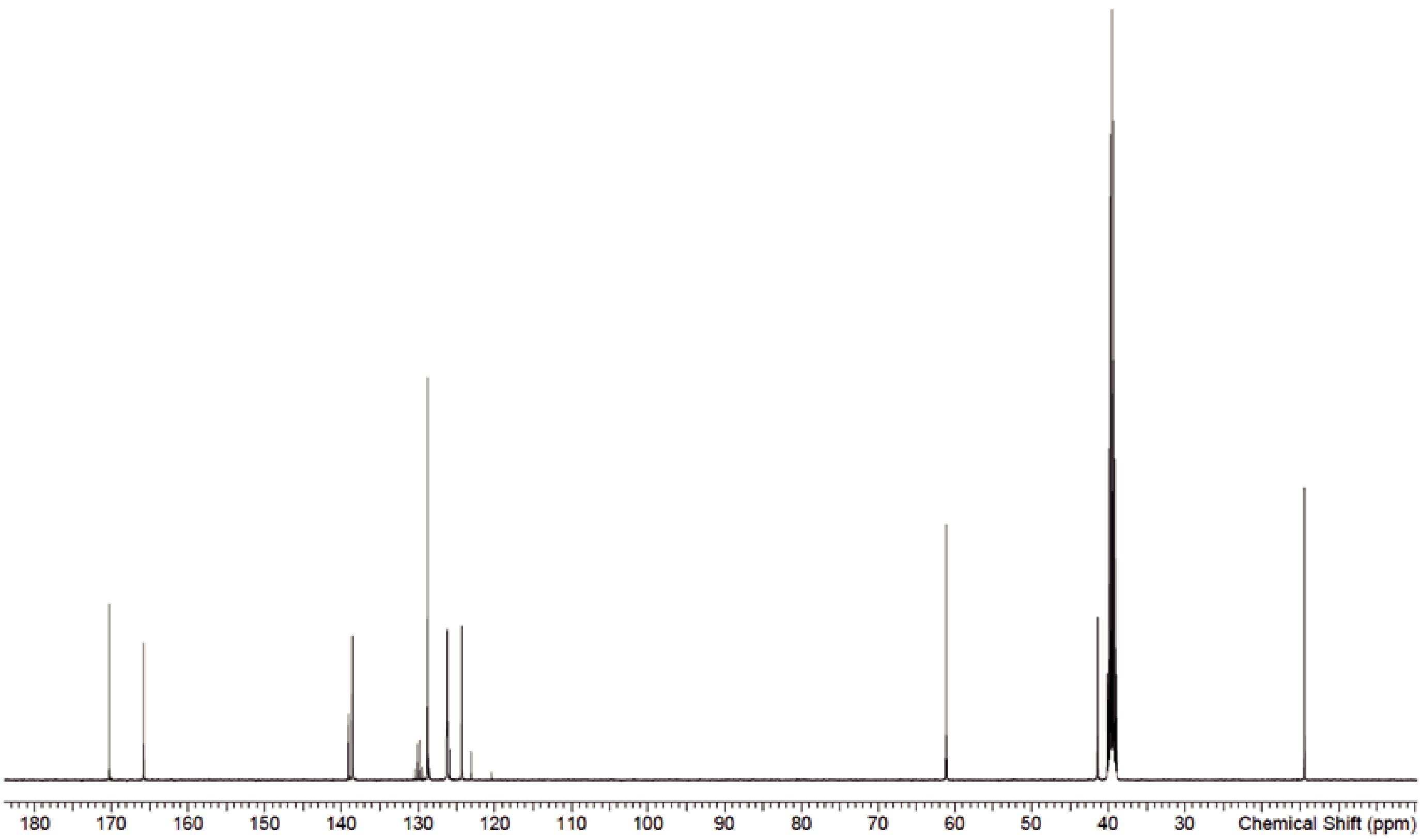

2-[[(E)-3-[4-(trifluoromethyl)phenyl]prop-2-enoyl]amino]acetic acid (5)

A mixture of ethyl 2-[[(E)-3-[4-(trifluoromethyl)phenyl]prop-2-enoyl]amino]acetate (40.5 g, 134 mmol, 1.00 eq) and 1 mol/L NaOH (270 mL, 270 mmol, 2.00 eq) in THF (100 mL) was stirred for 2 h at rt. Then 1 mol/L HCl was added to neutralize, precipitated solid was separated with filtration. The filtered compound was washed with water and dried over. White solid, yield 34.5 g (126 mmol, 94 %).

^{1}H NMR (400 MHz, DMSO-d_6): δ [ppm] = 3.91 (d, J = 5.8 Hz, 2H), 6.87 (d, J = 16.0 Hz, 1H), 7.54 (d, J = 16.0 Hz, 1H), 7.74-7.86 (m, 4H), 8.52 (t, J = 5.8 Hz, 1H), 12.66 (br s, 1H). ^{13}C NMR (100 MHz, DMSO-d_6): δ [ppm] = 41.4, 120.4, 123.1, 124.6, 125.1, 126.2, 128.7, 129.9, 138.4, 139.1, 165.7, 171.7. HRMS (ESI): m/z = 274.0686 (calcd. 274.0683 for $C_{12}H_{11}F_3NO_3$ $[M+H]^+$).

2-[[(E)-3-[4-(trifluoromethyl)phenyl]prop-2-enoyl]amino]acetic acid (5) ^{1}H NMR (400 MHz, DMSO-d_6)

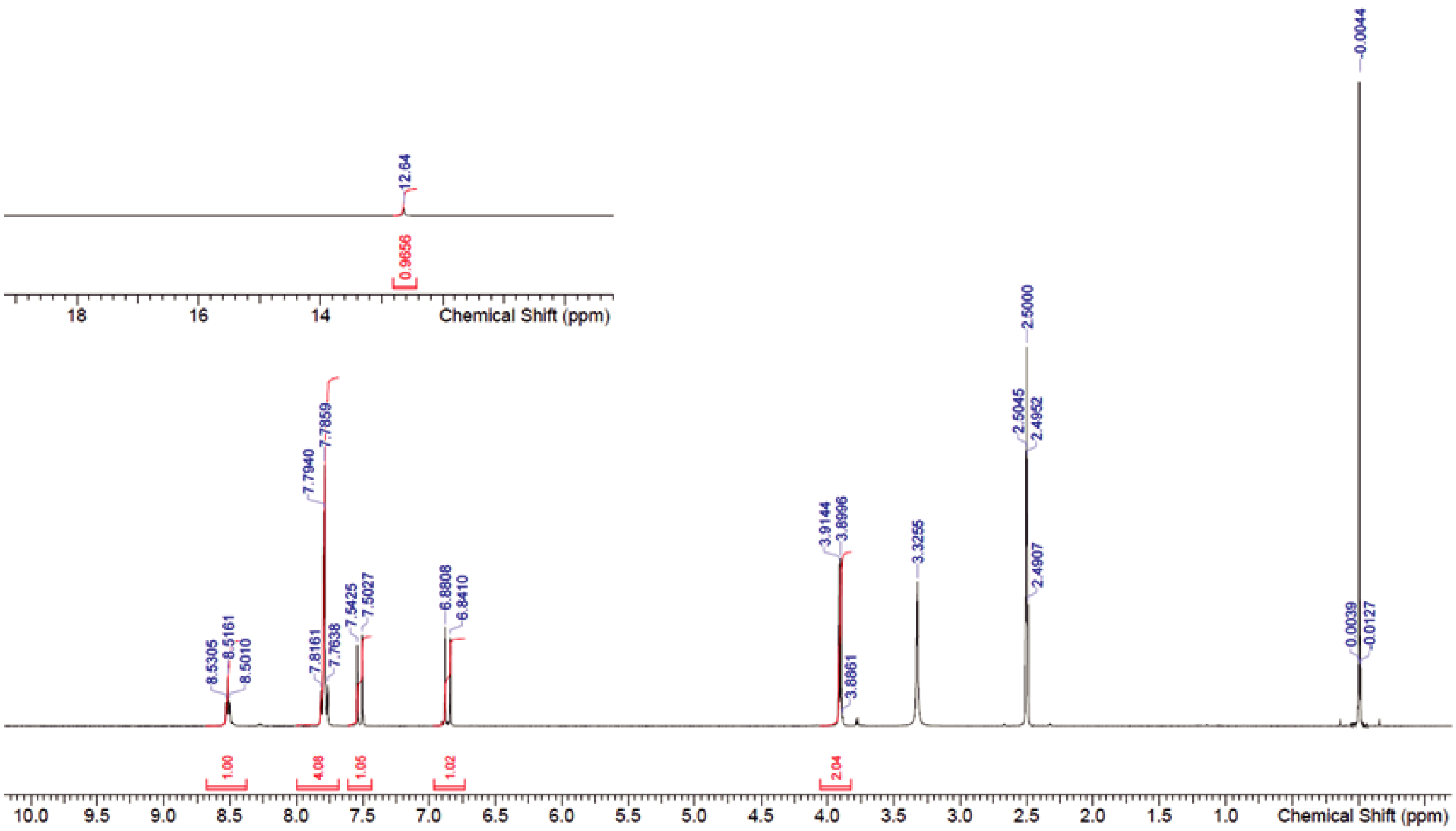

2-[[(E)-3-[4-(trifluoromethyl)phenyl]prop-2-enoyl]amino]acetic acid (5) ^{13}C NMR (100 MHz, DMSO-d_6)

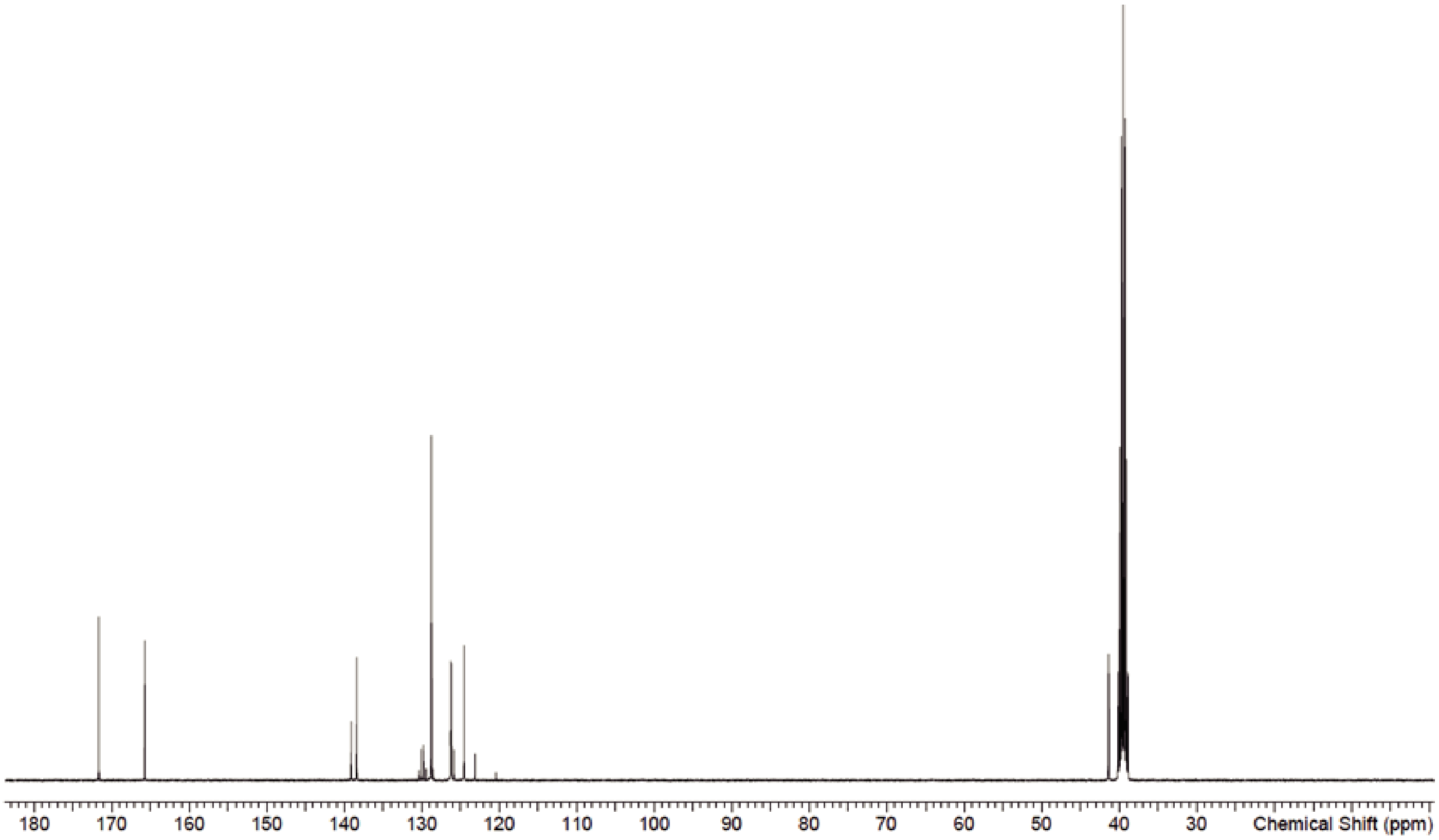

(E)-N-[2-oxo-2-(3-oxopiperazin-1-yl)ethyl]-3-[4-(trifluoromethyl)phenyl]prop-2-enamide (compound 3)

2-[[(E)-3-[4-(trifluoromethyl)phenyl]prop-2-enoyl]amino]acetic acid (51.0 mg, 0.19 mmol, 1.00 eq), piperazin-2-one (19 mg , 0.19 mmol, 1.02 eq), DIPEA(0.10 mL, 0.60 mmol, 3.00 eq) and HATU(78 mg, 0.21 mmol, 1.10 eq) were added to DMF (1 mL). The mixture was stirred for 1h at rt. Then, A saturated solution of NH_4Cl and $CHCl_3$ were added, the organic layer was separated and the aqueous layer was extracted with $CHCl_3$. The combined organic layers were dried (Na_2SO_4), filtered and concentrated in vacuo. The crude product was purified by HPLC (column :Xbridge C18, Φ30mm*50mm 5μm, solvent : A: 10mM, Ammonium Carbonate, B: CH_3CN, flow rate : 50 ml/min, Gradient 20-50% 3min). White solid, yield 62.0 mg (0.17 mmol, 93 %).

^{1}H NMR (400 MHz, $CDCl_3$): δ [ppm] = 3.43-3.55 (m, 2H), 3.72 (t, J = 5.6 Hz, 1H,), 3.90 (t, J = 5.6 Hz, 1H), 4.16 (s, 1H), 4.25 (dd, J = 4.2/12.6 Hz, 2H), 4.32 (s, 1H), 6.52 (m, 1H), 6.59 (dd, J = 2.0/13.6 Hz, 1H), 6.70-6.85 (m, 1H), 7.59-7.68 (m, 4H), 7.67 (dd, J = 2.6/13.6 Hz, 1H). ^{13}C NMR (100 MHz, DMSO-d$_6$): δ [ppm] = 41.3, 41.7, 46.2, 47.7, 120.6, 123.3, 124.7, 126.0, 126.4, 129.0, 130.1, 138.7, 139.2, 166.1, 167.1, 167.9. HRMS (ESI): m/z = 356.1217 (calcd. 356.1215 for $C_{16}H_{17}F_3N_3O_3$ [M+H]$^+$). Purity (HPLC): 96.6 %.

Compound 3 ^{1}H NMR (400 MHz, $CDCl_3$)

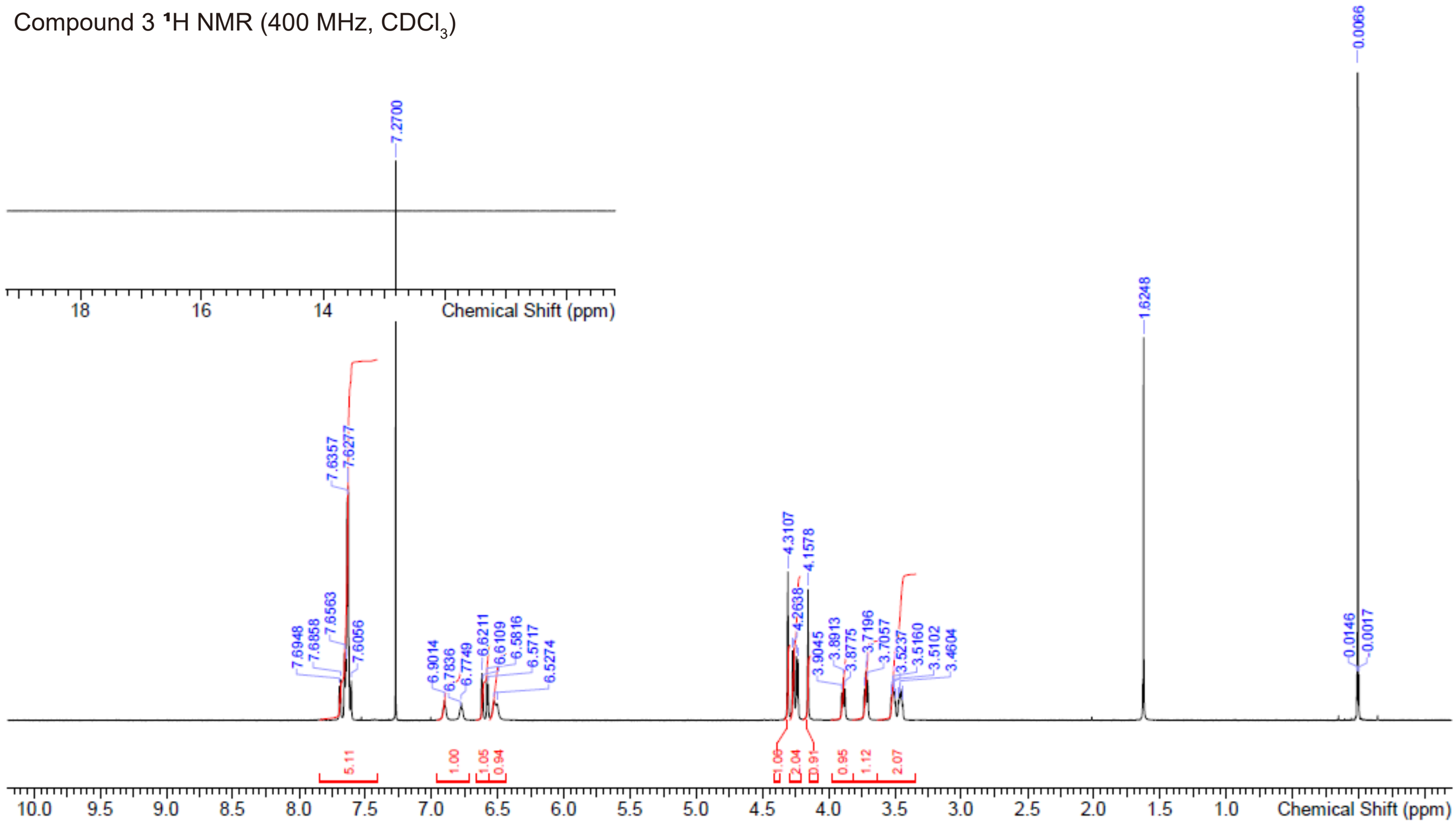

Compound 3 ^{13}C NMR (100 MHz, DMSO-d$_6$)

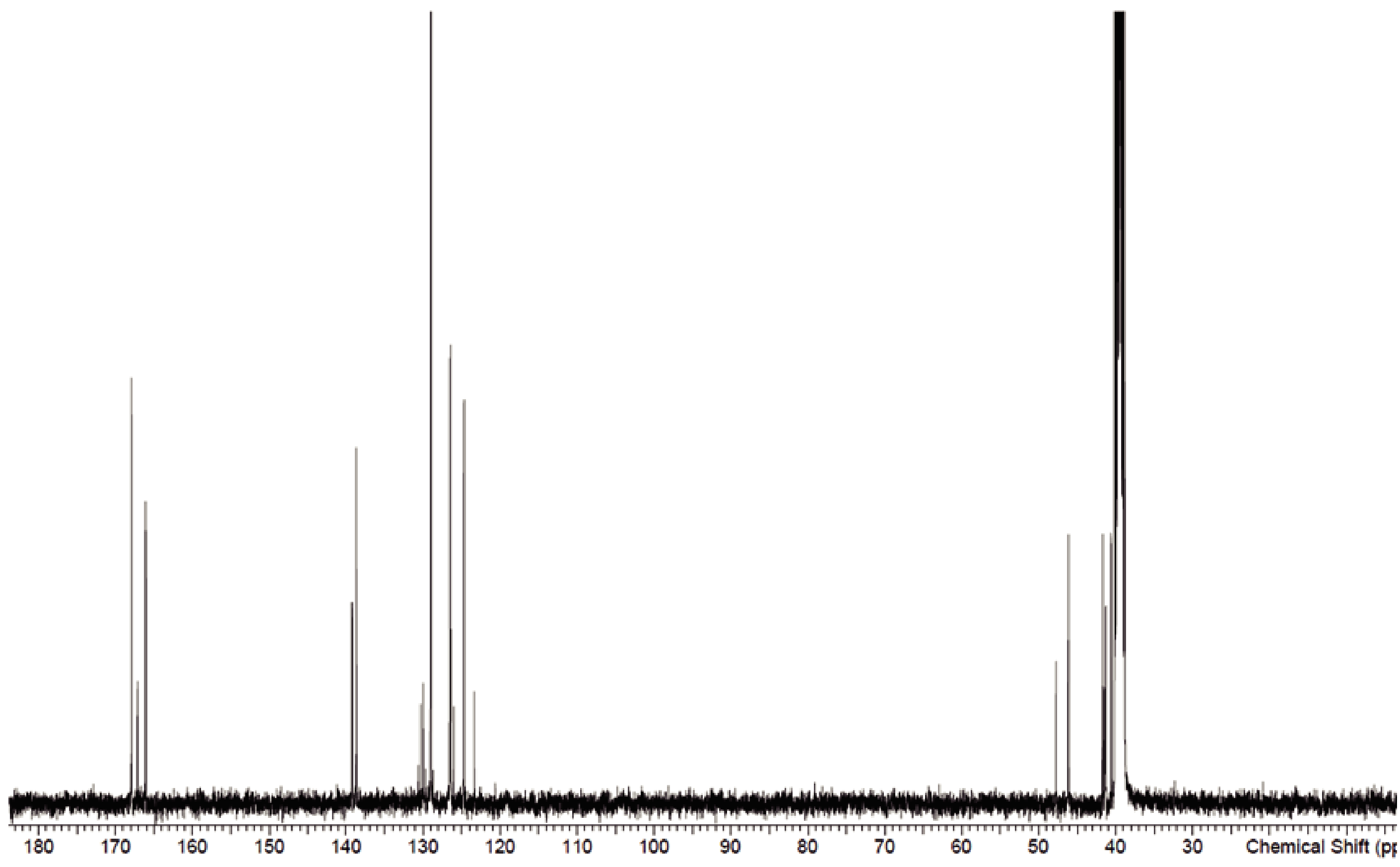

Supplement: Supplementary file 2 [file 42003_2026_10535_MOESM2_ESM.pdf]
