# Supplementary material for: Structure-guided development of a potent human B0AT1 inhibitor effective in a mouse model of phenylketonuria
Source: Commun Biol. 2026 Jun 25;9:1350. doi: 10.1038/s42003-026-10535-y (PMC13669362; doi:10.1038/s42003-026-10535-y)
Supplement: Supplementary file 3 [file 42003_2026_10535_MOESM3_ESM.pdf]

Description of Additional Supplementary Files

File name: Supplementary Data 1

Description: Kinetic parameters of d5-phenylalanine uptake by hB^0AT1 in the presence of compound 3. Each data represents the mean ± SEM (n = 3, technical replicates).

File name: Supplementary Data 2

Description: Kinetic parameters of compound 3 in the crude membrane expressing B^0AT1. Each data represents the mean ± SEM (n = 3, technical replicates).

File name: Supplementary Data 3

Description: Plasma concentration of compound 3 in male Pah^{enu2} mice. Plasma concentration of compound 3 after administration orally at a dose of 30 mg/kg in male Pah^{enu2} mice. Each data represents the mean ± SD (n = 8, biological replicates).

File name: Supplementary Data 4

Description: Source data underlying the graphs in the figures and tables
